# Epigenetics and plant hormone dynamics: a functional and methodological perspective

**DOI:** 10.1093/jxb/erae054

**Published:** 2024-02-19

**Authors:** Jiri Rudolf, Lucia Tomovicova, Klara Panzarova, Jiri Fajkus, Jan Hejatko, Jan Skalak

**Affiliations:** Mendel Centre for Plant Genomics and Proteomics, CEITEC - Central European Institute of Technology, Masaryk University, Kamenice 753/5, CZ-62500 Brno, Czech Republic; Department of Cell Biology and Radiobiology, Institute of Biophysics of the Czech Academy of Sciences, CZ-61265 Brno, Czech Republic; National Centre for Biomolecular Research, Faculty of Science, Masaryk University, Kamenice 753/5, CZ-62500 Brno, Czech Republic; Mendel Centre for Plant Genomics and Proteomics, CEITEC - Central European Institute of Technology, Masaryk University, Kamenice 753/5, CZ-62500 Brno, Czech Republic; National Centre for Biomolecular Research, Faculty of Science, Masaryk University, Kamenice 753/5, CZ-62500 Brno, Czech Republic; Photon Systems Instruments, Prumyslova 470, CZ-664 24 Drasov, Czech Republic; Mendel Centre for Plant Genomics and Proteomics, CEITEC - Central European Institute of Technology, Masaryk University, Kamenice 753/5, CZ-62500 Brno, Czech Republic; Department of Cell Biology and Radiobiology, Institute of Biophysics of the Czech Academy of Sciences, CZ-61265 Brno, Czech Republic; National Centre for Biomolecular Research, Faculty of Science, Masaryk University, Kamenice 753/5, CZ-62500 Brno, Czech Republic; Mendel Centre for Plant Genomics and Proteomics, CEITEC - Central European Institute of Technology, Masaryk University, Kamenice 753/5, CZ-62500 Brno, Czech Republic; National Centre for Biomolecular Research, Faculty of Science, Masaryk University, Kamenice 753/5, CZ-62500 Brno, Czech Republic; Mendel Centre for Plant Genomics and Proteomics, CEITEC - Central European Institute of Technology, Masaryk University, Kamenice 753/5, CZ-62500 Brno, Czech Republic; National Centre for Biomolecular Research, Faculty of Science, Masaryk University, Kamenice 753/5, CZ-62500 Brno, Czech Republic; University of South Bohemia, Czech Republic

**Keywords:** Abscisic acid, auxin, cytokinins, epigenetics, ethylene, gibberellins, histone modifications

## Abstract

Plant hormones, pivotal regulators of plant growth, development, and response to environmental cues, have recently emerged as central modulators of epigenetic processes governing gene expression and phenotypic plasticity. This review addresses the complex interplay between plant hormones and epigenetic mechanisms, highlighting the diverse methodologies that have been harnessed to decipher these intricate relationships. We present a comprehensive overview to understand how phytohormones orchestrate epigenetic modifications, shaping plant adaptation and survival strategies. Conversely, we explore how epigenetic regulators ensure hormonal balance and regulate the signalling pathways of key plant hormones. Furthermore, our investigation includes a search for novel genes that are regulated by plant hormones under the control of epigenetic processes. Our review offers a contemporary overview of the epigenetic–plant hormone crosstalk, emphasizing its significance in plant growth, development, and potential agronomical applications.

## Teaser

We summarize advances in understanding the dynamic interplay between the key plant hormones auxin, cytokinins, ethylene, abscisic acid, and gibberellins, and epigenetic mechanisms, highlighting diverse methodologies employed in recent research.

## Introduction

The intricate interplay between plant hormones and epigenetic regulation stands at the forefront of modern plant biology, shaping our understanding of how plants dynamically respond to environmental cues and orchestrate their growth and development. The perennial question of which factor—plant hormones or epigenetic regulation—exerts a greater influence over plant physiology and adaptation has stimulated extensive scientific discourse. Plant hormones, as quintessential mediators of growth and stress responses, have long been recognized for their pivotal roles in modulating various aspects of plant life. On the other hand, different types of epigenetic regulation, such as modifications to DNA and associated proteins in chromatin, without altering the genetic code, have emerged as powerful mechanisms to reprogram gene expression during development and in response to environmental stimuli, and shape phenotypic diversity. In this introductory exploration, we embark on a journey to unravel the nuanced relationship between these two fundamental aspects of plant biology, aiming to shed light on their importance in shaping the detailed patterns of plant traits and responses. Through a methodological lens, we summarize our recent knowledge on the mechanisms underlying the crosstalk between hormonal regulation and epigenetic regulation. While all plant hormones contribute to the overall regulation of plant growth and responses to environmental cues, they differ in their specific functions and the processes they predominantly influence. In this review, our focus is on the five ‘classical’ plant hormones auxin, cytokinins, ethylene, abscisic acid (ABA), and gibberellins (GAs) that act in fundamental developmental processes (Kende and Zeevaart, 1997). Hormones such as jasmonic acid (JA), salicylic acid (SA), and strigolactones, on the other hand, are more closely associated with stress responses and defence mechanisms, opening up another voluminous chapter concerning the role of the abiotic/biotic stress responses in epigenetic processes exceeding the scope of our review.

## Hormone–epigenetic synergy: illuminating plant processes through common methodologies

**Fig. 1.**
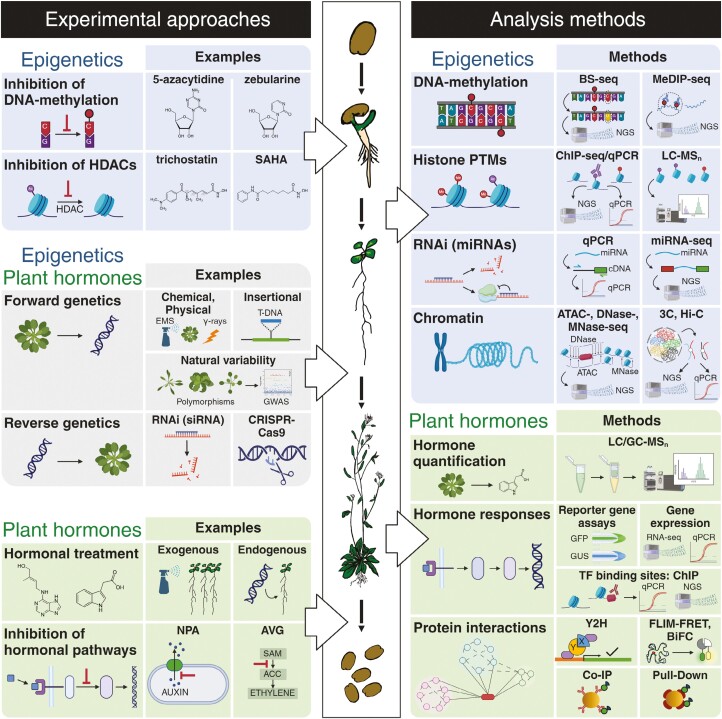
Schematic pipeline of methods for studying epigenetic–hormonal crosstalk. The conventional experimental approaches in plant hormone biology and epigenetics are shown on the left side, such as inhibition of DNA methylation and histone deacetylation, exogenous hormonal treatments, inhibition of hormonal pathways, and forward/reverse genetic approaches. The right side presents various analytical techniques and methods used to explore these areas of research, including high-throughput sequencing (such as RNA-seq, ChIP-seq, BS-seq, MeDIP-seq, and ATAC-seq), spatial chromatin organization methods (such as 3C and Hi-C), mass spectrometry-based techniques (LC/GC-MS_n_), and advanced imaging techniques for studying reporter gene lines and protein–protein interactions by BiFC or FLIM-FRET, complementing with *in vitro* protein–protein interaction methods (such as Y2H, co-IP, and pull-down). 3C, chromosome conformation capture; ACC, 1-aminocyclopropane-1-carboxylate; ATAC-seq, assay for transposase-accessible chromatin using sequencing; AVG, aminoethoxyvinylglycine; BiFC, bimolecular fluorescence complementation; BS-seq, bisulfite sequencing; ChIP-seq, ChIP followed by sequencing; co-IP, co-immunoprecipitation; DNase-seq, DNase I hypersensitive sites sequencing; EMS, ethyl methanesulfonate; FLIM-FRET, fluorescence lifetime imaging-Förster resonance energy transfer; GFP, green fluorescent protein; GUS, β-glucuronidase; GWAS, genome-wide association studies; HDACs, histone deacetylases; Hi-C, high-throughput chromosome conformation capture; MeDIP-seq, methylated DNA immunoprecipitation sequencing; miRNA-seq, miRNA sequencing; MNase-seq, micrococcal nuclease sequencing; NGS, next-generation sequencing; NPA, *N*-1-naphthylphthalamic acid; PTM, post-translational modification; qPCR, quantitative PCR; RNA-seq, RNA sequencing; SAM, *S*-adenosylmethionine; SAHA, suberoylanilide hydroxamic acid; TF, transcription factor; Y2H, yeast two-hybrid. Created with BioRender.com.

Initially, we summarize cutting-edge technologies that are widely employed for advanced understanding of the role of plant hormones in epigenetic processes (Fig. 1). One of these frequently used techniques is ChIP followed by sequencing (ChIP-seq) that has enabled researchers to delineate hormone-responsive genomic regions marked by histone modifications, providing insights into how DNA-binding proteins, such as transcription factors or chromatin modifiers, interact with DNA sequences in the context of chromatin (Kaufmann *et al.*, 2010). The principle of immunoprecipitation can also be used for the study of methylated DNA—methylated DNA immunoprecipitation sequencing (MeDIP-seq; Wardenaar *et al.*, 2013; Cortijo *et al.*, 2014). Alternatively, bisulfite sequencing has proven invaluable in deciphering the impact of plant hormones on DNA methylation patterns, unveiling hormone-responsive methylome dynamics and their implications for gene expression (Foerster and Mittelsten Scheid, 2010).

In connection to methylation and DNA-binding proteins, it is often relevant to evaluate the chromatin accessibility status. Several methods were developed utilizing open chromatin hypersensitivity to nucleases or nucleosome hindrance in nuclease digestion—DNase I-hypersensitive sites sequencing (DNase-seq) and micrococcal nuclease digestion with deep sequencing (MNase-seq; Cumbie *et al.*, 2015; Zhang and Jiang, 2015; Pajoro *et al.*, 2018; Zhao *et al.*, 2020). Alternatively, open chromatin is exposed to Tn*5* transposase tagging, which is utilized in assay for transposase-accessible chromatin using sequencing (ATAC-seq; Bajic *et al.*, 2018; X. Wang *et al.*, 2022). These methods provide crucial information about chromatin accessibility; however, they do not disclose spatial information and higher chromatin structures. For such a purpose, high-throughput chromatin conformation capture (Hi-C) could be mentioned as a powerful tool enabling depiction of all-to-all chromatin proximity and thus potentially interchromatin interactions (Feng *et al.*, 2014). More recently, Hi-C was also combined with INTACT (isolation of nuclei tagged in specific cell types), resulting in an INT-Hi-C method allowing cell type-specific chromatin conformation capture (Yadav *et al.*, 2021). RNA sequencing (RNA-seq) data have unveiled the hormone-induced transcriptomic alterations associated with epigenetic modifications, offering a holistic view of hormone-mediated regulatory networks. Using transgenic and mutant studies, coupled with clustered regularly interspaced palindromic repeats (CRISPR)-based epigenome editing techniques, researchers were able to dissect hormone signalling pathways and examine their impact on epigenetic marks, elucidating their roles in developmental plasticity and stress responses (Tyagi *et al.*, 2022).

Mass spectrometry is an essential method to investigate histone variants and their post-translational modifications and modifiers (Lochmanová *et al.*, 2019; Kuchaříková *et al.*, 2021, 2022). Combining the results of the aforementioned techniques together with state-of-the-art bioinformatics approaches has deepened our understanding of hormone-associated epigenetic phenomena regulating gene expression of key factors of plant development (Chenarani *et al.*, 2021). The integration of multi-omics datasets has enabled the identification of novel hormone-responsive epigenetic regulators, unravelling previously uncharted dimensions of hormone signalling cascades.

In summary, the above-mentioned techniques provide a cornerstone methodology of almost all studies discussed below, reflecting their pivotal role in advancing research of epigenetics and plant hormones. Novel techniques, such as chemical down-regulation of histone modifications using inhibitors of histone modifiers in combination with genetic tools, have potential to extend our knowledge about the role of epigenetic regulation of plant hormone action, yet these approaches are still in their infancy. For instance, Wei *et al.* (2022) performed transcriptomic profiling of plants treated by the histone deacetylation inhibitor (HDACi) trichostatin A, identifying that several genes promoting auxin and GA signalling were down-regulated upon the treatment. Another HDACi, suberoylanilide hydroxamic acid, induces strong hyperacetylation of histones H3 and H4 in roots followed by up-regulation of genes involved in biosynthesis of ethylene, ABA, and GAs (Patanun *et al.*, 2017). While the influence of epigenetic status on hormonal responses undoubtedly exists, deciphering this interaction remains challenging due to the complex molecular network behind those processes. In spite of these challenges, we embark on a comprehensive review of this complex topic below.

## Auxin

Auxin plays a central role in orchestrating a wide array of developmental processes and physiological responses in plants, such as cell elongation, division, and differentiation, which collectively contribute to organ formation, root and shoot development, and vascular tissue differentiation (Han *et al.*, 2021; Verma *et al.*, 2021; Li *et al.*, 2022). The realm of auxin biosynthesis and transport is intricate and abundant, to the extent that providing a comprehensive description necessitates a dedicated review. Thus, we encourage exploring additional reviews to acquire a more in-depth understanding of auxin regulation (Casanova-Sáez *et al.*, 2021; Solanki and Shukla, 2023). In this context, our focus will be directed solely towards examining the epigenetic factors affecting auxin metabolism and signalling, and techniques that support the observations.

### Auxin levels under epigenetic control

**Fig. 2.**
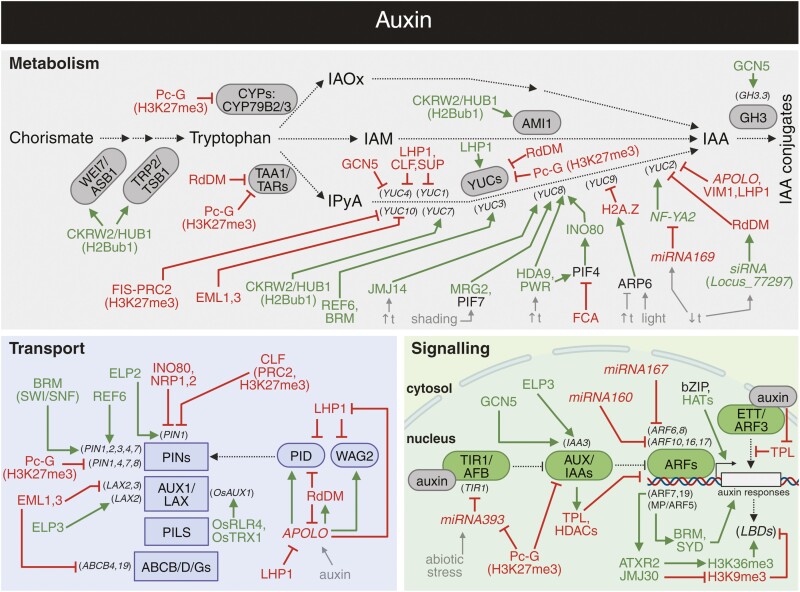
Overview of crosstalk between auxin and epigenetic regulation. The interplays between auxin metabolism (grey), transport (blue), signalling (green), and epigenetic mechanisms. Arrows in dark green and blunt arrows in red denote the specific positive and negative effects, respectively. See the main text for a detailed description of the illustrated crosstalk. CYP79B2, 3, CYTOCHROME P450 FAMILY 79 SUBFAMILY B POLYPEPTIDE 2, 3; IAA, indole-3-acetic acid; IAM, indole-3-acetamide; IAOx, indole-3-acetaldoxime; IPya, indole-3-pyruvic acid; AMI1, AMIDASE 1; Pc-G, Polycomb-group proteins; PIF7, PHYTOCHROME-INTERACTING FACTOR 7; SUP, SUPERMAN; SWI/SNF, SWITCH/SUCROSE NON-FERMENTING; TRP2/TSB1, TRYPTOPHAN BIOSYNTHESIS 2/TRYPTOPHAN SYNTHASE BETA SUBUNIT 1; WEI7/ASB1, WEAK ETHYLENE INSENSITIVE 7/ANTHRANILATE SYNTHASE BETA SUBUNIT 1; ↑t, increased temperature; ↓t, decreased temperature. Abbreviations not stated here are explained in the main text. Created with BioRender.com.

Various epigenetic mechanisms impact the transcription of genes responsible for auxin biosynthesis (Do *et al.*, 2019; Mateo-Bonmatí *et al.*, 2019; Casanova-Sáez *et al.*, 2021; J.-L. Wang *et al.*, 2022; Solanki and Shukla, 2023). Comprehensive whole-genome tiling arrays indicated the repressive role of H3K27me3 over larger genomic regions for genes related to auxin metabolism, such as *YUCCA* (*YUC*), *CYTOCHROME P450* (*CYP*), and *TRYPTOPHAN AMINOTRANSFERASE 1/TRYPTOPHAN AMINOTRANSFERASE-RELATED* (*TAA1/TAR)* genes (Fig. 2; Lafos *et al.*, 2011; He *et al.*, 2012). Considering this modification, it is predominantly connected to the Polycomb group pathway that epigenetically represses gene expression. There are two Polycomb repressive complexes (PRCs)—PRC2 is a histone writing complex depositing H3K27me3, which is recognized by PRC1 (Baile *et al.*, 2022; Godwin and Farrona, 2022). Subsequently, ubiquitin is deposited on H2A as a mark of gene repression and the locus is further repressed by other machineries (Baile *et al.*, 2022; Godwin and Farrona, 2022). PRC2 component FERTILIZATION INDEPENDENT SEED (FIS) was shown to control *YUC10* expression during endosperm development (Figueiredo *et al.*, 2015), which is likely to be repressed by EMSY-Like (EML) Tudor/Agenet histone readers EML1 and EML3 during the same process (Milutinovic *et al.*, 2019).

A plant-specific component of PRC1, LIKE HETEROCHROMATIN PROTEIN 1 (LHP1), is involved in Polycomb repression presumably by interaction with PRC2 and PRC1 components (Derkacheva *et al.*, 2013; Wang *et al.*, 2014; Zhou *et al.*, 2017). LHP1 is often considered to be a PRC1 subunit, but, interestingly, LHP1 can act both as a repressor and an activator (Wang *et al.*, 2016; Feng and Lu, 2017). Using ChIP-qPCR, LHP1 was shown to bind to several *YUC* gene loci in seedlings (Rizzardi *et al.*, 2011). This binding is partially auxin dependent, thus pointing to specific feedback mechanisms. The corresponding *lhp1* mutant shows greatly diminished auxin levels in shoots and *YUC* gene expression in whole seedlings, suggesting a positive role for *LHP1* in auxin biosynthesis. On the other hand, LHP1 was suggested to negatively regulate *YUC1* and *YUC4* expression during floral organ development in interaction with the PRC2 component CURLY LEAF (CLF) and the transcription factor SUPERMAN (Xu *et al.*, 2018). Thus, LHP1 seems to function in a development- and tissue-specific context.

*AUXIN-REGULATED PROMOTER LOOP* (*APOLO*) is another epigenetic factor that is an important regulator of both auxin biosynthesis and transport (Ariel *et al.*, 2014, 2020; Feng and Lu, 2017). *APOLO* recognition by chromatin isolation by RNA purification (ChIRP) sequencing revealed that in the context of auxin biosynthesis, *APOLO* links LHP1 with DNA methylation via interaction with the methylcytosine-binding protein VARIANT IN METHYLATION 1 (VIM1) (Fig. 1; Fonouni-Farde *et al.*, 2022). This complex/module is capable of both RNA-directed DNA methylation (RdDM) and Polycomb-mediated repression of the *YUC2* locus. Furthermore, *YUC2* was shown to be targeted by small RNAs, such as siRNAs and miRNAs, both indirectly and directly (Gyula *et al.*, 2018). The *miRNA169* represses a subunit of the NUCLEAR FACTOR Y (NF-Y) complex *NF-YA2* which regulates *YUC2* expression. Considering other non-coding RNAs, the *YUC2* promoter itself contains *Locus_77297*-transcribed siRNA, which is thought to direct RdDM in response to ambient temperature fluctuations (Gyula *et al.*, 2018). Although mechanistically not confirmed, investigation of the DNA methyltransferases DOMAINS REARRANGED METHYLTRANSFERASE 1/2 (DRM1/2) and CHROMOMETHYLASE 3 (CMT3) points to their involvement in RdDM-mediated regulation of *YUC2* since the *drm1 drm2 cmt3* mutant exhibits elevated levels of *YUC2* in leaves followed by corresponding changes in DNA methylation observed by whole-genome bisulfite sequencing (Forgione *et al.*, 2019, 2022). The RdDM regulation does not seem to be unique for the regulation of *YUC2*. By analysing the association of RNA polymerase V (RNA Pol V) with chromatin as done by W. Liu *et al.* (2018), Markulin *et al.* (2021) propose that RdDM-based mechanisms control the expression of many auxin biosynthetic genes such as *TAA1/TAR* genes (*TAA1*, *TAR1*, and *TAR2*) and *YUC* genes (*YUC1*, *YUC2*, *YUC5*, and *YUC10*), but also auxin transport and signalling genes.

As a counterweight of Polycomb repression, H3K27me3 can be demethylated by JUMONJI DOMAIN-CONTAINING (JMJ) protein to either H3K27me2 or even further. Using ChIP-seq, Cui *et al.* (2016) observed hypermethylation on the *YUC3* locus in the mutant of *JMJ12/RELATIVE OF EARLY FLOWERING 6* (*REF6*). In line with that, C. Li *et al.* (2016) provided data showing that REF6 binds to the *YUC3* locus, demethylates corresponding histones, and, in concert, the chromatin architecture is adjusted by the chromatin-remodelling ATPase BRAHMA (BRM). *REF6* has several paralogues with similar activity—*EARLY FLOWERING 6* (*ELF6*) and *JMJ13*. Work on the triple mutant *ref6 elf6 jmj13* led to the conclusion that such regulation could be common for several *YUC* genes (*YUC3*, *YUC8*, and *YUC11*; Yan *et al.*, 2018). Concerning other members of the *JMJ* gene family with different substrate specificity, JMJ14 was shown to affect *YUC8* activation in response to higher temperature, suggesting the role of H3K4 demethylases in the auxin-related thermosensory response (Cui *et al.*, 2021). In conclusion, both H3K27 and H3K4 JMJ demethylases regulate *YUC* expression, affecting many developmental and environmental responses through the modulation of auxin biosynthesis (Cui *et al.*, 2021; Yamaguchi, 2021).

Similarly to histone methylation, histone acetylation also mediates changes in plant chromatin structure and gene expression (Boycheva *et al.*, 2014). Histone acetylation levels are regulated by HISTONE ACETYLTRANSFERASES (HATs) and HISTONE DEACETYLASES (HDACs) which were shown to affect expression of auxin metabolism-related genes (J.-L. Wang *et al.*, 2022). Although HDACs are often presented as repressive components of epigenetic regulatory networks, HDA9 was shown to positively regulate *YUC8* expression during thermomorphogenesis (van der Woude *et al.*, 2019). Using different HDAC inhibitors (sodium butyrate and trichostatin) in combination with RNA-seq analysis (Fig. 1), HDA9 together with its interactor POWERDRESS (PWR) were shown to mediate histone deacetylation at the *YUC8* locus, which leads to subsequent depletion of the H2A.Z histone variant and binding of transcription factor PHYTOCHROME INTERACTING FACTOR 4 (PIF4; Kim *et al.*, 2016; Tasset *et al.*, 2018; van der Woude *et al.*, 2019). Further research revealed that PIF4 is recruiting the INOSITOL AUXOTROPHY 80 (INO80) chromatin-remodelling complex to evict H2A.Z at PIF4 target sequences, including *YUC8* (Xue *et al.*, 2021). Consequently, INO80 directly interacts with the COMPASS-like complex mediating H3K4me3 deposition, which results in gene activation (Xue *et al.*, 2021). Moreover, the binding of PIF4 to the *YUC8* promoter can be attenuated by interaction with FLOWERING CONTROL LOCUS A (FCA) which causes dissociation of PIF4 from the *YUC8* promoter while inducing changes in histone modifications in the *YUC8* locus (Lee *et al.*, 2014). Functionally, FCA serves as an RNA-binding 3'-end processing protein that interacts with the PRC2 component CLF (Macknight *et al.*, 1997; Sonmez *et al.*, 2011; Tian *et al.*, 2019). Furthermore, it is functionally connected with LYSINE-SPECIFIC DEMETHYLASE1/FLOWERING LOCUS D (FLD) and DICER-LIKE 3 (F. Liu *et al.*, 2007). Thus, FCA serves as a hub bringing together regulation by non-coding RNAs and histone modifications, especially in the context of Polycomb repression. Because of that, it can be expected that its role in regulation of auxin biosynthesis is not limited to PIF4 interaction.

Supporting the role of H2A.Z in auxin biosynthesis regulation, disruption of the H2A.Z deposition machinery by mutation in *ACTIN-RELATED PROTEIN 6* (*ARP6*) resulted in higher *YUC9* expression and altered auxin levels (Lee and Seo, 2017; van der Woude *et al.*, 2019). In addition to that, ARP6 also acts as a negative regulator of YUCs, especially *YUC9* expression during photomorphogenesis (Wei *et al.*, 2021). Such results are in accordance with the above-presented role of H2A.Z in thermomorphogenesis as both thermo- and photomorphogenesis are controlled by similar machineries (Delker *et al.*, 2022; P. Wang *et al.*, 2022).

In contrast to the above-described HDA9–PWR–PIF4-mediated increase in *YUC8* expression, acetylation plays an opposite role in *YUC8* transcription during shade avoidance (Peng *et al.*, 2018). In the shade, PIF7 interacts with a reader of positive histone methylation MORF RELATED GENE 2 (MRG2) and induces *YUC8* (and possibly also *YUC9*) expression (Xu *et al.*, 2014; Peng *et al.*, 2018). This induction correlates with histone hyperacetylation observed by ChIP-qPCR in various chromatin regions of *YUCCA8*, potentially associated with MRG2-related recruitment of HATs (Xu *et al.*, 2014; Peng *et al.*, 2018). This underlines the importance of evaluation of the effects in the specific context of the gene and developmental process even though such processes can be regulated by similar machineries (in this case PIFs). The outcomes can vary for the same modification in the same locus, depending on the observed process. In part, this could be explained by different combinations of observed acetylation patterns and different position of the modifications in the locus (Peng *et al.*, 2018; van der Woude *et al.*, 2019). Moreover, several HDACs, such as HDA6, could be expected to affect mRNA processing, poly(A) tail length, and thus RNA stability (Lin *et al.*, 2020).

Histone acetylation also seems to play a role in auxin homeostatic mechanisms controlled by auxin signalling. Using classic quantitative reverse transcription–PCR (RT–qPCR), Weiste and Dröge-Laser (2014) observed that the expression of *GRETCHEN HAGEN 3* (*GH3.3*), encoding an auxin conjugase, increases after inhibition of HDACs by trichostatin A. *GH3.3* expression is also less responsive to external treatment with auxins after γ-butyrolactone application, which inhibits GENERAL CONTROL NON-REPRESSIBLE 5/HISTONE ACETYLTRANSFERASE OF THE GNAT FAMILY 1 (GCN5/HAG1; Q. Chen *et al.*, 2010). Mechanistically, this regulatory loop seems to be dependent on basic leucine zipper (bZIP) transcription factors which are able to recruit HATs, like the above-mentioned GCN5, with a positive effect on expression of auxin-related genes such as *GH3.3* (Weiste and Dröge-Laser, 2014). Nevertheless, GCN5 is hypothesized to negatively regulate *YUC4* expression during pistil development, but it has to be mentioned that GCN5 has a global effect on plant development and that mutation in *GCN5* actually resulted in higher H3K9/K14 acetylation in the *YUC4* promoter region (Poulios and Vlachonasios, 2018). Therefore, the observed results of *gcn5* mutation or pharmacological inhibition of GCN5 function probably involve many indirect effects.

In addition to histone methylation and acetylation, ubiquitination is another chromatin modification controlling gene expression (Feng and Shen, 2014; March and Farrona, 2018). ChIP analysis showed that mutants in CYTOKININ INDUCED ROOT WAVING 2/HISTONE MONOUBIQUITINATION 1 (CKRW2/HUB1), a gene encoding an E3 ligase required for histone H2B monoubiquitination (H2Bub1), had typical auxin-deficient phenotypes in roots (Zhang *et al.*, 2021). These phenotypes were associated with lower expression of functional auxin biosynthetic genes including *YUC7*. ChIP analysis revealed defects in H2Bub1 within the coding regions of these genes, suggesting the role of H2Bub1 in the regulation of auxin biosynthesis (L. Wu *et al.*, 2015; Zhang *et al.*, 2021). However, the defects in H2Bub1 function might result in a pleiotropic phenotype as H2Bub1 regulates expression of genes involved in various developmental processes and plant hormonal networks—including auxins, cytokinins, and ABA (Bourbousse *et al.*, 2012; March and Farrona, 2018; Ma *et al.*, 2019; Zhang *et al.*, 2021). Moreover, gain-of-function mutation of histone modification enzymes might result in severe developmental defects with shifted circadian rhythms (Bourbousse *et al.*, 2012; Himanen *et al.*, 2012). As circadian oscillations regulate auxin signalling and auxin levels in plants (Covington and Harmer, 2007; Voß *et al.*, 2015), it is always necessary to refer to a certain time window when assaying the rate of auxin biosynthetic gene expression and endogenous auxin levels in lines deficient in histone modifiers.

### Epigenetic modulation of auxin transport

Once synthesized, auxin is transported to its final destination, creating local gradients within the tissue, which enable auxin-mediated organ development and tropic movements (Weijers *et al.*, 2006; Geisler, 2021; Han *et al.*, 2021). Auxin transporters include influx and efflux carriers localized on the plasma membrane but also endomembranes. AUXIN-RESISTANT 1/LIKE AUX1 (AUX1/LAX) are mostly plasma membrane-localized importers, whereas long-looped PIN-FORMED (PINs) are auxin exporters mostly localized in the plasma membrane, in contrast to short-looped PINs and PIN-LIKES (PILS) proteins, which are located to the endomembranes (Swarup and Bhosale, 2019; Zwiewka *et al.*, 2019; Geisler, 2021; Ung *et al.*, 2023). Last, but not least, several subgroups of ABC transporters (ABCBs, ABCDs, and ABCGs) were reported to participate in auxin export as well as import (Geisler *et al.*, 2017; Geisler, 2021).

As for auxin biosynthesis, the auxin transporters are widely regulated by histone modifiers, especially in connection to Polycomb repression. Several *PIN* loci (*PIN1*, *PIN4*, *PIN7*, and *PIN8*) show dynamic changes in H3K27me3 levels upon leaf differentiation or leaf-based callus formation (Lafos *et al.*, 2011; He *et al.*, 2012). Furthermore, the expression of *PIN1* was shown to be repressed by CLF, one of the PRC2 subunits, affecting development of lateral roots (Gu *et al.*, 2014).

Concerning the antagonistic processes, which result in lower H3K27me3 levels, the role of BRM and REF6 should be pointed out (C. Li *et al.*, 2015, 2016; Yang *et al.*, 2015; Wang *et al.*, 2019). The mutation in *REF6* leads to reduced expression of *PIN1*, *PIN2*, *PIN3*, *PIN4*, and *PIN7* (Wang *et al.*, 2019). *PIN1*, *PIN3*, and *PIN7* promoters are directly targeted by REF6, whereas expression of *PIN2* and *PIN4* is rather indirectly regulated (Wang *et al.*, 2019). Similarly, BRM directly targets *PIN* genes as can be manifested by lower expression of *PIN1*, *PIN2*, *PIN3*, *PIN4*, and *PIN7* in the *brm* mutant (Yang *et al.*, 2015). However, using ChIP-qPCR, elevated H3K27me3 levels were observed only at *PIN1* and *PIN2* loci in the *brm* mutant (Yang *et al.*, 2015).

Concerning histone acetylation, ELONGATOR COMPLEX SUBUNITs (ELPs) should be mentioned, as *ELP* mutants exhibit several auxin-related phenotypes, and specifically *elp2* and *elp3* also exhibit disrupted auxin biosynthesis, transport, and signalling (Nelissen *et al.*, 2005, 2010). However, mutations in *ELP* genes generate several imbalances in the expression of genes related to phytohormones, including auxin, cytokinins, ethylene, and JAs (Nelissen *et al.*, 2010; Jia *et al.*, 2015). Moreover, the severely disorganized root apical meristem associated with deficiency in *ELP* genes complicates the interpretations (Nelissen *et al.*, 2010; Jia *et al.*, 2015). More specific effects can be found at the levels of H3K14ac at 3' ends of genes, which is related to mRNA elongation and ELP activity *per se*. Mutation in *ELP3* results in lower *LAX2* expression and lower 3'-end acetylation and, similarly, *elp2* exhibits lower *PIN1* levels and lower 3'-end acetylation levels (Nelissen *et al.*, 2010; Jia *et al.*, 2015). Concerning *PIN1* and meristem patterning, another mode of regulation was found by chromatin-remodelling complex INO80 together with NAP1-RELATED PROTEIN 1/2 (NRP1/2). Their triple mutant exhibits higher *PIN1* levels and lower H3 occupancy at the *PIN1* transcribed region, as observed by ChIP-qPCR (Kang *et al.*, 2019).

Although PINs remain the most studied auxin transporters even in terms of epigenetic regulation, there is increasing evidence for regulation of ABCBs and AUX1/LAXs as well. In rice, *OsAUX1* expression is regulated by ROOT LENGTH REGULATOR 4 (OsRLR4), a part of the REGULATOR CHROMOSOME CONDENSATION 1 (RCC1) family (Sun *et al.*, 2022). RCC1 is a nucleosome-binding protein and, in accordance, OsRLR4 is able to bind TRITHORAX-like protein OsTRX1 and target it to the *OsAUX1* promoter, leading to H3K4me3 deposition (England *et al.*, 2010; Sun *et al.*, 2022). This is not the only known epigenetic regulatory mechanism for AUX1/LAXs since EML1 and EML3 seem to affect *LAX2*, *LAX3*, and also *ABCB4* and *ABCB19* expression levels during endosperm development (Milutinovic *et al.*, 2019).

As indicated above, *APOLO* is an important regulator of polar auxin transport thanks to its control over expression of PIN polarity-governing kinase genes *PINOID* (PID) and *WAG2* (Ariel *et al.*, 2014, 2020). *APOLO* can be transcribed by RNA Pol II and V. Upon auxin treatment, *APOLO* expression increases. Interestingly, ChIP-qPCR shows higher RNA Pol II occupancy correlating with higher *APOLO* transcript levels (Ariel *et al.*, 2014). In the native state, the *APOLO* locus is methylated, repressed by LHP1, and brought into proximity to the neighbouring *PID* locus by a formed chromatin loop. Upon auxin induction, *APOLO* is demethylated, and *APOLO* and *PID* transcription rates increase. Subsequently, the chromatin loop is released. As a part of negative feedback, *APOLO* recruits RdDM machinery to repress expression of both genes and formation of the chromatin loop, possibly in a VIM1-dependent manner (Ariel *et al.*, 2014; Fonouni-Farde *et al.*, 2022). Moreover, *APOLO* is not only targeting its own promoter region, but Ariel *et al.* (2020) were able to find more *APOLO* targets using ChIRP-seq. They further confirmed that *APOLO* forms a RNA–DNA hybrid (R-loop) in target regions like *WAG2*, where it acts as a decoy for LHP1 and modulates target gene expression. In conclusion, *APOLO* represents a highly interesting RNA, which shows the complexity of epigenetic regulation in plant hormones by combining RdDM and LHP1-based repression with modulation of chromatin architecture. Considering RdDM regulation of other genes involved in auxin transport, *PIN3*, *PIN4*, and *PIN7* were suggested as potential targets, but the specific mechanism, and indeed the involvement of *APOLO*, is not known (W. Liu *et al.*, 2018; Markulin *et al.*, 2021).

### Exploring the emerging role of epigenetic regulations in auxin signalling

Auxin signalling is initiated by auxin perception via the F-box TRANSPORT INHIBITOR RESPONSE 1/AUXIN SIGNALING F-BOX PROTEIN (TIR1/AFB) auxin co-receptors (Fig. 2; Wang and Estelle, 2014; Lavy and Estelle, 2016; Du *et al.*, 2022). Upon binding of an auxin molecule, TIR1/AFB interacts with transcriptional repressors and auxin co-receptors AUXIN/INDOLE-3-ACETIC ACIDs (AUX/IAAs), triggering a series of downstream events that result in the activation of signalling cascades (Lavy and Estelle, 2016; Yu *et al.*, 2022). The key element of this pathway represents the degradation of AUX/IAAs mediated by SKP, CULLIN, F-BOX CONTAINING COMPLEX (SCF^TIR1/AFB^) E3 ligases, which target AUX/IAAs for ubiquitination and subsequent proteasome-mediated degradation (Salehin *et al.*, 2015). This leads to the activation of transcription factors from the AUXIN RESPONSE FACTOR (ARF) family, which in turn modulate the expression of target genes involved in various developmental processes (Lavy and Estelle, 2016; Yu *et al.*, 2022).

As for auxin metabolism, epigenetic machinery also governs the expression of auxin signalling components. For instance, *AUX/IAA* genes are targeted by Polycomb repression, thereby positively regulating auxin transcriptional responses (Lafos *et al.*, 2011). Moreover, ChIP analysis of H3K14 acetylation of the *IAA3* gene identified lower 3' end histone acetylation in the above-described *elp3* mutant, and its expression is likely to be dependent on GCN5 (Benhamed *et al.*, 2006; Nelissen *et al.*, 2010). Concerning signalling of plant hormones and their epigenetic interactions, TOPLESS/TOPLESS-RELATED (TPL/TPR) should be highlighted (Long *et al.*, 2006; Szemenyei *et al.*, 2008). TPL/TPR act as co-repressors in tandem with HDACs (Plant *et al.*, 2021). Interestingly for auxin signalling, AUX/IAAs are interacting with TPL and thus connecting their activity with HDACs, resulting in histone deacetylation, chromatin condensation, and gene silencing (Szemenyei *et al.*, 2008; Causier *et al.*, 2012). Thus, AUX/IAAs might act as repressors together with histone deacetylation. On the other hand, ARFs were shown to interact with activating epigenetic machineries. ARF7 and ARF19 form a transient complex with ARABIDOPSIS TRITHORAX-RELATED 2 (ATXR2) and JMJ30, as examined *in vivo* by co-immunoprecipitation (co-IP; Fig. 1;) (Lee *et al.*, 2017, 2018). Due to ARF7 and ARF19 DNA binding activities, this complex targets loci encoding transcription factors LATERAL ORGAN BOUNDARIES DOMAINs (LBDs), which are responsible for lateral root initiation and callus formation (Lee *et al.*, 2017, 2018). Mechanistically, JMJ30 presumably decreases H3K9me3 levels on *LDB* genes and ATXR2 increases H3K36me3 levels at their promoters (Lee *et al.*, 2017, 2018). Using bimolecular fluorescence complementation (BiFC), co-IP, and yeast two-hybrid (Y2H) assay, ARFs were observed to regulate SWI/SNF remodellers to open the chromatin architecture, as was shown for MONOPTEROS (MP/ARF5), which interacts with BRM and SPLAYED (SYD), especially during flowering initiation (M.-F. Wu *et al.*, 2015). Further, it is expected that ARFs may play a role in histone acetylation, as reviewed by Nguyen *et al.* (2020). However, thoroughly studied mechanisms involving HAT-recruiting bZIPs were not proven to be directly related to ARF function, although they may activate auxin-related gene expression (Weiste and Dröge-Laser, 2014; Weiste *et al.*, 2017). This system builds an interesting polarity of epigenetic repression by AUX/IAA–TPL–HDACs and gene activation by ARFs. However, the situation of the atypical auxin response factor ETTIN (ETT/ARF3) should be mentioned too. ETT is capable of direct auxin binding and auxin-mediated transcription regulation independently of AUX/IAA repression (Simonini *et al.*, 2017; Kuhn *et al.*, 2020). Since ETT also interacts with TPL, it presumably exhibits both the epigenetically repressive nature of AUX/IAAs and the activating nature of ARFs (Kuhn *et al.*, 2020). This shows that although the ETT function is atypical (compared with other ARFs), the TPL-based repression seems to be a common feature of auxin signalling components.

Apart from histone modifications, small RNAs are important regulators of auxin signalling and certainly a topic for a separate review. Therefore, the authors encourage the readers to read the recent review of Luo *et al.* (2022). In brief, two main mechanisms should be mentioned—miRNAs and *trans*-acting siRNAs (tasiRNAs) which co-regulate mRNA stability of auxin signalling components but also the methylation status of their loci. For instance, *TIR1* genes appear to be targeted by *miRNA393* (Sunkar and Zhu, 2004). The elevation of *miRNA393* under stress conditions implies that stress might trigger higher degradation or reduced translation of *TIR1* mRNA. Given that TIR1 functions as a positive regulator of auxin signalling by inducing the breakdown of AUX/IAA proteins via ubiquitination, the down-regulation of *TIR1* by miRNAs would inhibit plant growth by reducing auxin signalling activity. Further studies showed miRNA-directed regulation of *ARF6*, *ARF8*, *ARF10*, *ARF16*, and *ARF17* by *miRNA167* and *miRNA160*, respectively (Kasschau *et al.*, 2003; Allen *et al.*, 2005; Mallory *et al.*, 2005). The repression of *ARF10* by *miRNA160* was further shown to affect seed germination and post-embryonic developmental programs (P.-P. Liu *et al.*, 2007). However, the absence of histone modification H3K27me3 at *ARF* loci suggests no direct epigenetic regulation via PRC2 activity, but indirect regulation was hinted at by, for instance, H3K27me3-regulated *miRNA393* expression (Lafos *et al.*, 2011). Truskina *et al.* (2021) indicate that such a system would not allow fast changes in signalling output, but it could provide a fine-tuning mechanism in the case of more continual processes such as long-term stress or various developmental processes. They further suggest that rather transcriptional repressors of constitutively active loci expression together with post-translational modifications of ARF proteins provide dynamic auxin responsiveness during development (Cho *et al.*, 2014; Orosa-Puente *et al.*, 2018; Truskina *et al.*, 2021). Based on these findings, the auxin transcriptional responses regulated by epigenetic processes most probably rely on (i) fast regulation by siRNAs and (ii) prolonged molecular regulation via histone modifiers interacting with signalling component and presumably forming a polar duality of repressive AUX/IAAs–TPL–HDACs and activating ARF interactors.

## Cytokinins

Cytokinins are essential plant hormones that regulate important biological processes during plant growth and development (Schaller *et al.*, 2015). Cytokinins have been known for a long time to regulate the cell cycle, meristem activity, and leaf senescence, but also abiotic and biotic stress responses (Kieber and Schaller, 2014, 2018; Skalák *et al.*, 2021).

### Cytokinins and epitranscriptomics—an analogy to epigenetics

**Fig. 3.**
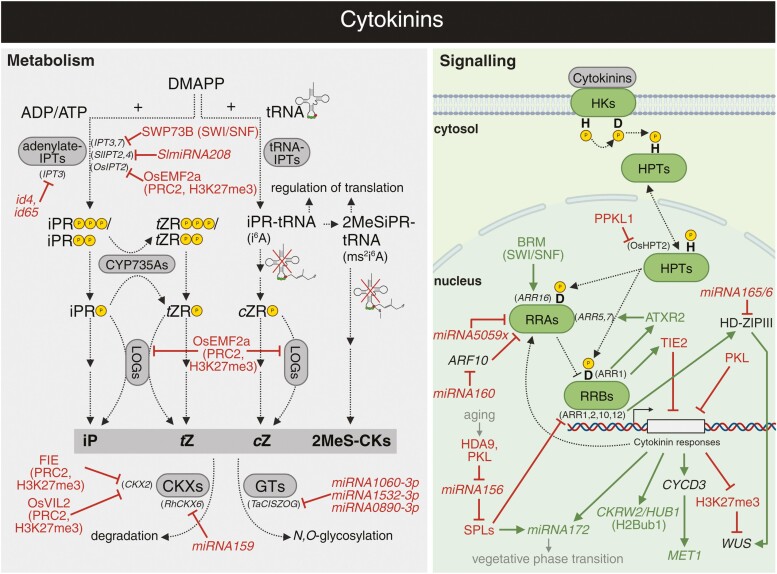
Overview of crosstalk between cytokinins and epigenetic regulation. The interplays between cytokinin metabolism (grey), signalling (green), and epigenetic mechanisms. Arrows in dark green and blunt arrows in red denote the specific positive and negative effects, respectively. See the main text for a detailed description of the illustrated crosstalk. *c*ZR-P, *cis*-zeatin riboside 5ʹ-monophosphate; DMAPP, dimethylallyl pyrophosphate; GT, GLYCOSYLTRANSFERASE; HD-ZIPIII, CLASS III HOMEODOMAIN LEUCINE-ZIPPER; iP, isopentenyladenine; iPR-tRNA, tRNA-bound isopentenyladenosine; iPR-P, isopentenyladenosine 5ʹ-monophosphate; iPR-PP, isopentenyladenosine 5ʹ-diphosphate; iPR-PPP, isopentenyladeninosine 5ʹ-triphosphate; 2MeS-CK, 2-methylthiolated cytokinin; 2MeSiPR-tRNA, tRNA-bound 2-methylthio-isopentenyladenosine; SWI/SNF, SWITCH/SUCROSE NON-FERMENTING; *t*ZR-P, *trans*-zeatin riboside 5ʹ-monophosphate; *t*ZR-PP, *trans*-zeatin riboside 5ʹ-diphosphate; *t*ZR-PPP, *trans*-zeatin riboside 5ʹ-triphosphate. Abbreviations not stated here are explained in the main text. Created with BioRender.com.

The biosynthesis of cytokinins is entwined with RNA and nucleotide metabolism (Miyawaki *et al.*, 2006). In seed plants, two distinct pathways are capable of producing active cytokinins (Fig. 3). The better-described plastid pathway initiates with ADP or ATP nucleotides, which are prenylated by adenylate ISOPENTENYL TRANSFERASES (IPT1, IPT3–IPT8). Subsequently, the resultant derivative can undergo various modifications such as hydroxylation by CYTOCHROME P450 enzymes (CYP735As) or could be directly processed into free active base by LONELY GUYs (LOGs), which acts as a cytokinin riboside 5'-monophosphate phosphoribohydrolase (Takei *et al.*, 2004; Kurakawa *et al.*, 2007; Kuroha *et al.*, 2009; Tokunaga *et al.*, 2012; Wu *et al.*, 2023). The other pathway starts with modification of tRNA by tRNA-IPTs (IPT2 or IPT9), and active cytokinins are released during the degradation of the modified tRNA (Takei *et al.*, 2001; Kasahara *et al.*, 2004; Miyawaki *et al.*, 2004, 2006). Considering zeatin, the most abundant cytokinin representative in plants, these two pathways result in production of its different isomeric forms—*cis*-zeatin (*c*Z) from tRNA and *trans*-zeatin (*t*Z) from ADP/ATP (Kamínek *et al.*, 1979; Sakakibara, 2006).

As cytokinins are mostly isopentenyladenine derivatives, they interfere with isopentenyladenine (i^6^A/iP) function as a non-canonical RNA base, which is present in tRNAs (Senapathy and Jacob, 1981; Jameson, 2023). Moreover, the further modified RNA bases such as prenylated methylthiolated adenines (ms^2^i^6^A) exhibit cytokinin-like activity as well (Gibb *et al.*, 2020). Although such interference makes research in this area more complex, it also hints at a potential interplay between RNA modification and processing, and cytokinin homeostasis and signalling.

Both i^6^A modification and ms^2^i^6^A hypermodification have been identified at anticodon loop position A37 in tRNAs of bacteria and eukaryotes (Schweizer *et al.*, 2017). i^6^A37 is believed to increase the efficiency and accuracy of translation especially with respect to UNN codons (‘N’ represents any nucleotide base; Lamichhane *et al.*, 2013; Aubee *et al.*, 2016; Fleming *et al.*, 2022). The deposition of i^6^A37 is thus a global switch, which affects total outcome of translation in terms of codon usage, mistranslations, and frameshifts. Such global changes are expected to be adaptive mechanisms during bacterial stress responses (Mohler and Ibba, 2017). Similarly, in plants, the codon usage changes during stress as well (Chamani Mohasses *et al.*, 2020; Tyagi *et al.*, 2023). Since production of *c*Z significantly varies in stress conditions (Havlová *et al.*, 2008; Pertry *et al.*, 2009; Vyroubalová *et al.*, 2009), one might speculate whether it is caused by an increased turnover of tRNAs or by differences in the activity of tRNA-modifying IPTs, which in turn could correlate with changes in translation efficiency. While a recent study described the phenotype and hormone profile of *ipt2 ipt9*, our understanding remains limited regarding their impact during stress responses and on the status of tRNA modifications (Silva-Navas *et al.*, 2019; Antoniadi *et al.*, 2022). However, expression profiling suggests their involvement in both biotic and abiotic stress responses (Kilian *et al.*, 2007; Winter *et al.*, 2007).

Proving such an interaction would indeed be methodologically intricate. The oldest methods for study of i^6^A emerged >50 years ago and involved various separation strategies for both tRNAs and digested tRNA nucleotides, such as countercurrent distribution, electrophoresis, affinity chromatography with antibodies, or two-dimensional TLC (Zachau *et al.*, 1966; Goodman *et al.*, 1970; Senapathy and Jacob, 1981). A significant advancement came with the integration of mass spectrometry techniques for precise identification of modifications in combination with high-performance chromatography (Thüring *et al.*, 2016; G. Huang *et al.*, 2023). While this method allowed for the detection and quantification of modifications with lower input of starting material, it presented a limitation: a lack of information about the position of the modifications, since it often works with samples digested to mononucleotides. Alternatively, the sample can be digested only to oligonucleotides and measured in a tandem MS_n_ mode similarly to classical bottom-up proteomics, which provides positional information (Wein *et al.*, 2020). The position of cytokinin modifications can also be obtained by sequencing—a field of newly emerging methods using reverse transcription on zero-mode waveguide chips (Vilfan *et al.*, 2013), direct sequencing by NanoPore (Jain *et al.*, 2022; Ying *et al.*, 2022; Lucas and Novoa, 2023), or methods based on specific further modification and/or cleavage at the position of the modified nucleotide (Motorin and Marchand, 2021). In the case of i^6^A, the recent advancement of click chemistry enabled direct labelling of the base (S. Wang *et al.*, 2022). In the case of ms^2^i^6^A, a method called redox-activated chemical tagging sequencing (ReACT-seq) was recently developed for mapping of this type of modification at single-base resolution. This approach combines specific tagging by a biotinylated moiety, followed by enrichment using affinity chromatography, and then reverse transcription, stopping on the modified base, allowing detection by sequencing.

In conclusion, the epitranscriptomic role of prenylated adenine derivatives cannot be separated from their function as cytokinin signalling molecules. Thus, the role of cytokinins in RNA processing provides another level of regulations independent of changes in the DNA sequence as an analogy to epigenetics.

### The epigenetic control of cytokinin metabolism

Cytokinin metabolism is transcriptionally and post-transcriptionally regulated by epigenetic processes (Fig. 3). The biosynthetic pathways of cytokinins were described above; however for a complete picture, it should be mentioned that the free cytokinin bases can be further reversibly or irreversibly glycosylated (Spíchal *et al.*, 2004; Šmehilová *et al.*, 2016). Moreover, the cytokinins isopentenyladenine, *t*Z, and *c*Z, and their ribosides can be irreversibly cleaved by CYTOKININ OXIDASEs/HYDROGENASEs (CKXs; Paces *et al.*, 1971; Galuszka *et al.*, 2007; Avalbaev *et al.*, 2012; Wang *et al.*, 2020).

Starting with the biosynthetic pathway, the expression of ADP/ATP-dependent *IPT3* and *IPT7* is modulated by SWI/SNF chromatin-remodelling complexes, as was shown for one of its component SWI/SNF ASSOCIATED PROTEIN 73B (SWP73B/CHC1/BAF60; Fig. 3; Jégu *et al.*, 2015). ChIP followed by qPCR showed that SWP73B is capable of binding both promoters and regions downstream of *IPT3* and *IPT7* coding sequences. Chromosome conformation capture (3C) experiments confirmed that SWP73B controls the chromatin conformation at the *IPT3* and *IPT7* loci (Jégu *et al.*, 2015). Collectively, SWP73B acts as a negative regulator of cytokinin biosynthesis. Apart from transcriptional regulation, cytokinin metabolic genes are also targets of small RNAs. For instance, *SlmiRNA208* negatively regulates *SlIPT2* and *SlIPT4* expression, thus reducing cytokinin levels and promoting senescence in tomato plants (Zhang *et al.*, 2020). Moreover, two siRNAs (id*4* and id*65*) were mapped, in the sense and antisense orientations, respectively, to the coding region of the *AtIPT3* orthologue in *Vitis vinifer*a (Carra *et al.*, 2009). In rose, *miRNA159* was shown to target an orthologue of the cytokinin degradation gene *CKX6* in petals. Reducing *miRNA159* levels causes an increase in *RhCKX6* transcripts (Jing *et al.*, 2023).

Seed development serves as one of the main models for studying the interplay between cytokinins and epigenetic mechanisms, owing to its potential applications in crop breeding (Mandal *et al.*, 2022). Moreover, epigenetics plays an important role in the parental conflict in newly developing seeds, during which the maternal and paternal genomes compete over seed trait regulation (Autran *et al.*, 2011; Jameson and Song, 2016). From the evolutionary perspective, the paternal intentions are to maximize resource allocation into seed, which is counterbalanced by maternally inherited genome components, especially PRCs and components of DNA methylation maintenance (Autran *et al.*, 2011; Petrén *et al.*, 2023). PRC2 complexes were shown to deposit H3K27me3 in the *CKX2* promoter region (J. Li *et al.*, 2013). In detail, *CKX2* is ectopically overexpressed in plants with suppressed *FERTILIZATION INDEPENDENT ENDOSPERM* (*FIE*), the core PRC2 subunit (J. Li *et al.*, 2013). Supporting the hypothesis that this regulation is mostly part of the parental conflict, pollination by the paternal genome harbouring a mutation in *DNA METHYLTRANSFERASE 1* (*MET1*) perturbs expression of *CKX2*, thus revealing the importance of genomic imprinting in this process (J. Li *et al.*, 2013). Similarly, excess of the maternal genome resulted in *CKX2* repression (J. Li *et al.*, 2013). This finding is in accordance with the assumption that excess maternal genome promotes expression of genes for PRC2 components (Jullien and Berger, 2010). The role of PRC2 in mediating parental conflict was also observed in rice, where maternally expressed PRC2 complex components have a high impact on cytokinin production. The PHD PRC2 component, VIN3-LIKE 2 (OsVIL2), directly interacts with the *OsCKX2* promoter and participates in the H3K27me3-mediated decrease of *OsCKX2* expression (Wood *et al.*, 2006; De Lucia *et al.*, 2008; Kim and Sung, 2017; Yang *et al.*, 2019). The OsVIL2-mediated reduction of cytokinin degradation results in higher levels of cytokinins, thus improving the production of biomass and grain yield in rice (Yang *et al.*, 2019). Similarly, a mutation in a rice homologue of the Arabidopsis Polycomb group gene *EMBRYONIC FLOWER 2a* (*OsEMF2a*) leads to the hyperaccumulation of cytokinins, correlating with increased expression of *OsIPT2* and *OsLOG1* (Cheng *et al.*, 2021). Methodologically, all the mentioned studies are highly dependent on phenotypic and transcriptomic analyses of mutants and ChIP-qPCR analyses of cytokinin-related genes to decipher the functional importance of binding of selected epigenetic components along with changes in histone modifications. Recent methodological advances have led to the adoption of untargeted methods (e.g. ChIP-seq instead of ChIP-qPCR) and single-cell approaches (e.g. INT-Hi-C), which help to dissect the phenomena with respect to complex organization of developing seeds.

Concerning glycosylation, little is known about its epigenetic regulation. In wheat, *c*Z *O-GLUCOSYLTRANSFERASES* (*TaCISZOG*) can be targeted by several miRNAs, *miRNA1060-3p*, *miRNA1532-3p*, and *miRNA0890-3p* (Cui *et al.*, 2020). Similar regulation can also be expected in the case of *t*Z-modifying enzymes. The overall evidence for epigenetic regulation of cytokinin biosynthesis is not rich, but it is recently emerging due to its potential applications in crop breeding. The most promising topic is clearly cytokinin–epigenetic crosstalk controlling floral and seed development.

### A crosstalk between cytokinin signalling and epigenetics

The cytokinin perception and signal transduction is mediated by multistep phosphorelay (MSP). Mechanistically, the minimal components involve three groups of proteins HISTIDINE KINASEs (HKs), HISTIDINE CONTAINING PHOSPHOTRANSMITTERs (HPTs), and RESPONSE REGULATORs (RRAs, RRBs, and RRCs). Recently, novel negative regulators of MSP, such as TCP INTERACTOR CONTAINING EAR MOTIF PROTEIN 1 (TIE1) and TIE2 and PROTEIN PHOSPHATASE WITH KELCH-LIKE DOMAINS 1 (PPKL1), have been identified (He *et al.*, 2022; Liu *et al.*, 2022). For in-depth details on the cytokinin signalling pathway, we encourage readers to explore recent literature (Heyl *et al.*, 2013; Kieber and Schaller, 2014, 2018; Zürcher and Müller, 2016).

The pivotal components in cytokinin signalling pathways are RRBs. RRBs not only affect the gene expression as transcription factors but also rapidly modulate chromatin accessibility of cytokinin-responsive genes. This has been demonstrated for ARR1, ARR10, and ARR12 by correlating RNA-seq with fluorescence-activated nuclei sorting (FANS)-ATAC-seq in the wild type and mutants (Potter *et al.*, 2018). Similarly, correlating results from RNA-seq, ATAC-seq, and ChIP-seq profiling, Wu *et al.* (2022) found that shoot formation in the non-differentiated tissues (calli) observed at high cytokinin to auxin ratios is associated with changes in the chromatin structure of loci related to callus pluripotency acquisition and shoot fate determination. They also observed specifically altered chromatin in the *arr1 arr10 arr12* triple mutant during callus induction, which they refer to as ‘collapse of pluripotency’. While the involvement of RRBs in this process suggests potential interactions with chromatin remodellers and specific target genes, the full understanding of this mechanism remains to be achieved. Methodologically, these findings, made possible by untargeted approaches, offer a crucial whole-genome perspective on cytokinin-related chromatin changes, even though they do not fully elucidate the underlying mechanisms.

The role of cytokinin-dependent changes in chromatin and corresponding gene expression can be illustrated by the transcription factor WUSCHEL (WUS; Mayer *et al.*, 1998; Kalve *et al.*, 2014; Müller-Xing and Xing, 2022). In brief, *WUS* is a direct target of cytokinin signalling, but it also feed backs to up-regulate cytokinin signalling by repressing RRAs, the negative regulators of MSP (Lopes *et al.*, 2021, and references therein). In terms of epigenetic regulation, cytokinins facilitate the elimination of the suppressive histone mark H3K27me3 at the *WUS* locus during shoot regeneration (T.-Q. Zhang *et al.*, 2017). Following this, RRBs in complex with *miRNA165/6*-targeted HD-ZIP III transcription factors trigger the spatial activation of *WUS* expression (T.-Q. Zhang *et al.*, 2017). Similar effects of cytokinins were observed in auxiliary meristem formation, where cytokinins induce *WUS* expression and require (or even may mediate) epigenetically permissive histone modifications at the *WUS* locus (Wang *et al.*, 2017). Thus, it seems that WUS-regulated shoot apical meristem formation requires a tight cooperation between an epigenetically permissive environment and cytokinin activity. Nonetheless, the underlying mechanism is yet to be fully elucidated (Wang *et al.*, 2017; T.-Q. Zhang *et al.*, 2017).

In general, induction of pluripotency is connected with massive changes in chromatin modification status via histone acetylation and H3K27me3 (Hezroni *et al.*, 2011; Kim *et al.*, 2018; Rymen *et al.*, 2019). Moreover, the maintenance and induction of pluripotency is at least partially cytokinin dependent (Wu *et al.*, 2021; Šmeringai *et al.*, 2023). On the one hand, chemical inhibition of histone acetylation by γ-butyrolactone can block the wound-induced callus (Rymen *et al.*, 2019). On the other hand, the deacetylation inhibitor trichostatin A can partially substitute cytokinin supply during callus growth, which further emphasizes the connection of these two phenomena (Furuta *et al.*, 2011). Mechanistically, this connection is still rather unclear, but two directions can be mentioned—the direct interactor of RRBs, TIE2, and an indirect link between RRs and the chromatin-remodelling complex component PICKLE (PKL; Furuta *et al.*, 2011; He *et al.*, 2022). Since TIE proteins contain an EAR motif, TIEs can be expected to recruit TPL/TPR co-repressors and HDACs (Causier *et al.*, 2012). Therefore, they might connect the deacetylation machinery to the MSP. Another potential link can be represented by PKL, which was originally identified as a *cytokinin hypersensitive* mutant (Kubo and Kakimoto, 2000). PKL is a component of the ATP-dependent CHROMODOMAIN HELICASE-DNA BINDING 3 chromatin-remodelling complex, which is also involved in the above-discussed callogenesis, cell identity, and the vegetative phase change via interaction with HDA9 and subsequent H3K27 deacetylation at *miRNA156* coding loci (Ogas *et al.*, 1999; Henderson *et al.*, 2004; Aichinger *et al.*, 2009; Furuta *et al.*, 2011; Hu *et al.*, 2022).

Cytokinins are also pivotal regulators of the vegetative phase change, a process that is controlled by the *miRNA156*/SQUAMOSA PROMOTER BINDING PROTEIN-LIKE (SPL) module. This module is able to control *de novo* organogenesis via modulation of cytokinin responses possibly by direct interaction of RRBs with SPL transcription factors (Zhang *et al.*, 2015; Barrera-Rojas *et al.*, 2020). Conversely, MSP is hypothesized to co-activate this module as a part of cytokinin-dependent *miRNA172* induction during vegetative phase transition (Werner *et al.*, 2021). A deeper look at the interaction of PKL and cytokinin signalling in the vegetative phase change might explain the cytokinin hypersensitivity phenotype of the *pkl* mutant. Based on the current model, the mutation in *PKL* results in higher expression of *miRNA156*, consequent repression of *SPL* genes, and altered RRB activity by RRB–SPL interactions (Zhang *et al.*, 2015; Barrera-Rojas *et al.*, 2020; Hu *et al.*, 2022).

The only confirmed histone-modifying protein known to interact with RRs is ARABIDOPSIS TRITHORAX-RELATED 2 (ATXR2; Lee *et al.*, 2021). ATXR2 deposits H3K36me3, a mark of transcriptionally active genes and alternative splicing (Pajoro *et al.*, 2017; Lee *et al.*, 2021). Lee *et al.* (2021) showed that the transient ARR1–ATXR2 complex attenuates MSP signalling via up-regulating expression of *RRA* genes by depositing H3K36me3 in the *RRA* promoters, as identified by ChIP-qPCR. The expression of *RRA* genes is also known to be controlled by the SWI/SNF component BRM and several miRNAs (Efroni *et al.*, 2013; Turner *et al.*, 2013; Cui *et al.*, 2020). For instance, *miRNA5059-x* directly affects expression of *RRA* genes in wheat (Cui *et al.*, 2020). Additionally, in soybean, overexpression of *miRNA160* was shown to cause cytokinin hyposensitivity, which is associated with altered expression of *RRA* genes, while suppression of *miRNA160* leads to increased cytokinin sensitivity (Turner *et al.*, 2013; Nizampatnam *et al.*, 2015). This effect may be mechanistically attributed to the suppression of specific *ARF* genes within the auxin signalling pathway. For instance, during callus formation in Arabidopsis, *miRNA160*-mediated repression of *ARF* genes results in the up-regulation of *ARR15* (Liu *et al.*, 2016). Despite these results being partially contradictory, they indicate that miRNA-mediated regulation of the *ARF* level with further impact on cytokinin sensitivity may constitute a common molecular mechanism with phenotypic outcomes that are species dependent.

Last, but not least, it has to be admitted that cytokinins could also regulate the expression of various crucial epigenetic factors, both directly and indirectly. For instance, the methyltransferase *MET1* gene, shown to be a negative regulator of shoot regeneration, is up-regulated by cytokinin-induced expression of *CYCLIN D3* (*CYCD3*; H. Liu *et al.*, 2018). Thus, the cytokinin-induced cell cycle genes might impact DNA methylation status to further regulate shoot initiation (H. Liu *et al.*, 2018). Cytokinins can also induce the expression of *CKRW2/HUB1* (Zhang *et al.*, 2021). As discussed above, CKRW2/HUB1 has a global effect on H2Bub1 deposition, resulting in typical auxin-like phenotypic traits that correlate with diminished expression of auxin biosynthetic genes in the *ckrw2* mutant (Zhang *et al.*, 2021). The crosstalk between cytokinins and auxins, as evidenced by the interplay with CKRW2/HUB1, adds another layer of complexity to the hormonal networks governing plant growth and development.

It is evident that cytokinins significantly influence chromatin architecture and histone modifications. However, apart from the ATXR2-mediated induction of *RRA* genes, the detailed mechanisms underlying these modifications are yet to be fully elucidated. Promising directions involve the long-term ongoing research of PKL function and the ecently described cytokinin interaction with TIEs, which could connect cytokinin signalling with histone deacetylation.

## Ethylene

**Fig. 4.**
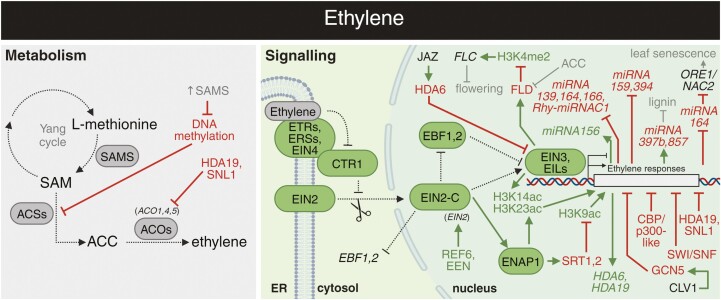
Overview of crosstalk between ethylene and epigenetic regulation. The interplays between ethylene metabolism (grey), signalling (green), and epigenetic mechanisms. Arrows in dark green and blunt arrows in red denote the specific positive and negative effects, respectively. See the main text for a detailed description of the illustrated crosstalk. CLV1, CLAVATA 1 receptor; EBF1, 2, EIN3-BINDING F BOX PROTEIN 1, 2; ER, endoplasmic reticulum; JAZ, JASMONATE ZIM-DOMAIN PROTEIN; ORE1/NAC2, ORESARA 1/NAC DOMAIN CONTAINING PROTEIN 2; SAMS, S-ADENOSYLMETHIONINE SYNTHASE; SWI/SNF, SWITCH/SUCROSE NON-FERMENTING. Abbreviations not stated here are explained in the main text. Created with BioRender.com.

Ethylene is a gaseous plant hormone that regulates a wide range of developmental processes, from apical hook maintenance to flower senescence and fruit ripening (Binder, 2020; Pattyn *et al.*, 2021; Yamoune *et al.*, 2021). There are two steps in the ethylene biosynthetic pathway specifically dedicated to ethylene production. First is the conversion of *S*-adenosylmethionine (SAM) into 5ʹ-methylthioadenosine and 1-aminocyclopropane-1-carboxylic acid (ACC) by ACC SYNTHASEs (ACSs). Second is ACC oxidation by ACC OXIDASEs (ACOs), allowing ethylene production with carbon dioxide and cyanide as byproducts (Fig. 4; Pattyn *et al.*, 2021). Thanks to multiple roles of SAM, ethylene metabolism is tightly linked to epigenetic machinery. For instance, the overexpression of SAM synthase causes globally decreased DNA methylation and increased ethylene content, resulting in abnormal floral organ development (Hu *et al.*, 2023).

Regarding ethylene signalling, the first components include ethylene receptors ETHYLENE RESPONSE 1 (ETR1) and 2 (ETR2), ETHYLENE RESPONSE SENSOR 1 (ERS1) and 2 (ERS2), and ETHYLENE-INSENSITIVE 4 (EIN4; Bleecker *et al.*, 1988). The kinase domain of ethylene receptors physically interacts with CONSTITUTIVE TRIPLE RESPONSE 1 (CTR1) and ETHYLENE-INSENSITIVE 2 (EIN2; Zhong *et al.*, 2008). CTR1, a serine/threonine kinase, functions as a negative regulator of ethylene signalling as it blocks downstream signalling by phosphorylating EIN2 (Kieber *et al.*, 1993). Thus, in the absence of ethylene, EIN2 is inhibited by CTR1, leading to the degradation of canonical downstream components such as EIN3, ETHYLENE-INSENSITIVE-LIKE (EILs), and ETHYLENE RESPONSE FACTORS (ERFs; Bisson and Groth, 2015; Binder, 2020). Ethylene binding inactivates its sensors, leading to the inactivation of CTR1. The hypophosphorylated EIN2 is cleaved, and its C-terminal portion (EIN2-C) moves to the nucleus, further activating ethylene-dependent transcription in cooperation with EIN3 and EIL1 (for a detailed review, see Binder, 2020).

### Ethylene regulation of miRNA function

Using a combination of miRNA-seq, northern blot assays, and RT–qPCR, it was observed that ethylene treatment alters expression of several miRNAs, including *miRNA159*, *miRNA394*, *miRNA139*, *miRNA156*, *miRNA164*, *miRNA166*, and *Rhy-miRNAC1* (Fig. 4; Liu *et al.*, 2009; Pei *et al.*, 2013a, b). For instance, ethylene-modulated expression of several miRNAs (*miRNA156*, *miRNA164*, *miRNA166*, *miRNA5139*, and *Rhy-miRNAC1*) was inversely correlated with the expression of their target genes in rose petals during flower opening (Pei *et al.*, 2013a). Thus, transcriptional control of miRNAs by ethylene provides an interesting mechanism for flower development regulation (Liu and Chen, 2009; Jin *et al.*, 2013). The role of miRNAs in the ethylene-induced leaf senescence pathway has also been elucidated using histochemical analysis of *pmiRNA164:GUS* transgenic plants and ChIP-qPCR assay. In particular, EIN3 was shown to directly bind to the promoter of *miRNA164* and represses its transcription (Z. Li *et al.*, 2013). As a result, expression of *ORESARA1/NAC2*, a target of *miRNA164* and positive regulator of leaf senescence, is progressively elevated during ageing (Z. Li *et al.*, 2013). In contrast, binding of EIN3 and EIL2 to the promoter regions of *miRNA397b* and *miRNA857* activates their transcription. Through this mechanism, ethylene negatively affects lignin biosynthesis, impacting secondary plant growth (Gaddam *et al.*, 2022).

### Chromatin landscape as a key aspect of ethylene signalling activation

Histone deacetylases HDA6 and HDA19 were identified as targets induced by the ethylene signalling pathway, as shown by a stronger *pHDA19:GUS* signal in transgenic plants following ACC treatment, complemented by RT–PCR analysis (Figs 1, 4; Zhou *et al.*, 2005). Further exploration revealed another layer of HDA19–ethylene crosstalk, investigated using a battery of protein–protein interaction techniques, including Y2H assay, BiFC, and immunoprecipitation coupled with LC-MS_n_. HDA19 was shown to interact with SIN3-LIKEs (SNLs), functioning together as components of the HDAC repressor complex, which negatively regulates ethylene biosynthesis (Fig. 4) (Wang *et al.*, 2013; Schmidt and Vélez-Bermúdez, 2023). Mutations in *snl1/2* lead to reduced seed dormancy and increased acetylation levels of H3K9/18 and H3K14, as determined through immunoblotting with specific antibodies (Wang *et al.*, 2013). RNA-seq analysis revealed enhanced expression of genes involved in the ethylene pathway and diminished expression of those involved in ABA signalling, highlighting the importance of SNL1/2 in regulating seed dormancy by modulating ABA–ethylene antagonism (Wang *et al.*, 2013). Concerning HDA6–ethylene interplay, it has been demonstrated that HDA6 is recruited by JAZ proteins to both *EIN3* and *EIL1*, resulting in formation of a repressive complex that inhibits EIN3/EIL1 transcriptional activity (Zhu *et al.*, 2011). JAZ proteins are transcriptional repressors acting in JA signalling. In the presence of JA, these proteins are degraded, which in turn alleviates the JAZ/HDA6-mediated inhibition of ethylene signalling (Zhu *et al.*, 2011).

Another ethylene-regulated epigenetic mechanism involves the SWI/SNF chromatin-remodelling complex that plays a crucial role in nucleosome rearrangement. Sarnowska *et al.* (2013) observed that a knockout mutant deficient in *SWI3C*, a core component of the SWI/SNF complex, exhibited altered responses to hormones such as ethylene, ABA, and GAs. When treated with the ethylene precursor ACC, dark-grown *swi3c* seedlings manifested an ethylene-hypersensitive phenotype characterized by short roots and hypocotyls, suggesting a negative role for SWI/SNF in mediating ethylene responses. In the context of ethylene responses to water availability, Lafon-Placette *et al.* (2018) employed transcriptome profiling and bisulfite sequencing to show that DNA methylation changes play an essential role in modulating expression of ethylene-related genes in poplar plants.

Ethylene-responsive genes are also under the control of activity of HATs controlling histone acetylation levels in the shoot apical meristem. The CLAVATA (CLV) transduction pathway acts synergically with GCN5 inhibiting ethylene response gene expression (Poulios and Vlachonasios, 2016). The *gcn5* mutant exhibited changes in the histone acetylation levels of genes responsive to ethylene, as shown by ChIP-qPCR using antibodies against acetylated H3K9/14 (Poulios and Vlachonasios, 2016). However, the negative role of GCN5 and the exact mechanism of interaction with CLV1 remain mechanistically elusive, relying mainly on genetic proof. The role of GCN5 in this process could be indirect, similarly to the above-discussed GCN5-dependent *YUC4* expression. Poulios and Vlachonasios (2016) also reported altered (both increased and decreased) expression of other HATs in *gcn5*, which could be potentially responsible for observed changes in acetylation patterns.

Additionally, a subfamily of highly conserved HAT genes known as *CBP/p300-like* genes was shown to transcriptionally regulate ethylene-responsive genes (Li *et al.*, 2014). The use of an inhibitor of ethylene synthesis does not alleviate the (ethylene-induced) triple responses observed in mutants of *CBP/p300-like* genes. These findings indicate that CBP/p300-like proteins play a key role in the ethylene signalling pathway (Li *et al.*, 2014). A detailed molecular mechanism regulating transcriptional activation of ethylene-responsive genes via histone acetylation was uncovered recently. Using a combination of ChIP-qPCR and ChIP-seq analysis, ethylene signalling was initially shown to induce H3K14 and H3K23 acetylation levels at downstream target genes in an EIN2-C-dependent manner (Fig. 1) (Zhang *et al.*, 2016). Further investigations employing ChIP-seq analysis revealed that ethylene facilitates the interaction between EIN2-C and EIN2 NUCLEAR ASSOCIATED PROTEIN 1 (ENAP1), a protein that associates with active chromatin regions. Notably, ENAP1 is bound to histones bound to ethylene-responsive genes even in the absence of ethylene, suggesting its role in maintaining an activation-permissive chromatin state. Upon ethylene exposure, the EIN2-C/ENAP1 complex increases histone acetylation and promotes EIN3 binding to its target loci (F. Zhang *et al.*, 2017). Additionally, the binding of EIN3 establishes a positive feedback loop that increases EIN2-C-mediated histone acetylation, further amplifying the expression of genes under ethylene control (L. Wang *et al.*, 2021). ENAP1 also interacts with histone deacetylases SIRTUIN 1 (SRT1) and SRT2 to control transcriptional repression in the ethylene signalling pathway by modulating H3K9 acetylation levels (F. Zhang *et al.*, 2018).

Using the wide range of next-generation sequencing techniques (e.g. ChIP-, RNA-, and bisulfite-seq; Fig. 1), a chromatin-dependent regulatory mechanism was shown in the case of EIN2, requiring H3K27me3 demethylase REF6 (also known as EIN6) and EIN6 ENHANCER (EEN), which is a subunit of the INO80 chromatin-remodelling complex (Zander *et al.*, 2019). In detail, REF6 and EEN cooperatively control the localization and level of the histone variant H2A.Z and the repressive histone modification H3K27me3 at the intronic 5'-untranslated region (UTR) of EIN2. When *EIN6* and *ENN* are simultaneously mutated, there is an ectopic accumulation of H3K27me3 and H2A.Z at EIN2, which creates a repressive chromatin environment leading to a significant reduction in *EIN2* expression (Zander *et al.*, 2019). Recent investigations into the molecular mechanisms linking ethylene and epigenetic control of flowering revealed a direct interaction between the histone demethylase FLD and the ethylene transcription factors EIN3 and EIL1, as shown by Y2H and co-IP assays (Xu *et al.*, 2023). However, exogenous treatment with the ethylene precursor ACC resulted in reduced *FLD* expression, leading to increased enrichment of H3K4me2 at the *FLOWERING LOCUS C* (*FLC*) loci and delayed flowering (Maric, 2023; Xu *et al.*, 2023). These recent studies provide the link between chromatin remodelling and ethylene-mediated control of flowering, a new avenue for potential strategies to enhance crop yield through targeted manipulation of ethylene-related flowering regulation (Campos-Rivero *et al.*, 2017).

## Abscisic acid

The phytohormone ABA is primarily recognized for its crucial role in plant stress responses, particularly under severe environmental conditions such as drought, cold, and salinity. Additionally, ABA also regulates various aspects of seed development as well as several processes at different stages of plant growth (e.g. root stem cell maintenance, bud dormancy, and leaf senescence; for a detailed review, see Chen *et al.*, 2020).

**Fig. 5.**
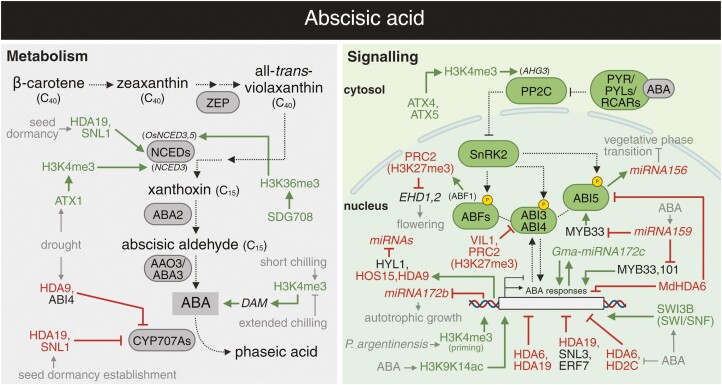
Overview of crosstalk between abscisic acid and epigenetic regulation. The interplays between ABA metabolism (grey), signalling (green), and epigenetic mechanisms. Arrows in dark green and blunt arrows in red denote the specific positive and negative effects, respectively. See the main text for a detailed description of the illustrated crosstalk. ABI3, 4, 5, ABSCISIC ACID INSENSITIVE 3, 4, 5; ABF, ABSCISIC ACID RESPONSIVE ELEMENTS-BINDING FACTOR; AHG3, ABSCISIC ACID-HYPERSENSITIVE GERMINATION 3; ATX1, 4, 5, ARABIDOPSIS TRITHORAX 1, 4, 5; HD2C, HISTONE DEACETYLASE 2C; *P. argentinensis*, *Pseudomonas argentinensis*; ZEP, ZEAXANTHIN EPOXIDASE. Abbreviations not stated here are explained in the main text. Created with BioRender.com.

ABA biosynthesis is initiated in plastids from a carotenoid precursor, leading to production of xanthoxin, which is subsequently oxidized to ABA in the cytosol by the action of ABSCISIC ACID DEFICIENT 2 (ABA2) and ABSCISIC ALDEHYDE OXIDASE 3 (AAO3, also known as ABA3; Fig. 5). If not needed, ABA is catabolized to phaseic acid by CYTOCHROME P450 FAMILY 707 SUBFAMILY A proteins (CYP707As; Sah *et al.*, 2016; Chen *et al.*, 2020). The canonical ABA signalling includes three main components: ABA receptors [PYRABACTIN RESISTANCE 1/PYRABACTIN RESISTANCE-LIKE/REGULATORY COMPONENT OF ABA RECEPTORs (PYR/PYLs/RCARs)], the negative regulator PROTEIN PHOSPHATASE 2C (PP2C), and the positive regulator SNF1-related protein kinase 2 (SnRK2), which regulates downstream target proteins, such as the transcription factor ABA-INSENSITIVE 5 (ABI5), to mediate ABA-related responses (Sah *et al.*, 2016; Chen *et al.*, 2020).

### ABA: the hub of epigenetics and stress responses?

Emerging evidence suggests a direct involvement of histone post-translational modifications in mediating ABA responses. Using ChIP-qPCR, ABA treatment was shown to enhance H3K9K14 acetylation at downstream ABA-inducible genes (Fig. 5; L.-T. Chen *et al.*, 2010). Consistent with these findings, genetic approaches, such as RNAi-mediated down-regulation of *HDA6* or T-DNA mutation in *HDA19* result in an ABA-hypersensitive phenotype, implying that the level of histone acetylation is an important factor in transcriptional regulation of ABA-related genes (Chen and Wu, 2010; L.-T. Chen *et al.*, 2010). HDA19, a negative regulator of histone acetylation, is thought to be recruited to ABA-responsive genes as part of a transcriptional repressor complex that includes ETHYLENE RESPONSE FACTOR 7 and SIN3-LIKE 3 (SNL3; Song *et al.*, 2005; Chen and Wu, 2010). In contrast, interaction of HDA19 with SNL1, another member of the histone deacetylation complex, has been observed through Y2H assay and BiFC, contributing to positive regulation of ABA synthesis and negative modulation of the ethylene pathway during the establishment of seed dormancy (Wang *et al.*, 2013). Interestingly, pull-down assay and BiFC showed that HDA6 can directly bind to another type of HDAC in Arabidopsis, HD2C, forming a repressive complex that negatively regulates ABA responses via histone modifications. Exogenous ABA application can further repress *HD2C* expression, introducing an additional layer of crosstalk regulation (Luo *et al.*, 2012). A recent combination of RNA-seq and H3 acetylation-targeted ChIP-seq revealed that HDA6 (MdHDA6) from apple (*Malus*×*domestica*) mediates histone deacetylation on drought-responsive genes, thus negatively affecting drought tolerance. Counterintuitively, MdHDA6 also forms a complex with the ABA signalling transcription factor MdABI5, leading to the repression of MdABI5-regulated genes (Li *et al.*, 2023). The negative impact of HDACs on plant drought responses is, however, not a universal phenomenon since HDA9, in association with the transcription factor ABI4, was found to negatively regulate expression of ABA catabolic genes and promote drought tolerance (Baek *et al.*, 2020).

Beyond histone acetylation, both histone methylation and chromatin-remodelling complexes (such as the SWI3B component of SWI/SNF) were shown to be crucial for induction of ABA-responsive genes and ABA-dependent pathways (Saez *et al.*, 2008; Ding *et al.*, 2011). Concerning H3K4 trimethylation, the role of ATX1 methyltransferase in promoting ABA production during dehydration stress has been elucidated. The ChIP-qPCR results have demonstrated that water deficit stimulates physical binding of ATX1 to the ABA biosynthetic gene *9-CIS-EPOXYCAROTENOID DIOXYGENASE* (*NCED3*) and increases its occupancy with H3K4me3 and RNA Pol II, leading to *NCED3* transcriptional activation and subsequent ABA accumulation (Ding *et al.*, 2011). Conversely, ChIP-qPCR data indicated that ATX4 and ATX5 regulate the H3K4me3 status at the locus encoding *PP2C* (*AHG3*), a negative regulator of ABA signalling, and thus negatively influencing drought stress responses (Y. Liu *et al.*, 2018). Recent research indicates that the endophytic bacterium *Pseudomonas argentinensis* enhances drought tolerance of plants by priming promoters of drought stress-related genes through the induction of H3K4me3 deposition in an ABA-dependent manner (Alwutayd *et al.*, 2023). In a related context, temperature-mediated changes in gene expression during bud dormancy and break in apple (*Malus*×*domestica*) were elucidated through global epigenome and transcriptome profiling (Chen *et al.*, 2022). Short-term chilling in autumn stimulates H3K4me3 presence over *DORMANCY-ASSOCIATED MADS-box* (*DAM*) genes encoding transcription factors that control ABA levels and facilitate bud dormancy. Extended chilling periods in winter reduce H3K4me3, and decrease *DAM* expression and ABA abundance, aiding dormancy release (Chen *et al.*, 2022). The intricate modulation of H3K4me3 levels by biotic and abiotic factors points to a universal mechanism integrating various external stimuli into ABA metabolism and signalling via epigenetic regulation.

H3K36 methylation, much like H3K4me3, is generally linked with gene activation in plants and its significance in the drought response has also been reported recently. Research combining ChIP-qPCR with physiological analyses of RNAi and overexpressing lines found that the specific H3K36 methyltransferase, SET DOMAIN GROUP 708 (SDG708), enhances drought resistance in rice by directly targeting and activating crucial ABA biosynthesis genes (Chen *et al.*, 2021). However, proper plant development and stress responses require precise and timely fine-tuning of ABA signalling. In line with that, VIN3-LIKE 1 (VIL1) protein was demonstrated to guide PRC2-mediated deposition of the repressive H3K27me3 mark to diminish expression of ABA-responsive transcription factors during early stages of seedling growth. Transcriptional profiling of the *vil1* mutant revealed up-regulated expression of *ABI3* and *ABI4*, leading to ABA hypersensitivity and improved drought tolerance (Zong *et al.*, 2022). By contrast, a recent study utilizing protein–protein and DNA–protein interaction methods, along with ChIP-qPCR, has uncovered the role of ABA signalling in directing PRC2 to flowering regulatory genes in rice. In detail, ABSCISIC ACID RESPONSIVE ELEMENT-BINDING FACTOR 1 (ABF1), a transcription factor induced by the canonical ABA pathway, is responsible for recruiting PRC2 to the promoters of *EPS15 HOMOLOGY DOMAIN* genes (*EHD1* and *EHD2*), positive regulators of flowering. This facilitates the deposition of H3K27me3, which inhibits *EHD1/2* transcription and consequently delays flowering (Tang *et al.*, 2023).

### Dancing in harmony: the mutual orchestration of ABA and miRNA regulation

Similar to other hormones discussed earlier, the activity of ABA is modulated by miRNAs and, conversely, several miRNAs are regulated by ABA treatment. For example, northern blot analysis of germinating seeds exposed to exogenous ABA treatment revealed increased expression of *miRNA159* (Fig. 5) (Reyes and Chua, 2007). Importantly, *miRNA159* further controls the expression of *MYB DOMAIN PROTEIN* transcription factors (MYB101 and MYB33), creating a negative feedback loop to desensitize hormone signalling (Wyrzykowska *et al.*, 2022). Notably, plants overexpressing *MYB101* or *MYB33* genes with a mutated *miRNA159* recognition site exhibit an improved tolerance to drought, highlighting *miRNA159* as a promising candidate for crop improvement (Wyrzykowska *et al.*, 2022). Although a *miRNA159* loss-of-function mutant confers enhanced tolerance to drought, it unfortunately also displays a range of pleiotropic developmental defects (Jiang *et al.*, 2022). Beyond drought response, genetic analyses identified the role of the *miRNA159–MYB33* regulatory module in regulating juvenile to adult transition in an ABI5-dependent manner. Specifically, ChIP-seq analysis confirmed direct binding of MYB33 to the *ABI5* promoter to stimulate its expression. ABI5, in turn, negatively affects vegetative phase change by promoting *miRNA156* expression (Guo *et al.*, 2021). Moreover, the expression of *miRNA159* is also regulated by ethylene and GAs (Liu *et al.*, 2009). Thus, *miRNA159* might serve as a hub for the integration of diverse phytohormonal signals (Liu and Chen, 2009; Liu *et al.*, 2009; Jin *et al.*, 2013).

In Arabidopsis, the activation of the miRNA172 family (*miRNA172b*) is crucial for the post-germination switch from heterotrophic to autotrophic growth. However, when exposed to ABA, plants exhibit suppressed *miRNA172b* expression, ensuring the inhibition of this metabolic transition as a mechanism of stress adaptation (Zou *et al.*, 2013). In the context of stress response, an ABA-inducible *Gma-miRNA172c* from soybean was observed to promote tolerance to drought and salt stress. Transgenic Arabidopsis plants that overexpress *Gma-miRNA172c* become more responsive to ABA, displaying an early flowering phenotype along with up-regulated expression of both ABA signalling and flowering-promoting genes (W. Li *et al.*, 2016). Thus, the functional interaction between the miRNA172 family and ABA signalling might represent a regulatory node for developmental- and stress-related responses.

It is becoming increasingly evident that the biogenesis of miRNAs must be tightly controlled to maintain their homeostatic levels, particularly following their up-regulation or down-regulation in response to various stimuli. Using a forward genetic screen and a subsequent reverse genetics-based CRISPR/CRISPR-associated protein 9 (Cas9) system, Park *et al.* (2023) highlighted the role of HYPONASTIC LEAVES 1 (HYL1) protein, which not only interacts with nascent pri-miRNA transcripts but also attracts the HIGH EXPRESSION OF OSMOTICALLY RESPONSIVE GENES 15 (HOS15)–HDA9 complex to miRNA coding regions, as elucidated by several ChIP assays (Park *et al.*, 2023). As a consequence, this leads to the reduction of histone acetylation, followed by suppressed miRNA expression. Intriguingly, ABA plays a dual role: it acts as a positive regulator of many miRNAs after treatment, as well as an inhibitor of their biogenesis by promoting the formation and association of the HYL1–HOS15–HDA9 complex with target regions, probably fine-tuning miRNA expression (Park *et al.*, 2023).

## Gibberellins

GAs, diterpenoid phytohormones, play pivotal roles in both vegetative and generative plant development. Most importantly for agronomic applications, GAs influence floral and fruit development, seed germination, as well as biomass production (Wu *et al.*, 2020; Castro-Camba *et al.*, 2022). Thus, regulation of GA metabolism or signalling was utilized in the green revolution in the second half of the last century (Castro-Camba *et al.*, 2022). Moreover, GAs represent utilizable elements for further increasing yield and improving nitrogen use efficiency through chromatin modulations to make the green revolution more sustainable (i.e. truly green; Wu *et al.*, 2020; F. Wang *et al.*, 2021).

### Gibberellin metabolism under the control of epigenetics with direct implications for agriculture

**Fig. 6.**
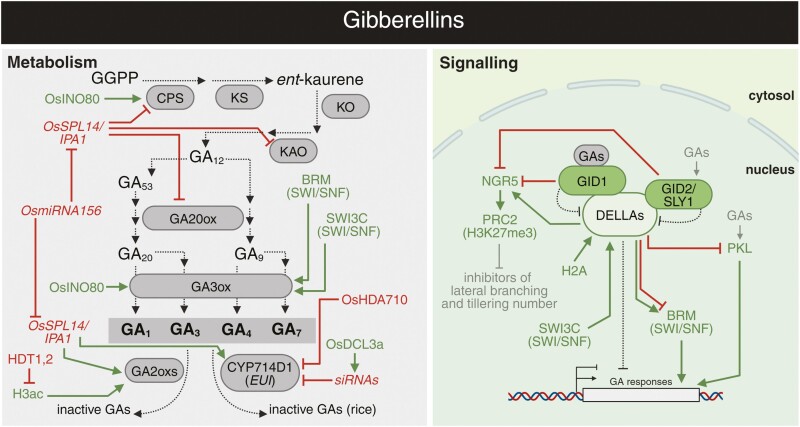
Overview of crosstalk between gibberellins and epigenetic regulation. The interplays between GA metabolism (grey), signalling (green), and epigenetic mechanisms. Arrows in dark green and blunt arrows in red denote the specific positive and negative effects, respectively. See the main text for a detailed description of the illustrated crosstalk. CPS, *ENT*-COPALYL DIPHOSPHATE SYNTHASE; GGPP, geranylgeranyl diphosphate; KS, *ENT*-KAURENE SYNTHASE; SWI/SNF, SWITCH/SUCROSE NON-FERMENTING. Abbreviations not stated here are explained in the main text. Created with BioRender.com.

The biosynthesis of GAs involves three main steps: (i) production of *ent*-kaurene; (ii) its stepwise oxidation to GA12 by ENT-KAURENE OXIDASE (KO) and ENT-KAURENOIC ACID OXIDASE (KAO); and (iii) formation of bioactive GAs by a series of oxidation reactions catalysed by enzymes such as GA20-oxidases (GA20oxs) and GA3-oxidases (GA3oxs; Fig. 6; Sun, 2008).

Application of GAs has a significant impact on complex epigenetic reprogramming, leading to notable chromatin alterations. This includes increased chromatin decondensation, as shown by MNase I digestion assay, and a global decrease in DNA methylation (Manoharlal *et al.*, 2018). On the other hand, chromatin-remodelling complexes play a direct positive role in the GA biosynthetic pathway, namely SWI/SNF components BRM and SWI3C, as well as OsINO80 (Archacki *et al.*, 2013; Sarnowska *et al.*, 2013; Li *et al.*, 2018). Furthermore, an indirect positive regulation of several GA biosynthetic genes has been observed to control seed dormancy in rice through *OsmiRNA156* targeting SQUAMOSA PROMOTER-BINDING-LIKE PROTEIN 14 (OsSPL14) known as IDEAL PLANT ARCHITECTURE 1 (IPA1) transcription factor (Jiao *et al.*, 2010; Miao *et al.*, 2019). Interestingly, disrupting the *OsmiRNA156*-mediated cleavage of *OsSPL14* transcripts, through generation of transgenic plants with point mutations in the *OsmiRNA156* complementary site of *OsSPL14*, results in a rice variety that exhibits altered plant architecture, including reduced tillering and enhanced grain yield, often termed ‘ideal rice’ (Jiao *et al.*, 2010).

Several enzymes orchestrate the catabolism of GAs, with GA2 oxidases (GA2oxs) and CYTOCHROME P450 FAMILY 714 SUBFAMILY D POLYPEPTIDE 1 (CYP714D1), known as ELONGATED UPPERMOST INTERNODE 1 (EUI1), being notably regulated epigenetically by HDACs (Li *et al.*, 2017; Xie *et al.*, 2018). RNA-seq analysis has shown that histone deacetylation mediated by HISTONE DEACETYLASE 2A/2B (HDT1/2) suppresses *GA2ox2* expression. This regulatory mechanism fine-tunes GA metabolism in the root apical meristem, thereby influencing the critical developmental transition from cell division to expansion in Arabidopsis (Li *et al.*, 2017). In rice, *CYP714D1* (*EUI1*) is negatively regulated by HDACs through a distinct mechanism, ensuring GA homeostasis to maintain normal plant growth. In detail, the OsHDA710-containing repressor complex binds to a silencing element within the intronic region of *EUI1* to create a closed chromatin state blocking *EUI1* expression (Xie *et al.*, 2018). Additionally, small RNA-seq and RNA-seq analysis revealed that siRNAs produced by DICER-LIKE 3 homologue (OsDCL3a) target genes involved in GA homeostasis, such as *EUI1*, thus contributing to balanced GA biosynthesis (Wei *et al.*, 2014).

### Gibberellin signalling regulates chromatin architecture

The GA signalling pathway consists of three main components—DELLA proteins (DELLAs; Fig. 6; Rosa *et al.*, 2015; Briones-Moreno *et al.*, 2023), receptor GA-INSENSITIVE DWARF1 (GID1; Ueguchi-Tanaka *et al.*, 2007; Wu *et al.*, 2020; Zhong *et al.*, 2021), and F-box protein GA-INSENSITIVE DWARF2/SLEEPY1 (GID2/SLY1; Gomi *et al.*, 2004). In the absence of GAs, DELLAs inhibit GA-mediated responses. When GAs are present, they associate with the GID1 receptor, which in turn facilitates interaction with DELLA repressors. This promotes their degradation through the proteasomal pathway with the help of the GID2/SLY1-containing SCF complex. As evidenced in rice, the GA-dependent GID1–GID2/SLY1 module not only targets DELLAs but also mediates proteasomal degradation of NITROGEN-MEDIATED TILLER GROWTH RESPONSE 5 (NGR5; Wu *et al.*, 2020). As the concentration of DELLAs rises, the degradation of NGR5 is competitively inhibited. Interestingly, a Y2H screen identified a component of PRC2 as an NGR5-interacting partner. Subsequent genome-wide ChIP-seq analysis revealed that the distribution of the repressive histone mark H3K27me3 is NGR5 dependent. Thus, in response to increased nitrogen supply, NGR5 drives the recruitment of PRC2 to specific target genes, facilitating the spread of the H3K27me3 mark and repressing branching inhibitory genes (Wu *et al.*, 2020). These findings have a promising implication for agriculture, indicating that the manipulation of such a module could improve yield in suboptimal nitrogen conditions.

DELLAs also interact with various components of chromatin-remodelling complexes, including BRM, SWI3C, and PKL, resulting in either a direct or indirect effect on chromatin architecture (Archacki *et al.*, 2013; Sarnowska *et al.*, 2013; Zhang *et al.*, 2014, 2023). The interplay between epigenetic factors and GA signalling components can be illustrated by the chromatin-remodelling protein PKL. Several studies suggested that both PKL and the GA signalling pathway coordinately suppress embryonic traits during seed germination (Henderson *et al.*, 2004; Zhang *et al.*, 2008). PKL plays a positive role in GA signalling, as global RNA-seq data from GA- and/or *PKL*-deficient mutants revealed that 80% of GA-responsive genes depend on PKL functionality (Henderson *et al.*, 2004; Zhang *et al.*, 2008; Park *et al.*, 2017). As mentioned above, DELLAs interfere with PKL by direct protein interaction, which attenuates PKL association with target promoters, a conclusion experimentally supported by pull-down assay (Fig. 1). In line with this observation, GA treatment up-regulates PKL at the protein level and enhances its binding to downstream targets (Zhang *et al.*, 2014).

In addition to chromatin-remodelling factors, DELLAs also directly interact with histone H2A which is essential for DELLA-dependent transcriptional regulation (X. Huang *et al.*, 2023). Given the wide array of interactions, it is plausible that the list of epigenetic factors interacting with DELLAs extends beyond those mentioned. Therefore, DELLAs might be considered as another regulatory hub integrating phytohormonal and epigenetic signalling.

## Exploring the intersection of epigenetics and hormonal regulation

**Fig. 7.**
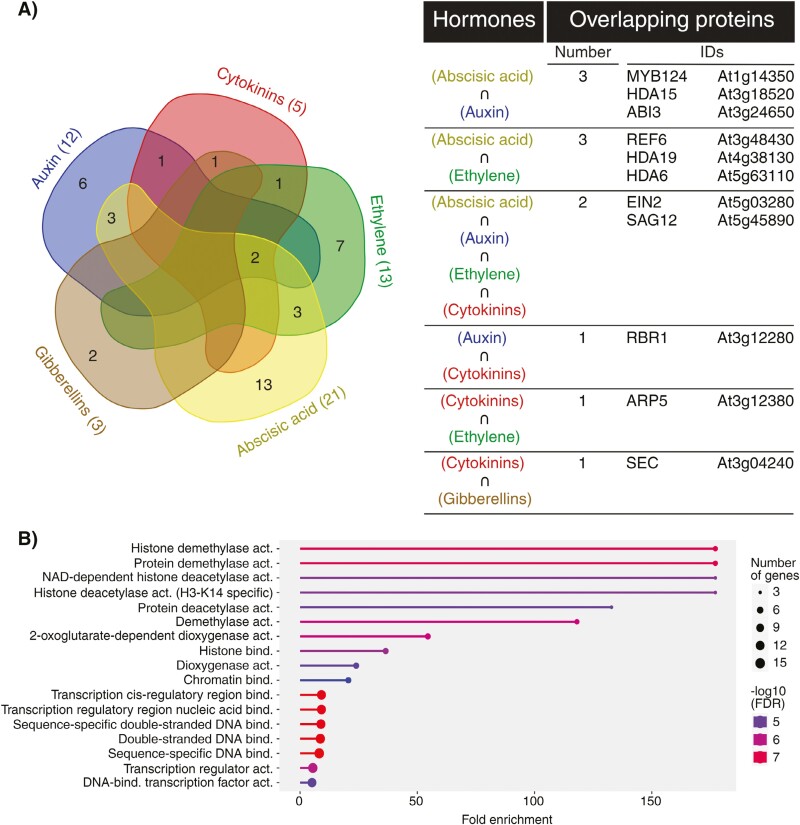
Exploring the intersection of epigenetics and hormonal regulation. (A) Venn diagram created with https://bioinformatics.psb.ugent.be/webtools/Venn/ showing the overlap between genes related to particular plant hormones and epigenetics. (B) Molecular functions of identified proteins from Arabidopsis in term of GO (Gene Ontology) families were analysed using the ShinyGO 0.77 tool (http://bioinformatics.sdstate.edu/go/; Ge *et al.*, 2020) with the default parameters. The figure shows a graphical representation of the GO enrichment analysis of the identified proteins, highlighting the most significant associations (FDR, false discovery rate). Created with BioRender.com.

We conducted a search in the UNIPROT database (UniProt Consortium, 2023) to identify proteins involved in both epigenetics and specific hormonal pathways. This analysis resulted in a list of candidate proteins, revealing previously unrecognized or less-explored protein interactions that contribute to the complex interplay between epigenetics and hormonal regulation. After filtering of proteins to include only ‘reviewed’ proteins, we identified those related to epigenetics simultaneously assigned to auxin (12), cytokinins (5), ethylene (13), ABA (25), or GAs (3), with overlapping categories for ABA–auxin (MYB124, HDA15, ABI3), ABA–ethylene (REF6, HDA19, HDA6), ABA–auxin–ethylene–cytokinins (EIN2, SAG12), auxin–cytokinins (RETINOBLASTOMA-RELATED PROTEIN 1; RBR1), cytokinins–ethylene (ARP5), and cytokinins–GAs (SECRET AGENT; SEC) in Arabidopsis (Fig. 7A; for gene names and their function, see Supplementary Table S1). Keywords ‘auxin’, ‘cytokinins’, ‘ethylene’, ‘abscisic acid’, and ‘gibberellins’ were used as general terms for the UNIPROT search that encompasses various forms and derivatives of a particular plant hormone. Subsequent Gene Ontology (GO) analysis of all Arabidopsis proteins identified in our search uncovered significant enrichment for processes linked to histone demethylation and deacetylation, thus emphasizing the central role of these epigenetic modifications in hormone-mediated regulatory pathways (Fig. 7B).

Interestingly, two proteins, EIN2 and the senescence-specific cysteine protease SENESCENCE ASSOCIATED GENE 12 (SAG12), were identified as concurrently overlapping in epigenetic crosstalk linked to auxin, cytokinins, ethylene, and ABA (Fig. 7A). EIN2, a central component of ethylene signalling, is known to interact with other hormonal pathways. For instance, it forms a complex with ARR2 that drives the expression of the ethylene biosynthetic enzyme ACO4, thereby inducing ethylene levels (Yamoune *et al.*, 2023, Preprint). Moreover, EIN2 was shown to activate auxin signalling, whereas the ABA signalling pathway is altered in the *ein2* mutant line (Ghassemian *et al.*, 2000; Růžička *et al.*, 2007). Given that EIN2 function is tightly regulated by a repressive chromatin environment, multifaceted activity of EIN2 is an excellent example of how an epigenetic regulatory mechanism might co-regulate a plethora of hormonal pathways via a single signalling component (Zander *et al.*, 2019).

The second identified protein, SAG12, is a cysteine protease expressed in senescent tissues, associated with a developmental, senescence-specific cell death function during apoptosis (Noh and Amasino, 1999; Pontier *et al.*, 1999). Its expression is down-regulated by auxin and cytokinins, while being stimulated by ethylene and ABA treatment (Grbić and Bleecker, 1995; Weaver *et al.*, 1998; Noh and Amasino, 1999). SAG12 is also epigenetically regulated by histone H3 deacetylation (Wu *et al.*, 2008). This pleiotropic regulation suggests a complex and finely tuned regulatory mechanism impacting plant ageing. Thus, this raises a major question: what is the primary molecular mechanism in the process of plant ageing? Do plant hormones regulate chromatin status to further control expression of senescence-related genes, or do histone modifications influence hormone signalling pathways and metabolism to regulate plant ageing? The answer is not straightforward, as we show that plant hormones and epigenetic regulators often work in tandem to control various developmental processes in plants. It is more accurate to consider both plant hormones and epigenetic regulations as interconnected components that collectively contribute to the complex regulation of plant development, rather than prioritizing one over the other. As we envisage the future of research, it becomes clear that a comprehensive exploration of both hormonal pathways and epigenetic mechanisms, approached from a global perspective, is essential to unravel the complex molecular networks governing both phenomena.

## Concluding remarks

In summary, our perspective provides an overview of state-of-art techniques and approaches that allowed researchers to elucidate the complex relationship between plant hormones and epigenetic regulation. By unveiling the underlying molecular mechanisms governing hormone–epigenome interactions, this synthesis advances our understanding of plant adaptation, development, and resilience in the face of ever-changing environmental challenges. As new technologies continue to emerge, the synergistic integration of various methodologies promises to unravel even deeper layers of complexity within the captivating nexus of plant hormones and epigenetics. This might answer the fundamental questions such as the following. (i) Crosstalk: what are the extensive crosstalk networks between different plant hormones and their epigenetic effects? How do these interactions impact plant growth and development? (ii) Tissue-specific effects: how do plant hormones and epigenetic modifications operate in a tissue-specific manner? What governs the specificity of their actions in different parts of the plant? (iii) Environmental interactions: how do environmental factors, such as light, temperature, and stress, affect the interplay between plant hormones and epigenetics? What are the implications for plant adaptation and survival? (iv) Long-term memory: how do plants ‘remember’ previous hormonal and epigenetic responses across generations, and what is the significance of this memory for plant fitness and evolution? (v) Epigenetic inheritance (related to the previous question): to what extent are epigenetic changes inheritable in plants, and how do they affect plant responses to hormones and environmental conditions? (vi) Practical applications: how can the knowledge of plant hormone–epigenetic interactions be applied to enhance crop yield, improve stress tolerance, and foster sustainable agriculture practices?

## Supplementary data

The following supplementary data are available at *JXB* online.

Table S1. Proteins identified through UNIPROT database search linking epigenetics and hormonal pathways.

erae054_suppl_Supplementary_Tables_S1

## References

[CIT0001] Aichinger E , Villar CBR, Farrona S, Reyes JC, Hennig L, Köhler C. 2009. CHD3 proteins and polycomb group proteins antagonistically determine cell identity in Arabidopsis. PLoS Genetics 5, e1000605. 19680533 10.1371/journal.pgen.1000605 PMC2718830

[CIT0002] Allen E , Xie Z, Gustafson AM, Carrington JC. 2005. microRNA-directed phasing during trans-acting siRNA biogenesis in plants. Cell 121, 207–221. 15851028 10.1016/j.cell.2005.04.004

[CIT0003] Alwutayd KM , Rawat AA, Sheikh AH, et al. 2023. Microbe-induced drought tolerance by ABA-mediated root architecture and epigenetic reprogramming. EMBO Reports 24, e56754. 37278352 10.15252/embr.202256754 PMC10398642

[CIT0004] Antoniadi I , Mateo-Bonmatí E, Pernisová M, et al. 2022. IPT9, a cis-zeatin cytokinin biosynthesis gene, promotes root growth. Frontiers in Plant Science 13, 932008. 36311087 10.3389/fpls.2022.932008 PMC9616112

[CIT0005] Archacki R , Buszewicz D, Sarnowski TJ, et al. 2013. BRAHMA ATPase of the SWI/SNF chromatin remodeling complex acts as a positive regulator of gibberellin-mediated responses in Arabidopsis. PLoS One 8, e58588. 23536800 10.1371/journal.pone.0058588 PMC3594165

[CIT0006] Ariel F , Jegu T, Latrasse D, Romero-Barrios N, Christ A, Benhamed M, Crespi M. 2014. Noncoding transcription by alternative RNA polymerases dynamically regulates an auxin-driven chromatin loop. Molecular Cell 55, 383–396. 25018019 10.1016/j.molcel.2014.06.011

[CIT0007] Ariel F , Lucero L, Christ A, et al. 2020. R-loop mediated trans action of the APOLO long noncoding RNA. Molecular Cell 77, 1055–1065.e4. 31952990 10.1016/j.molcel.2019.12.015

[CIT0008] Aubee JI , Olu M, Thompson KM. 2016. The i6A37 tRNA modification is essential for proper decoding of UUX-leucine codons during rpoS and iraP translation. RNA 22, 729–742. 26979278 10.1261/rna.053165.115 PMC4836647

[CIT0009] Autran D , Baroux C, Raissig MT, et al. 2011. Maternal epigenetic pathways control parental contributions to Arabidopsis early embryogenesis. Cell 145, 707–719. 21620136 10.1016/j.cell.2011.04.014

[CIT0010] Avalbaev AM , Somov KA, Yuldashev RA, Shakirova FM. 2012. Cytokinin oxidase is key enzyme of cytokinin degradation. Biochemistry 77, 1354–1361. 23244730 10.1134/S0006297912120024

[CIT0011] Baek D , Shin G, Kim MC, Shen M, Lee SY, Yun D-J. 2020. Histone deacetylase HDA9 with ABI4 contributes to abscisic acid homeostasis in drought stress response. Frontiers in Plant Science 11, 143. 32158458 10.3389/fpls.2020.00143 PMC7052305

[CIT0012] Baile F , Gómez-Zambrano A, Calonje M. 2022. Roles of polycomb complexes in regulating gene expression and chromatin structure in plants. Plant Communications 3, 100267. 35059633 10.1016/j.xplc.2021.100267 PMC8760139

[CIT0013] Bajic M , Maher KA, Deal RB. 2018. Identification of open chromatin regions in plant genomes using ATAC-Seq. Methods in Molecular Biology 1675, 183–201. 29052193 10.1007/978-1-4939-7318-7_12 PMC5693289

[CIT0014] Barrera-Rojas CH , Rocha GHB, Polverari L, et al. 2020. miR156-targeted SPL10 controls Arabidopsis root meristem activity and root-derived de novo shoot regeneration via cytokinin responses. Journal of Experimental Botany 71, 934–950. 31642910 10.1093/jxb/erz475

[CIT0015] Benhamed M , Bertrand C, Servet C, Zhou D-X. 2006. Arabidopsis GCN5, HD1, and TAF1/HAF2 interact to regulate histone acetylation required for light-responsive gene expression. The Plant Cell 18, 2893–2903. 17085686 10.1105/tpc.106.043489 PMC1693931

[CIT0016] Binder BM. 2020. Ethylene signaling in plants. Journal of Biological Chemistry 295, 7710–7725. 32332098 10.1074/jbc.REV120.010854 PMC7261785

[CIT0017] Bisson MMA , Groth G. 2015. Targeting plant ethylene responses by controlling essential protein–protein interactions in the ethylene pathway. Molecular Plant 8, 1165–1174. 25843012 10.1016/j.molp.2015.03.014

[CIT0018] Bleecker AB , Estelle MA, Somerville C, Kende H. 1988. Insensitivity to ethylene conferred by a dominant mutation in *Arabidopsis thaliana*. Science 241, 1086–1089. 17747490 10.1126/science.241.4869.1086

[CIT0019] Bourbousse C , Ahmed I, Roudier F, Zabulon G, Blondet E, Balzergue S, Colot V, Bowler C, Barneche F. 2012. Histone H2B monoubiquitination facilitates the rapid modulation of gene expression during Arabidopsis photomorphogenesis. PLoS Genetics 8, e1002825. 22829781 10.1371/journal.pgen.1002825 PMC3400566

[CIT0020] Boycheva I , Vassileva V, Iantcheva A. 2014. Histone acetyltransferases in plant development and plasticity. Current Genomics 15, 28–37. 24653661 10.2174/138920291501140306112742 PMC3958957

[CIT0021] Briones-Moreno A , Hernández-García J, Vargas-Chávez C, Blanco-Touriñán N, Phokas A, Úrbez C, Cerdán PD, Coates JC, Alabadí D, Blázquez MA. 2023. DELLA functions evolved by rewiring of associated transcriptional networks. Nature Plants 9, 535–543. 36914897 10.1038/s41477-023-01372-6

[CIT0022] Campos-Rivero G , Osorio-Montalvo P, Sánchez-Borges R, Us-Camas R, Duarte-Aké F, De-la-Peña C. 2017. Plant hormone signaling in flowering: an epigenetic point of view. Journal of Plant Physiology 214, 16–27. 28419906 10.1016/j.jplph.2017.03.018

[CIT0023] Carra A , Mica E, Gambino G, Pindo M, Moser C, Pè ME, Schubert A. 2009. Cloning and characterization of small non-coding RNAs from grape. The Plant Journal 59, 750–763. 19453456 10.1111/j.1365-313X.2009.03906.x

[CIT0024] Casanova-Sáez R , Mateo-Bonmatí E, Ljung K. 2021. Auxin metabolism in plants. Cold Spring Harbor Perspectives in Biology 13, a039867. 33431579 10.1101/cshperspect.a039867 PMC7919392

[CIT0025] Castro-Camba R , Sánchez C, Vidal N, Vielba JM. 2022. Plant development and crop yield: the role of gibberellins. Plants 11, 2650. 36235516 10.3390/plants11192650 PMC9571322

[CIT0026] Causier B , Ashworth M, Guo W, Davies B. 2012. The TOPLESS interactome: a framework for gene repression in Arabidopsis. Plant Physiology 158, 423–438. 22065421 10.1104/pp.111.186999 PMC3252085

[CIT0027] Chamani Mohasses F , Solouki M, Ghareyazie B, Fahmideh L, Mohsenpour M. 2020. Correlation between gene expression levels under drought stress and synonymous codon usage in rice plant by in-silico study. PLoS One 15, e0237334. 32776991 10.1371/journal.pone.0237334 PMC7416939

[CIT0028] Chen K , Du K, Shi Y, Yin L, Shen W-H, Yu Y, Liu B, Dong A. 2021. H3K36 methyltransferase SDG708 enhances drought tolerance by promoting abscisic acid biosynthesis in rice. New Phytologist 230, 1967–1984. 33606283 10.1111/nph.17290

[CIT0029] Chen K , Li G-J, Bressan RA, Song C-P, Zhu J-K, Zhao Y. 2020. Abscisic acid dynamics, signaling, and functions in plants. Journal of Integrative Plant Biology 62, 25–54. 31850654 10.1111/jipb.12899

[CIT0030] Chen L-T , Luo M, Wang Y-Y, Wu K. 2010. Involvement of Arabidopsis histone deacetylase HDA6 in ABA and salt stress response. Journal of Experimental Botany 61, 3345–3353. 20519338 10.1093/jxb/erq154 PMC2905197

[CIT0031] Chen L-T , Wu K. 2010. Role of histone deacetylases HDA6 and HDA19 in ABA and abiotic stress response. Plant Signaling & Behavior 5, 1318–1320. 20930557 10.4161/psb.5.10.13168 PMC3115378

[CIT0032] Chen Q , Westfall CS, Hicks LM, Wang S, Jez JM. 2010. Kinetic basis for the conjugation of auxin by a GH3 family indole-acetic acid-amido synthetase. Journal of Biological Chemistry 285, 29780–29786. 20639576 10.1074/jbc.M110.146431 PMC2943290

[CIT0033] Chen W , Tamada Y, Yamane H, Matsushita M, Osako Y, Gao-Takai M, Luo Z, Tao R. 2022. H3K4me3 plays a key role in establishing permissive chromatin states during bud dormancy and bud break in apple. The Plant Journal 111, 1015–1031. 35699670 10.1111/tpj.15868

[CIT0034] Chenarani N , Emamjomeh A, Allahverdi A, Mirmostafa S, Afsharinia MH, Zahiri J. 2021. Bioinformatic tools for DNA methylation and histone modification: a survey. Genomics 113, 1098–1113. 33677056 10.1016/j.ygeno.2021.03.004

[CIT0035] Cheng X , Pan M, Zhiguo E, Zhou Y, Niu B, Chen C. 2021. The maternally expressed polycomb group gene OsEMF2a is essential for endosperm cellularization and imprinting in rice. Plant Communications 2, 100092. 33511344 10.1016/j.xplc.2020.100092 PMC7816080

[CIT0036] Cho H , Ryu H, Rho S, et al. 2014. A secreted peptide acts on BIN2-mediated phosphorylation of ARFs to potentiate auxin response during lateral root development. Nature Cell Biology 16, 66–76. 24362628 10.1038/ncb2893

[CIT0037] Cortijo S , Wardenaar R, Colomé-Tatché M, Johannes F, Colot V. 2014. Genome-wide analysis of DNA methylation in Arabidopsis using MeDIP-chip. Methods in Molecular Biology 1112, 125–149. 24478012 10.1007/978-1-62703-773-0_9

[CIT0038] Covington MF , Harmer SL. 2007. The circadian clock regulates auxin signaling and responses in Arabidopsis. PLoS Biology 5, e222. 17683202 10.1371/journal.pbio.0050222 PMC1939880

[CIT0039] Cui G , Zhao M, Zhang S, Wang Z, Meng M, Sun F, Zhang C, Xi Y. 2020. MicroRNA and regulation of auxin and cytokinin signalling during post-mowing regeneration of winter wheat (*Triticum aestivum* L.). Plant Physiology and Biochemistry 155, 769–779. 32866790 10.1016/j.plaphy.2020.08.032

[CIT0040] Cui X , Lu F, Qiu Q, et al. 2016. REF6 recognizes a specific DNA sequence to demethylate H3K27me3 and regulate organ boundary formation in Arabidopsis. Nature Genetics 48, 694–699. 27111035 10.1038/ng.3556

[CIT0041] Cui X , Zheng Y, Lu Y, Issakidis-Bourguet E, Zhou D-X. 2021. Metabolic control of histone demethylase activity involved in plant response to high temperature. Plant Physiology 185, 1813–1828. 33793949 10.1093/plphys/kiab020 PMC8133595

[CIT0042] Cumbie JS , Filichkin SA, Megraw M. 2015. Improved DNase-seq protocol facilitates high resolution mapping of DNase I hypersensitive sites in roots in *Arabidopsis thaliana*. Plant Methods 11, 42. 26339280 10.1186/s13007-015-0087-1 PMC4558764

[CIT0043] Delker C , Quint M, Wigge PA. 2022. Recent advances in understanding thermomorphogenesis signaling. Current Opinion in Plant Biology 68, 102231. 35636376 10.1016/j.pbi.2022.102231

[CIT0044] De Lucia F , Crevillen P, Jones AME, Greb T, Dean C. 2008. A PHD-Polycomb Repressive Complex 2 triggers the epigenetic silencing of FLC during vernalization. Proceedings of the National Academy of Sciences, USA 105, 16831–16836. 10.1073/pnas.0808687105 PMC2579339 18854416

[CIT0045] Derkacheva M , Steinbach Y, Wildhaber T, Mozgová I, Mahrez W, Nanni P, Bischof S, Gruissem W, Hennig L. 2013. Arabidopsis MSI1 connects LHP1 to PRC2 complexes. The EMBO Journal 32, 2073–2085. 23778966 10.1038/emboj.2013.145 PMC3715863

[CIT0046] Ding Y , Avramova Z, Fromm M. 2011. The Arabidopsis trithorax-like factor ATX1 functions in dehydration stress responses via ABA-dependent and ABA-independent pathways. The Plant Journal 66, 735–744. 21309869 10.1111/j.1365-313X.2011.04534.x

[CIT0047] Do BH , Phuong VTB, Tran G-B, Nguyen NH. 2019. Emerging functions of chromatin modifications in auxin biosynthesis in response to environmental alterations. Plant Growth Regulation 87, 165–174.

[CIT0048] Du W , Lu Y, Li Q, Luo S, Shen S, Li N, Chen X. 2022. TIR1/AFB proteins: active players in abiotic and biotic stress signaling. Frontiers in Plant Science 13, 1083409. 36523629 10.3389/fpls.2022.1083409 PMC9745157

[CIT0049] Efroni I , Han S-K, Kim HJ, Wu M-F, Steiner E, Birnbaum KD, Hong JC, Eshed Y, Wagner D. 2013. Regulation of leaf maturation by chromatin-mediated modulation of cytokinin responses. Developmental Cell 24, 438–445. 23449474 10.1016/j.devcel.2013.01.019 PMC3994294

[CIT0050] England JR , Huang J, Jennings MJ, Makde RD, Tan S. 2010. RCC1 uses a conformationally diverse loop region to interact with the nucleosome: a model for the RCC1–nucleosome complex. Journal of Molecular Biology 398, 518–529. 20347844 10.1016/j.jmb.2010.03.037 PMC2895563

[CIT0051] Feng J , Lu J. 2017. LHP1 could act as an activator and a repressor of transcription in plants. Frontiers in Plant Science 8, 2041. 29234344 10.3389/fpls.2017.02041 PMC5712405

[CIT0052] Feng J , Shen W-H. 2014. Dynamic regulation and function of histone monoubiquitination in plants. Frontiers in Plant Science 5, 83. 24659991 10.3389/fpls.2014.00083 PMC3952079

[CIT0053] Feng S , Cokus SJ, Schubert V, Zhai J, Pellegrini M, Jacobsen SE. 2014. Genome-wide Hi-C analyses in wild type and mutants reveal high-resolution chromatin interactions in Arabidopsis. Molecular Cell 55, 694–707. 25132175 10.1016/j.molcel.2014.07.008 PMC4347903

[CIT0054] Figueiredo DD , Batista RA, Roszak PJ, Köhler C. 2015. Auxin production couples endosperm development to fertilization. Nature Plants 1, 15184. 27251719 10.1038/nplants.2015.184

[CIT0055] Fleming BA , Blango MG, Rousek AA, et al. 2022. A tRNA modifying enzyme as a tunable regulatory nexus for bacterial stress responses and virulence. Nucleic Acids Research 50, 7570–7590. 35212379 10.1093/nar/gkac116 PMC9303304

[CIT0056] Foerster AM , Mittelsten Scheid O. 2010. Analysis of DNA methylation in plants by bisulfite sequencing. Methods in Molecular Biology 631, 1–11. 20204863 10.1007/978-1-60761-646-7_1

[CIT0057] Fonouni-Farde C , Christ A, Blein T, et al. 2022. The Arabidopsis APOLO and human UPAT sequence-unrelated long noncoding RNAs can modulate DNA and histone methylation machineries in plants. Genome Biology 23, 181. 36038910 10.1186/s13059-022-02750-7 PMC9422110

[CIT0058] Forgione I , Muto A, Woloszynska M, Chiappetta AA, Ferrari M, Van Lijsebettens M, Bitonti MB, Bruno L. 2022. Epigenetic mechanisms affect the curled leaf phenotype in the hypomethylated *ddc* mutant of *Arabidopsis thaliana*. Plant Science 319, 111254. 35487663 10.1016/j.plantsci.2022.111254

[CIT0059] Forgione I , Wołoszyńska M, Pacenza M, Chiappetta A, Greco M, Araniti F, Abenavoli MR, Van Lijsebettens M, Bitonti MB, Bruno L. 2019. Hypomethylated *drm1 drm2 cmt3* mutant phenotype of *Arabidopsis thaliana* is related to auxin pathway impairment. Plant Science 280, 383–396. 30824017 10.1016/j.plantsci.2018.12.029

[CIT0060] Furuta K , Kubo M, Sano K, Demura T, Fukuda H, Liu Y-G, Shibata D, Kakimoto T. 2011. The CKH2/PKL chromatin remodeling factor negatively regulates cytokinin responses in Arabidopsis calli. Plant and Cell Physiology 52, 618–628. 21357580 10.1093/pcp/pcr022

[CIT0061] Gaddam SR , Bhatia C, Gautam H, Pathak PK, Sharma A, Saxena G, Trivedi PK. 2022. Ethylene regulates miRNA-mediated lignin biosynthesis and leaf serration in *Arabidopsis thaliana*. Biochemical and Biophysical Research Communications 605, 51–55. 35316763 10.1016/j.bbrc.2022.03.037

[CIT0062] Galuszka P , Popelková H, Werner T, Frébortová J, Pospíšilová H, Mik V, Köllmer I, Schmülling T, Frébort I. 2007. Biochemical characterization of cytokinin oxidases/dehydrogenases from *Arabidopsis thaliana* expressed in *Nicotiana tabacum* L. Journal of Plant Growth Regulation 26, 255–267.

[CIT0063] Ge SX , Jung D, Yao R. 2020. ShinyGO: a graphical gene-set enrichment tool for animals and plants. Bioinformatics 36, 2628–2629. 31882993 10.1093/bioinformatics/btz931 PMC7178415

[CIT0064] Geisler MM. 2021. A retro-perspective on auxin transport. Frontiers in Plant Science 12, 756968. 34675956 10.3389/fpls.2021.756968 PMC8524130

[CIT0065] Geisler M , Aryal B, di Donato M, Hao P. 2017. A critical view on ABC transporters and their interacting partners in auxin transport. Plant and Cell Physiology 58, 1601–1614. 29016918 10.1093/pcp/pcx104

[CIT0066] Ghassemian M , Nambara E, Cutler S, Kawaide H, Kamiya Y, McCourt P. 2000. Regulation of abscisic acid signaling by the ethylene response pathway in Arabidopsis. The Plant Cell 12, 1117–1126. 10899978 10.1105/tpc.12.7.1117 PMC149053

[CIT0067] Gibb M , Kisiala AB, Morrison EN, Emery RJN. 2020. The origins and roles of methylthiolated cytokinins: evidence from among life Kingdoms. Frontiers in Cell and Developmental Biology 8, 605672. 33240900 10.3389/fcell.2020.605672 PMC7680852

[CIT0068] Godwin J , Farrona S. 2022. The importance of networking: plant polycomb repressive complex 2 and its interactors. Epigenomes 6, 8. 35323212 10.3390/epigenomes6010008 PMC8948837

[CIT0069] Gomi K , Sasaki A, Itoh H, Ueguchi-Tanaka M, Ashikari M, Kitano H, Matsuoka M. 2004. GID2, an F-box subunit of the SCF E3 complex, specifically interacts with phosphorylated SLR1 protein and regulates the gibberellin-dependent degradation of SLR1 in rice. The Plant Journal 37, 626–634. 14756772 10.1111/j.1365-313x.2003.01990.x

[CIT0070] Goodman HM , Abelson JN, Landy A, Zadrazil S, Smith JD. 1970. The nucleotide sequences of tyrosine transfer RNAs of *Escherichia coli*. European Journal of Biochemistry 13, 461–483. 4315419 10.1111/j.1432-1033.1970.tb00950.x

[CIT0071] Grbić V , Bleecker AB. 1995. Ethylene regulates the timing of leaf senescence in Arabidopsis. The Plant Journal 8, 595–602.

[CIT0072] Gu X , Xu T, He Y. 2014. A histone H3 lysine-27 methyltransferase complex represses lateral root formation in *Arabidopsis thaliana*. Molecular Plant 7, 977–988. 24711289 10.1093/mp/ssu035

[CIT0073] Guo C , Jiang Y, Shi M, Wu X, Wu G. 2021. ABI5 acts downstream of miR159 to delay vegetative phase change in Arabidopsis. New Phytologist 231, 339–350. 33774835 10.1111/nph.17371

[CIT0074] Gyula P , Baksa I, Tóth T, Mohorianu I, Dalmay T, Szittya G. 2018. Ambient temperature regulates the expression of a small set of sRNAs influencing plant development through NF-YA2 and YUC2. Plant, Cell & Environment 41, 2404–2417. 10.1111/pce.13355 29856891

[CIT0075] Han H , Adamowski M, Qi L, Alotaibi SS, Friml J. 2021. PIN-mediated polar auxin transport regulations in plant tropic responses. New Phytologist 232, 510–522. 34254313 10.1111/nph.17617

[CIT0076] Havlová M , Dobrev PI, Motyka V, Štorchová H, Libus J, Dobrá J, Malbeck J, Gaudinová A, Vanková R. 2008. The role of cytokinins in responses to water deficit in tobacco plants over-expressing trans-zeatin O-glucosyltransferase gene under 35S or SAG12 promoters. Plant, Cell & Environment 31, 341–353. 10.1111/j.1365-3040.2007.01766.x 18088334

[CIT0077] He C , Chen X, Huang H, Xu L. 2012. Reprogramming of H3K27me3 is critical for acquisition of pluripotency from cultured arabidopsis tissues. PLoS Genetics 8, e1002911. 22927830 10.1371/journal.pgen.1002911 PMC3426549

[CIT0078] He Q , Yuan R, Zhang T, et al. 2022. Arabidopsis TIE1 and TIE2 transcriptional repressors dampen cytokinin response during root development. Science Advances 8, eabn5057. 36083905 10.1126/sciadv.abn5057 PMC9462699

[CIT0079] Henderson JT , Li H-C, Rider SD, Mordhorst AP, Romero-Severson J, Cheng J-C, Robey J, Sung ZR, de Vries SC, Ogas J. 2004. PICKLE acts throughout the plant to repress expression of embryonic traits and may play a role in gibberellin-dependent responses. Plant Physiology 134, 995–1005. 14963244 10.1104/pp.103.030148 PMC389922

[CIT0080] Heyl A , Brault M, Frugier F, Kuderova A, Lindner A-C, Motyka V, Rashotte AM, Schwartzenberg KV, Vankova R, Schaller GE. 2013. Nomenclature for members of the two-component signaling pathway of plants. Plant Physiology 161, 1063–1065. 23324541 10.1104/pp.112.213207 PMC3585578

[CIT0081] Hezroni H , Tzchori I, Davidi A, Mattout A, Biran A, Nissim-Rafinia M, Westphal H, Meshorer E. 2011. H3K9 histone acetylation predicts pluripotency and reprogramming capacity of ES cells. Nucleus 2, 300–309. 21941115 10.4161/nucl.2.4.16767 PMC3260568

[CIT0082] Himanen K , Woloszynska M, Boccardi TM, De Groeve S, Nelissen H, Bruno L, Vuylsteke M, Van Lijsebettens M. 2012. Histone H2B monoubiquitination is required to reach maximal transcript levels of circadian clock genes in Arabidopsis. The Plant Journal 72, 249–260. 22762858 10.1111/j.1365-313X.2012.05071.x

[CIT0083] Hu T , Manuela D, Hinsch V, Xu M. 2022. PICKLE associates with histone deacetylase 9 to mediate vegetative phase change in Arabidopsis. New Phytologist 235, 1070–1081. 35460275 10.1111/nph.18174 PMC9324081

[CIT0084] Hu W , Hu S, Li S, Zhou Q, Xie Z, Hao X, Wu S, Tian L, Li D. 2023. AtSAMS regulates floral organ development by DNA methylation and ethylene signaling pathway. Plant Science 334, 111767. 37302530 10.1016/j.plantsci.2023.111767

[CIT0085] Huang G , Zhang F, Xie D, Ma Y, Wang P, Cao G, Chen L, Lin S, Zhao Z, Cai Z. 2023. High-throughput profiling of RNA modifications by ultra-performance liquid chromatography coupled to complementary mass spectrometry: methods, quality control, and applications. Talanta 263, 124697. 37262985 10.1016/j.talanta.2023.124697

[CIT0086] Huang X , Tian H, Park J, Oh D-H, Hu J, Zentella R, Qiao H, Dassanayake M, Sun T-P. 2023. The master growth regulator DELLA binding to histone H2A is essential for DELLA-mediated global transcription regulation. Nature Plants 9, 1291–1305. 37537399 10.1038/s41477-023-01477-y PMC10681320

[CIT0087] Jain M , Abu-Shumays R, Olsen HE, Akeson M. 2022. Advances in nanopore direct RNA sequencing. Nature Methods 19, 1160–1164. 36203024 10.1038/s41592-022-01633-w PMC11388133

[CIT0088] Jameson PE. 2023. Zeatin: the 60th anniversary of its identification. Plant Physiology 192, 34–55. 36789623 10.1093/plphys/kiad094 PMC10152681

[CIT0089] Jameson PE , Song J. 2016. Cytokinin: a key driver of seed yield. Journal of Experimental Botany 67, 593–606. 26525061 10.1093/jxb/erv461

[CIT0090] Jégu T , Domenichini S, Blein T, et al. 2015. A SWI/SNF chromatin remodelling protein controls cytokinin production through the regulation of chromatin architecture. PLoS One 10, e0138276. 26457678 10.1371/journal.pone.0138276 PMC4601769

[CIT0091] Jia Y , Tian H, Li H, Yu Q, Wang L, Friml J, Ding Z. 2015. The *Arabidopsis thaliana* elongator complex subunit 2 epigenetically affects root development. Journal of Experimental Botany 66, 4631–4642. 25998905 10.1093/jxb/erv230 PMC4507768

[CIT0092] Jiang Y , Wu X, Shi M, Yu J, Guo C. 2022. The miR159–MYB33–ABI5 module regulates seed germination in Arabidopsis. Physiologia Plantarum 174, e13659. 35244224 10.1111/ppl.13659

[CIT0093] Jiao Y , Wang Y, Xue D, et al. 2010. Regulation of OsSPL14 by OsmiR156 defines ideal plant architecture in rice. Nature Genetics 42, 541–544. 20495565 10.1038/ng.591

[CIT0094] Jin D , Wang Y, Zhao Y, Chen M. 2013. MicroRNAs and their cross-talks in plant development. Journal of Genetics and Genomics 40, 161–170. 23618399 10.1016/j.jgg.2013.02.003

[CIT0095] Jing W , Gong F, Liu G, et al. 2023. Petal size is controlled by the MYB73/TPL/HDA19–miR159–CKX6 module regulating cytokinin catabolism in *Rosa hybrida*. Nature Communications 14, 7106. 10.1038/s41467-023-42914-y PMC10625627 37925502

[CIT0096] Jullien PE , Berger F. 2010. Parental genome dosage imbalance deregulates imprinting in Arabidopsis. PLoS Genetics 6, e1000885. 20333248 10.1371/journal.pgen.1000885 PMC2841625

[CIT0097] Kalve S , De Vos D, Beemster GTS. 2014. Leaf development: a cellular perspective. Frontiers in Plant Science 5, 362. 25132838 10.3389/fpls.2014.00362 PMC4116805

[CIT0098] Kamínek M , Pačes V, Corse J, Challice JS. 1979. Effect of stereospecific hydroxylation of N^6^-(Δ^2^-isopentenyl)adenosine on cytokinin activity. Planta 145, 239–243. 24317729 10.1007/BF00454447

[CIT0099] Kang H , Ma J, Wu D, Shen W-H, Zhu Y. 2019. Functional coordination of the chromatin-remodeling factor AtINO80 and the histone chaperones NRP1/2 in inflorescence meristem and root apical meristem. Frontiers in Plant Science 10, 115. 30792730 10.3389/fpls.2019.00115 PMC6374632

[CIT0100] Kasahara H , Takei K, Ueda N, Hishiyama S, Yamaya T, Kamiya Y, Yamaguchi S, Sakakibara H. 2004. Distinct isoprenoid origins of cis- and trans-zeatin biosyntheses in Arabidopsis. Journal of Biological Chemistry 279, 14049–14054. 14726522 10.1074/jbc.M314195200

[CIT0101] Kasschau KD , Xie Z, Allen E, Llave C, Chapman EJ, Krizan KA, Carrington JC. 2003. P1/HC-Pro, a viral suppressor of RNA silencing, interferes with Arabidopsis development and miRNA function. Developmental Cell 4, 205–217. 12586064 10.1016/s1534-5807(03)00025-x

[CIT0102] Kaufmann K , Muiño JM, Østerås M, Farinelli L, Krajewski P, Angenent GC. 2010. Chromatin immunoprecipitation (ChIP) of plant transcription factors followed by sequencing (ChIP-SEQ) or hybridization to whole genome arrays (ChIP-CHIP). Nature Protocols 5, 457–472. 20203663 10.1038/nprot.2009.244

[CIT0103] Kende H , Zeevaart J. 1997. The five ‘classical’ plant hormones. The Plant Cell 9, 1197–1210. 12237383 10.1105/tpc.9.7.1197 PMC156991

[CIT0104] Kieber JJ , Rothenberg M, Roman G, Feldmann KA, Ecker JR. 1993. CTR1, a negative regulator of the ethylene response pathway in Arabidopsis, encodes a member of the raf family of protein kinases. Cell 72, 427–441. 8431946 10.1016/0092-8674(93)90119-b

[CIT0105] Kieber JJ , Schaller GE. 2014. Cytokinins. The Arabidopsis Book 12, e0168. 24465173 10.1199/tab.0168 PMC3894907

[CIT0106] Kieber JJ , Schaller GE. 2018. Cytokinin signaling in plant development. Development 145, dev149344. 29487105 10.1242/dev.149344

[CIT0107] Kilian J , Whitehead D, Horak J, Wanke D, Weinl S, Batistic O, D’Angelo C, Bornberg-Bauer E, Kudla J, Harter K. 2007. The AtGenExpress global stress expression data set: protocols, evaluation and model data analysis of UV-B light, drought and cold stress responses. The Plant Journal 50, 347–363. 17376166 10.1111/j.1365-313X.2007.03052.x

[CIT0108] Kim D-H , Sung S. 2017. The binding specificity of the PHD-finger domain of VIN3 moderates vernalization response. Plant Physiology 173, 1258–1268. 27999085 10.1104/pp.16.01320 PMC5291027

[CIT0109] Kim J-Y , Yang W, Forner J, Lohmann JU, Noh B, Noh Y-S. 2018. Epigenetic reprogramming by histone acetyltransferase HAG1/AtGCN5 is required for pluripotency acquisition in Arabidopsis. The EMBO Journal 37, e98726. 30061313 10.15252/embj.201798726 PMC6187204

[CIT0110] Kim YJ , Wang R, Gao L, et al. 2016. POWERDRESS and HDA9 interact and promote histone H3 deacetylation at specific genomic sites in Arabidopsis. Proceedings of the National Academy of Sciences, USA 113, 14858–14863. 10.1073/pnas.1618618114 PMC5187680 27930340

[CIT0111] Kubo M , Kakimoto T. 2000. The cytokinin-hypersensitive genes of Arabidopsis negatively regulate the cytokinin-signaling pathway for cell division and chloroplast development. The Plant Journal 23, 385–394. 10929131 10.1046/j.1365-313x.2000.00796.x

[CIT0112] Kuchaříková H , Dobrovolná P, Lochmanová G, Zdráhal Z. 2021. Trimethylacetic anhydride-based derivatization facilitates quantification of histone marks at the ms1 level. Molecular & Cellular Proteomics 20, 100114. 34129942 10.1016/j.mcpro.2021.100114 PMC8283018

[CIT0113] Kuchaříková H , Plšková Z, Zdráhal Z, Fojtová M, Kerchev P, Lochmanová G. 2022. Quantitative analysis of posttranslational modifications of plant histones. Methods in Molecular Biology 2526, 241–257. 35657525 10.1007/978-1-0716-2469-2_18

[CIT0114] Kuhn A , Ramans Harborough S, McLaughlin HM, Natarajan B, Verstraeten I, Friml J, Kepinski S, Østergaard L. 2020. Direct ETTIN–auxin interaction controls chromatin states in gynoecium development. eLife 9, e51787. 32267233 10.7554/eLife.51787 PMC7164952

[CIT0115] Kurakawa T , Ueda N, Maekawa M, Kobayashi K, Kojima M, Nagato Y, Sakakibara H, Kyozuka J. 2007. Direct control of shoot meristem activity by a cytokinin-activating enzyme. Nature 445, 652–655. 17287810 10.1038/nature05504

[CIT0116] Kuroha T , Tokunaga H, Kojima M, Ueda N, Ishida T, Nagawa S, Fukuda H, Sugimoto K, Sakakibara H. 2009. Functional analyses of LONELY GUY cytokinin-activating enzymes reveal the importance of the direct activation pathway in Arabidopsis. The Plant Cell 21, 3152–3169. 19837870 10.1105/tpc.109.068676 PMC2782294

[CIT0117] Lafon-Placette C , Le Gac A-L, Chauveau D, et al. 2018. Changes in the epigenome and transcriptome of the poplar shoot apical meristem in response to water availability affect preferentially hormone pathways. Journal of Experimental Botany 69, 537–551. 29211860 10.1093/jxb/erx409

[CIT0118] Lafos M , Kroll P, Hohenstatt ML, Thorpe FL, Clarenz O, Schubert D. 2011. Dynamic regulation of H3K27 trimethylation during Arabidopsis differentiation. PLoS Genetics 7, e1002040. 21490956 10.1371/journal.pgen.1002040 PMC3072373

[CIT0119] Lamichhane TN , Blewett NH, Crawford AK, Cherkasova VA, Iben JR, Begley TJ, Farabaugh PJ, Maraia RJ. 2013. Lack of tRNA modification isopentenyl-A37 alters mRNA decoding and causes metabolic deficiencies in fission yeast. Molecular and Cellular Biology 33, 2918–2929. 23716598 10.1128/MCB.00278-13 PMC3719670

[CIT0120] Lavy M , Estelle M. 2016. Mechanisms of auxin signaling. Development 143, 3226–3229. 27624827 10.1242/dev.131870 PMC5047657

[CIT0121] Lee H-J , Jung J-H, Cortés Llorca L, Kim S-G, Lee S, Baldwin IT, Park C-M. 2014. FCA mediates thermal adaptation of stem growth by attenuating auxin action in Arabidopsis. Nature Communications 5, 5473. 10.1038/ncomms6473 25400039

[CIT0122] Lee K , Park O-S, Go JY, Yu J, Han JH, Kim J, Bae S, Jung YJ, Seo PJ. 2021. Arabidopsis ATXR2 represses de novo shoot organogenesis in the transition from callus to shoot formation. Cell Reports 37, 109980. 34758306 10.1016/j.celrep.2021.109980

[CIT0123] Lee K , Park O-S, Seo PJ. 2017. Arabidopsis ATXR2 deposits H3K36me3 at the promoters of LBD genes to facilitate cellular dedifferentiation. Science Signaling 10, eaan0316. 29184030 10.1126/scisignal.aan0316

[CIT0124] Lee K , Park O-S, Seo PJ. 2018. JMJ30-mediated demethylation of H3K9me3 drives tissue identity changes to promote callus formation in Arabidopsis. The Plant Journal 95, 961–975. 29923261 10.1111/tpj.14002

[CIT0125] Lee K , Seo PJ. 2017. Coordination of matrix attachment and ATP-dependent chromatin remodeling regulate auxin biosynthesis and Arabidopsis hypocotyl elongation. PLoS One 12, e0181804. 28746399 10.1371/journal.pone.0181804 PMC5529009

[CIT0126] Li C , Chen C, Gao L, et al. 2015. The Arabidopsis SWI2/SNF2 chromatin remodeler BRAHMA regulates polycomb function during vegetative development and directly activates the flowering repressor gene SVP. PLoS Genetics 11, e1004944. 25615622 10.1371/journal.pgen.1004944 PMC4304717

[CIT0127] Li C , Gu L, Gao L, et al. 2016. Concerted genomic targeting of H3K27 demethylase REF6 and chromatin-remodeling ATPase BRM in Arabidopsis. Nature Genetics 48, 687–693. 27111034 10.1038/ng.3555 PMC5134324

[CIT0128] Li C , Liu Y, Shen W-H, Yu Y, Dong A. 2018. Chromatin-remodeling factor OsINO80 is involved in regulation of gibberellin biosynthesis and is crucial for rice plant growth and development. Journal of Integrative Plant Biology 60, 144–159. 29045007 10.1111/jipb.12603

[CIT0129] Li C , Xu J, Li J, Li Q, Yang H. 2014. Involvement of Arabidopsis histone acetyltransferase HAC family genes in the ethylene signaling pathway. Plant and Cell Physiology 55, 426–435. 24287137 10.1093/pcp/pct180

[CIT0130] Li H , Torres-Garcia J, Latrasse D, Benhamed M, Schilderink S, Zhou W, Kulikova O, Hirt H, Bisseling T. 2017. Plant-specific histone deacetylases HDT1/2 regulate GIBBERELLIN 2-OXIDASE2 expression to control Arabidopsis root meristem cell number. The Plant Cell 29, 2183–2196. 28855334 10.1105/tpc.17.00366 PMC5635991

[CIT0131] Li J , Nie X, Tan JLH, Berger F. 2013. Integration of epigenetic and genetic controls of seed size by cytokinin in Arabidopsis. Proceedings of the National Academy of Sciences,USA 110, 15479–15484. 10.1073/pnas.1305175110 PMC3780859 24003120

[CIT0132] Li L , Gallei M, Friml J. 2022. Bending to auxin: fast acid growth for tropisms. Trends in Plant Science 27, 440–449. 34848141 10.1016/j.tplants.2021.11.006

[CIT0133] Li W , Deng M, Wang S, et al. 2023. HISTONE DEACETYLASE 6 interaction with ABSCISIC ACID-INSENSITIVE 5 decreases apple drought tolerance. Plant Physiology 193, kiad468. 10.1093/plphys/kiad468 PMC10663142 37607253

[CIT0134] Li W , Wang T, Zhang Y, Li Y. 2016. Overexpression of soybean miR172c confers tolerance to water deficit and salt stress, but increases ABA sensitivity in transgenic *Arabidopsis thaliana*. Journal of Experimental Botany 67, 175–194. 26466661 10.1093/jxb/erv450

[CIT0135] Li Z , Peng J, Wen X, Guo H. 2013. ETHYLENE-INSENSITIVE3 is a senescence-associated gene that accelerates age-dependent leaf senescence by directly repressing miR164 transcription in Arabidopsis. The Plant Cell 25, 3311–3328. 24064769 10.1105/tpc.113.113340 PMC3809534

[CIT0136] Lin J , Hung F-Y, Ye C, Hong L, Shih Y-H, Wu K, Li QQ. 2020. HDA6-dependent histone deacetylation regulates mRNA polyadenylation in Arabidopsis. Genome Research 30, 1407–1417. 32759225 10.1101/gr.255232.119 PMC7605263

[CIT0137] Liu D , Zhao H, Xiao Y, et al. 2022. A cryptic inhibitor of cytokinin phosphorelay controls rice grain size. Molecular Plant 15, 293–307. 34562665 10.1016/j.molp.2021.09.010

[CIT0138] Liu F , Quesada V, Crevillén P, Bäurle I, Swiezewski S, Dean C. 2007. The Arabidopsis RNA-binding protein FCA requires a lysine-specific demethylase 1 homolog to downregulate FLC. Molecular Cell 28, 398–407. 17996704 10.1016/j.molcel.2007.10.018

[CIT0139] Liu H , Zhang H, Dong YX, Hao YJ, Zhang XS. 2018. DNA METHYLTRANSFERASE1-mediated shoot regeneration is regulated by cytokinin-induced cell cycle in Arabidopsis. New Phytologist 217, 219–232. 28960381 10.1111/nph.14814

[CIT0140] Liu P-P , Montgomery TA, Fahlgren N, Kasschau KD, Nonogaki H, Carrington JC. 2007. Repression of AUXIN RESPONSE FACTOR10 by microRNA160 is critical for seed germination and post-germination stages. The Plant Journal 52, 133–146. 17672844 10.1111/j.1365-313X.2007.03218.x

[CIT0141] Liu Q , Chen Y-Q. 2009. Insights into the mechanism of plant development: interactions of miRNAs pathway with phytohormone response. Biochemical and Biophysical Research Communications 384, 1–5. 19366618 10.1016/j.bbrc.2009.04.028

[CIT0142] Liu Q , Zhang Y-C, Wang C-Y, Luo Y-C, Huang Q-J, Chen S-Y, Zhou H, Qu L-H, Chen Y-Q. 2009. Expression analysis of phytohormone-regulated microRNAs in rice, implying their regulation roles in plant hormone signaling. FEBS Letters 583, 723–728. 19167382 10.1016/j.febslet.2009.01.020

[CIT0143] Liu W , Duttke SH, Hetzel J, et al. 2018. RNA-directed DNA methylation involves co-transcriptional small-RNA-guided slicing of polymerase V transcripts in Arabidopsis. Nature Plants 4, 181–188. 29379150 10.1038/s41477-017-0100-y PMC5832601

[CIT0144] Liu Y , Zhang A, Yin H, Meng Q, Yu X, Huang S, Wang J, Ahmad R, Liu B, Xu Z-Y. 2018. Trithorax-group proteins ARABIDOPSIS TRITHORAX4 (ATX4) and ATX5 function in abscisic acid and dehydration stress responses. New Phytologist 217, 1582–1597. 29250818 10.1111/nph.14933

[CIT0145] Liu Z , Li J, Wang L, Li Q, Lu Q, Yu Y, Li S, Bai M, Hu Y, Xiang F. 2016. Repression of callus initiation by the miRNA-directed interaction of auxin–cytokinin in *Arabidopsis thaliana*. The Plant Journal 87, 391–402. 27189514 10.1111/tpj.13211

[CIT0146] Lochmanová G , Ihnatová I, Kuchaříková H, Brabencová S, Zachová D, Fajkus J, Zdráhal Z, Fojtová M. 2019. Different modes of action of genetic and chemical downregulation of histone deacetylases with respect to plant development and histone modifications. International Journal of Molecular Sciences 20, 5093. 31615119 10.3390/ijms20205093 PMC6829310

[CIT0147] Long JA , Ohno C, Smith ZR, Meyerowitz EM. 2006. TOPLESS regulates apical embryonic fate in Arabidopsis. Science 312, 1520–1523. 16763149 10.1126/science.1123841

[CIT0148] Lopes FL , Galvan-Ampudia C, Landrein B. 2021. WUSCHEL in the shoot apical meristem: old player, new tricks. Journal of Experimental Botany 72, 1527–1535. 33332559 10.1093/jxb/eraa572

[CIT0149] Lucas MC , Novoa EM. 2023. Long-read sequencing in the era of epigenomics and epitranscriptomics. Nature Methods 20, 25–29. 36635539 10.1038/s41592-022-01724-8

[CIT0150] Luo M , Wang Y-Y, Liu X, Yang S, Lu Q, Cui Y, Wu K. 2012. HD2C interacts with HDA6 and is involved in ABA and salt stress response in Arabidopsis. Journal of Experimental Botany 63, 3297–3306. 22368268 10.1093/jxb/ers059 PMC3350937

[CIT0151] Luo P , Di D, Wu L, Yang J, Lu Y, Shi W. 2022. MicroRNAs are involved in regulating plant development and stress response through fine-tuning of TIR1/AFB-dependent auxin signaling. International Journal of Molecular Sciences 23, 510. 35008937 10.3390/ijms23010510 PMC8745101

[CIT0152] Ma S , Tang N, Li X, et al. 2019. Reversible histone H2B monoubiquitination fine-tunes abscisic acid signaling and drought response in rice. Molecular Plant 12, 263–277. 30578854 10.1016/j.molp.2018.12.005

[CIT0153] Macknight R , Bancroft I, Page T, et al. 1997. FCA, a gene controlling flowering time in Arabidopsis, encodes a protein containing RNA-binding domains. Cell 89, 737–745. 9182761 10.1016/s0092-8674(00)80256-1

[CIT0154] Mallory AC , Bartel DP, Bartel B. 2005. MicroRNA-directed regulation of Arabidopsis AUXIN RESPONSE FACTOR17 is essential for proper development and modulates expression of early auxin response genes. The Plant Cell 17, 1360–1375. 15829600 10.1105/tpc.105.031716 PMC1091760

[CIT0155] Mandal S , Ghorai M, Anand U, et al. 2022. Cytokinins: a genetic target for increasing yield potential in the CRISPR era. Frontiers in Genetics 13, 883930. 35559022 10.3389/fgene.2022.883930 PMC9086551

[CIT0156] Manoharlal R , Saiprasad GVS, Ullagaddi C, Kovařík A. 2018. Gibberellin A3 as an epigenetic determinant of global DNA hypo-methylation in tobacco. Biologia Plantarum 62, 11–23.

[CIT0157] March E , Farrona S. 2018. Plant deubiquitinases and their role in the control of gene expression through modification of histones. Frontiers in Plant Science 8, 2274. 29387079 10.3389/fpls.2017.02274 PMC5776116

[CIT0158] Maric A. 2023. Beyond the genetics of flowering: integration of ethylene signaling and histone methylation controls flowering time. Plant Physiology 192, 2224–2226. 37067904 10.1093/plphys/kiad230 PMC10315301

[CIT0159] Markulin L , Škiljaica A, Tokić M, Jagić M, Vuk T, Bauer N, Leljak Levanić D. 2021. Taking the wheel—de novo DNA methylation as a driving force of plant embryonic development. Frontiers in Plant Science 12, 764999. 34777448 10.3389/fpls.2021.764999 PMC8585777

[CIT0160] Mateo-Bonmatí E , Casanova-Sáez R, Ljung K. 2019. Epigenetic regulation of auxin homeostasis. Biomolecules 9, 623. 31635281 10.3390/biom9100623 PMC6843323

[CIT0161] Mayer KF , Schoof H, Haecker A, Lenhard M, Jürgens G, Laux T. 1998. Role of WUSCHEL in regulating stem cell fate in the Arabidopsis shoot meristem. Cell 95, 805–815. 9865698 10.1016/s0092-8674(00)81703-1

[CIT0162] Miao C , Wang Z, Zhang L, Yao J, Hua K, Liu X, Shi H, Zhu J-K. 2019. The grain yield modulator miR156 regulates seed dormancy through the gibberellin pathway in rice. Nature Communications 10, 3822. 10.1038/s41467-019-11830-5 PMC6707268 31444356

[CIT0163] Milutinovic M , Lindsey BE, Wijeratne A, Hernandez JM, Grotewold N, Fernández V, Grotewold E, Brkljacic J. 2019. Arabidopsis EMSY-like (EML) histone readers are necessary for post-fertilization seed development, but prevent fertilization-independent seed formation. Plant Science 285, 99–109. 31203898 10.1016/j.plantsci.2019.04.007

[CIT0164] Miyawaki K , Matsumoto-Kitano M, Kakimoto T. 2004. Expression of cytokinin biosynthetic isopentenyltransferase genes in Arabidopsis: tissue specificity and regulation by auxin, cytokinin, and nitrate. The Plant Journal 37, 128–138. 14675438 10.1046/j.1365-313x.2003.01945.x

[CIT0165] Miyawaki K , Tarkowski P, Matsumoto-Kitano M, Kato T, Sato S, Tarkowska D, Tabata S, Sandberg G, Kakimoto T. 2006. Roles of Arabidopsis ATP/ADP isopentenyltransferases and tRNA isopentenyltransferases in cytokinin biosynthesis. Proceedings of the National Academy of Sciences, USA 103, 16598–16603. 10.1073/pnas.0603522103 PMC1637627 17062755

[CIT0166] Mohler K , Ibba M. 2017. Translational fidelity and mistranslation in the cellular response to stress. Nature Microbiology 2, 1–9. 10.1038/nmicrobiol.2017.117 PMC5697424 28836574

[CIT0167] Motorin Y , Marchand V. 2021. Analysis of RNA modifications by second- and third-generation deep sequencing: 2020 update. Genes 12, 278. 33669207 10.3390/genes12020278 PMC7919787

[CIT0168] Müller-Xing R , Xing Q. 2022. The plant stem-cell niche and pluripotency: 15 years of an epigenetic perspective. Frontiers in Plant Science 13, 1018559. 36388540 10.3389/fpls.2022.1018559 PMC9659954

[CIT0169] Nelissen H , De Groeve S, Fleury D, et al. 2010. Plant Elongator regulates auxin-related genes during RNA polymerase II transcription elongation. Proceedings of the National Academy of Sciences, USA 107, 1678–1683. 10.1073/pnas.0913559107 PMC2824411 20080602

[CIT0170] Nelissen H , Fleury D, Bruno L, Robles P, De Veylder L, Traas J, Micol JL, Van Montagu M, Inzé D, Van Lijsebettens M. 2005. The *elongata* mutants identify a functional Elongator complex in plants with a role in cell proliferation during organ growth. Proceedings of the National Academy of Sciences, USA 102, 7754–7759. 10.1073/pnas.0502600102 PMC1140448 15894610

[CIT0171] Nguyen CT , Tran G-B, Nguyen NH. 2020. Homeostasis of histone acetylation is critical for auxin signaling and root morphogenesis. Plant Molecular Biology 103, 1–7. 32088831 10.1007/s11103-020-00985-1

[CIT0172] Nizampatnam NR , Schreier SJ, Damodaran S, Adhikari S, Subramanian S. 2015. microRNA160 dictates stage-specific auxin and cytokinin sensitivities and directs soybean nodule development. The Plant Journal 84, 140–153. 26287653 10.1111/tpj.12965

[CIT0173] Noh Y-S , Amasino RM. 1999. Identification of a promoter region responsible for the senescence-specific expression of SAG12. Plant Molecular Biology 41, 181–194. 10579486 10.1023/a:1006342412688

[CIT0174] Ogas J , Kaufmann S, Henderson J, Somerville C. 1999. PICKLE is a CHD3 chromatin-remodeling factor that regulates the transition from embryonic to vegetative development in Arabidopsis. Proceedings of the National Academy of Sciences, USA 96, 13839–13844. 10.1073/pnas.96.24.13839 PMC24151 10570159

[CIT0175] Orosa-Puente B , Leftley N, von Wangenheim D, et al. 2018. Root branching toward water involves posttranslational modification of transcription factor ARF7. Science 362, 1407–1410. 30573626 10.1126/science.aau3956

[CIT0176] Paces V , Werstiuk E, Hall RH. 1971. Conversion of N6-(Δ2-isopentenyl)adenosine to adenosine by enzyme activity in tobacco tissue. Plant Physiology 48, 775–778. 16657878 10.1104/pp.48.6.775 PMC396946

[CIT0177] Pajoro A , Muiño JM, Angenent GC, Kaufmann K. 2018. Profiling nucleosome occupancy by MNase-seq: experimental protocol and computational analysis. Methods in Molecular Biology 1675, 167–181. 29052192 10.1007/978-1-4939-7318-7_11

[CIT0178] Pajoro A , Severing E, Angenent GC, Immink RGH. 2017. Histone H3 lysine 36 methylation affects temperature-induced alternative splicing and flowering in plants. Genome Biology 18, 102. 28566089 10.1186/s13059-017-1235-x PMC5452352

[CIT0179] Park J , Giudicatti AJ, Bader ZE, Han MK, Møller C, Arce AL, Xu Z-Y, Yang SW, Manavella PA, Yun D-J. 2023. The HIGH EXPRESSION OF OSMOTICALLY RESPONSIVE GENE15–HISTONE DEACETYLASE9 complex associates with HYPONASTIC LEAVES 1 to modulate microRNA expression in response to abscisic acid signaling. The Plant Cell 35, 2910–2928. 37195876 10.1093/plcell/koad132 PMC10396366

[CIT0180] Park J , Oh D-H, Dassanayake M, Nguyen KT, Ogas J, Choi G, Sun T. 2017. Gibberellin signaling requires chromatin remodeler PICKLE to promote vegetative growth and phase transitions. Plant Physiology 173, 1463–1474. 28057895 10.1104/pp.16.01471 PMC5291033

[CIT0181] Patanun O , Ueda M, Itouga M, et al. 2017. The histone deacetylase inhibitor suberoylanilide hydroxamic acid alleviates salinity stress in Cassava. Frontiers in Plant Science 7, 2039. 28119717 10.3389/fpls.2016.02039 PMC5220070

[CIT0182] Pattyn J , Vaughan-Hirsch J, Van de Poel B. 2021. The regulation of ethylene biosynthesis: a complex multilevel control circuitry. New Phytologist 229, 770–782. 32790878 10.1111/nph.16873 PMC7820975

[CIT0183] Pei H , Ma N, Chen J, Zheng Y, Tian J, Li J, Zhang S, Fei Z, Gao J. 2013a. Integrative analysis of miRNA and mRNA profiles in response to ethylene in rose petals during flower opening. PLoS One 8, e64290. 23696879 10.1371/journal.pone.0064290 PMC3655976

[CIT0184] Pei H , Ma N, Tian J, Luo J, Chen J, Li J, Zheng Y, Chen X, Fei Z, Gao J. 2013b. An NAC transcription factor controls ethylene-regulated cell expansion in flower petals. Plant Physiology 163, 775–791. 23933991 10.1104/pp.113.223388 PMC3793057

[CIT0185] Peng M , Li Z, Zhou N, Ma M, Jiang Y, Dong A, Shen W-H, Li L. 2018. Linking PHYTOCHROME-INTERACTING FACTOR to histone modification in plant shade avoidance. Plant Physiology 176, 1341–1351. 29187567 10.1104/pp.17.01189 PMC5813548

[CIT0186] Pertry I , Václavíková K, Depuydt S, et al. 2009. Identification of *Rhodococcus fascians* cytokinins and their modus operandi to reshape the plant. Proceedings of the National Academy of Sciences, USA 106, 929–934. 10.1073/pnas.0811683106 PMC2630087 19129491

[CIT0187] Petrén H , Thosteman H, Stift M, Toräng P, Ågren J, Friberg M. 2023. Differences in mating system and predicted parental conflict affect post-pollination reproductive isolation in a flowering plant. Evolution 77, 1019–1030. 36734045 10.1093/evolut/qpad016

[CIT0188] Plant AR , Larrieu A, Causier B. 2021. Repressor for hire! The vital roles of TOPLESS-mediated transcriptional repression in plants. New Phytologist 231, 963–973. 33909309 10.1111/nph.17428

[CIT0189] Pontier D , Gan S, Amasino RM, Roby D, Lam E. 1999. Markers for hypersensitive response and senescence show distinct patterns of expression. Plant Molecular Biology 39, 1243–1255. 10380810 10.1023/a:1006133311402

[CIT0190] Potter KC , Wang J, Schaller GE, Kieber JJ. 2018. Cytokinin modulates context-dependent chromatin accessibility through the type-B response regulators. Nature Plants 4, 1102–1111. 30420712 10.1038/s41477-018-0290-y

[CIT0191] Poulios S , Vlachonasios KE. 2016. Synergistic action of histone acetyltransferase GCN5 and receptor CLAVATA1 negatively affects ethylene responses in *Arabidopsis thaliana*. Journal of Experimental Botany 67, 905–918. 26596766 10.1093/jxb/erv503

[CIT0192] Poulios S , Vlachonasios KE. 2018. Synergistic action of GCN5 and CLAVATA1 in the regulation of gynoecium development in *Arabidopsis thaliana*. New Phytologist 220, 593–608. 30027613 10.1111/nph.15303

[CIT0193] Reyes JL , Chua N-H. 2007. ABA induction of miR159 controls transcript levels of two MYB factors during Arabidopsis seed germination. The Plant Journal 49, 592–606. 17217461 10.1111/j.1365-313X.2006.02980.x

[CIT0194] Rizzardi K , Landberg K, Nilsson L, Ljung K, Sundås-Larsson A. 2011. TFL2/LHP1 is involved in auxin biosynthesis through positive regulation of YUCCA genes. The Plant Journal 65, 897–906. 21251106 10.1111/j.1365-313X.2010.04470.x

[CIT0195] Rosa NM , Pfeiffer A, Hill K, et al. 2015. Genome wide binding site analysis reveals transcriptional coactivation of cytokinin-responsive genes by DELLA proteins. PLoS Genetics 11, e1005337. 26134422 10.1371/journal.pgen.1005337 PMC4489807

[CIT0196] Růžička K , Ljung K, Vanneste S, Podhorská R, Beeckman T, Friml J, Benková E. 2007. Ethylene regulates root growth through effects on auxin biosynthesis and transport-dependent auxin distribution. The Plant Cell 19, 2197–2212. 17630274 10.1105/tpc.107.052126 PMC1955700

[CIT0197] Rymen B , Kawamura A, Lambolez A, et al. 2019. Histone acetylation orchestrates wound-induced transcriptional activation and cellular reprogramming in Arabidopsis. Communications Biology 2, 404. 31701032 10.1038/s42003-019-0646-5 PMC6828771

[CIT0198] Saez A , Rodrigues A, Santiago J, Rubio S, Rodriguez PL. 2008. HAB1–SWI3B interaction reveals a link between abscisic acid signaling and putative SWI/SNF chromatin-remodeling complexes in Arabidopsis. The Plant Cell 20, 2972–2988. 19033529 10.1105/tpc.107.056705 PMC2613670

[CIT0199] Sah SK , Reddy KR, Li J. 2016. Abscisic acid and abiotic stress tolerance in crop plants. Frontiers in Plant Science 7, 571. 27200044 10.3389/fpls.2016.00571 PMC4855980

[CIT0200] Sakakibara H. 2006. CYTOKININS: activity, biosynthesis, and translocation. Annual Review of Plant Biology 57, 431–449. 10.1146/annurev.arplant.57.032905.105231 16669769

[CIT0201] Salehin M , Bagchi R, Estelle M. 2015. SCFTIR1/AFB-based auxin perception: mechanism and role in plant growth and development. The Plant Cell 27, 9–19. 25604443 10.1105/tpc.114.133744 PMC4330579

[CIT0202] Sarnowska EA , Rolicka AT, Bucior E, et al. 2013. DELLA-interacting SWI3C core subunit of switch/sucrose nonfermenting chromatin remodeling complex modulates gibberellin responses and hormonal cross talk in Arabidopsis. Plant Physiology 163, 305–317. 23893173 10.1104/pp.113.223933 PMC3762652

[CIT0203] Schaller GE , Bishopp A, Kieber JJ. 2015. The Yin–Yang of hormones: cytokinin and auxin interactions in plant development. The Plant Cell 27, 44–63. 25604447 10.1105/tpc.114.133595 PMC4330578

[CIT0204] Schmidt W , Vélez-Bermúdez I. 2023. The interactome of histone deacetylase HDA19 in dark-grown Arabidopsis seedlings. Frontiers in Plant Science 14, 1296767. 38078106 10.3389/fpls.2023.1296767 PMC10701890

[CIT0205] Schweizer U , Bohleber S, Fradejas-Villar N. 2017. The modified base isopentenyladenosine and its derivatives in tRNA. RNA Biology 14, 1197–1208. 28277934 10.1080/15476286.2017.1294309 PMC5699536

[CIT0206] Senapathy P , Jacob MT. 1981. Identification and purification of tRNAs containing *N*^6^-(Δ^2^-isopentenyl) adenosine using antibodies specific for *N*^6^-(Δ^2^-isopentenyl) adenosine. Journal of Biological Chemistry 256, 11580–11584. 7028736

[CIT0207] Silva-Navas J , Conesa CM, Saez A, Navarro-Neila S, Garcia-Mina JM, Zamarreño AM, Baigorri R, Swarup R, del Pozo JC. 2019. Role of cis-zeatin in root responses to phosphate starvation. New Phytologist 224, 242–257. 31230346 10.1111/nph.16020

[CIT0208] Simonini S , Bencivenga S, Trick M, Østergaard L. 2017. Auxin-induced modulation of ETTIN activity orchestrates gene expression in Arabidopsis. The Plant Cell 29, 1864–1882. 28804059 10.1105/tpc.17.00389 PMC5590509

[CIT0209] Skalák J , Nicolas KL, Vankova R, Hejatko J. 2021. Signal integration in plant abiotic stress responses via multistep phosphorelay signaling. Frontiers in Plant Science 12, 644823. 33679861 10.3389/fpls.2021.644823 PMC7925916

[CIT0210] Šmehilová M , Dobrůšková J, Novák O, Takáč T, Galuszka P. 2016. Cytokinin-specific glycosyltransferases possess different roles in cytokinin homeostasis maintenance. Frontiers in Plant Science 7, 1264. 27602043 10.3389/fpls.2016.01264 PMC4993776

[CIT0211] Šmeringai J , Schrumpfová PP, Pernisová M. 2023. Cytokinins—regulators of de novo shoot organogenesis. Frontiers in Plant Science 14, 1239133. 37662179 10.3389/fpls.2023.1239133 PMC10471832

[CIT0212] Solanki M , Shukla LI. 2023. Recent advances in auxin biosynthesis and homeostasis. 3 Biotech 13, 290. 10.1007/s13205-023-03709-6 PMC10400529 37547917

[CIT0213] Song C-P , Agarwal M, Ohta M, Guo Y, Halfter U, Wang P, Zhu J-K. 2005. Role of an Arabidopsis AP2/EREBP-type transcriptional repressor in abscisic acid and drought stress responses. The Plant Cell 17, 2384–2396. 15994908 10.1105/tpc.105.033043 PMC1182496

[CIT0214] Sonmez C , Bäurle I, Magusin A, Dreos R, Laubinger S, Weigel D, Dean C. 2011. RNA 3ʹ processing functions of Arabidopsis FCA and FPA limit intergenic transcription. Proceedings of the National Academy of Sciences, USA 108, 8508–8513. 10.1073/pnas.1105334108 PMC3100917 21536901

[CIT0215] Spíchal L , Rakova NY, Riefler M, Mizuno T, Romanov GA, Strnad M, Schmülling T. 2004. Two cytokinin receptors of *Arabidopsis thaliana*, CRE1/AHK4 and AHK3, differ in their ligand specificity in a bacterial assay. Plant & Cell Physiology 45, 1299–1305. 15509853 10.1093/pcp/pch132

[CIT0216] Sun C , Li D, Gao Z, et al. 2022. OsRLR4 binds to the OsAUX1 promoter to negatively regulate primary root development in rice. Journal of Integrative Plant Biology 64, 118–134. 34726825 10.1111/jipb.13183

[CIT0217] Sun T. 2008. Gibberellin metabolism, perception and signaling pathways in Arabidopsis. The Arabidopsis Book 6, e0103. 22303234 10.1199/tab.0103 PMC3243332

[CIT0218] Sunkar R , Zhu J-K. 2004. Novel and stress-regulated microRNAs and other small RNAs from Arabidopsis. The Plant Cell 16, 2001–2019. 15258262 10.1105/tpc.104.022830 PMC519194

[CIT0219] Swarup R , Bhosale R. 2019. Developmental roles of AUX1/LAX auxin influx carriers in plants. Frontiers in Plant Science 10, 1306. 31719828 10.3389/fpls.2019.01306 PMC6827439

[CIT0220] Szemenyei H , Hannon M, Long JA. 2008. TOPLESS mediates auxin-dependent transcriptional repression during arabidopsis embryogenesis. Science 319, 1384–1386. 18258861 10.1126/science.1151461

[CIT0221] Takei K , Sakakibara H, Sugiyama T. 2001. Identification of genes encoding adenylate isopentenyltransferase, a cytokinin biosynthesis enzyme, in *Arabidopsis thaliana*. Journal of Biological Chemistry 276, 26405–26410. 11313355 10.1074/jbc.M102130200

[CIT0222] Takei K , Yamaya T, Sakakibara H. 2004. Arabidopsis CYP735A1 and CYP735A2 encode cytokinin hydroxylases that catalyze the biosynthesis of trans-zeatin. Journal of Biological Chemistry 279, 41866–41872. 15280363 10.1074/jbc.M406337200

[CIT0223] Tang L , Li G, Wang H, et al. 2023. Exogenous abscisic acid represses rice flowering via SAPK8–ABF1–Ehd1/Ehd2 pathway. Journal of Advanced Research doi:10.1016/j.jare.2023.06.012 PMC11081964 37399924

[CIT0224] Tasset C , Singh Yadav A, Sureshkumar S, Singh R, Van Der Woude L, Nekrasov M, Tremethick D, Van Zanten M, Balasubramanian S. 2018. POWERDRESS-mediated histone deacetylation is essential for thermomorphogenesis in *Arabidopsis thaliana*. PLoS Genetics 14, e1007280. 29547672 10.1371/journal.pgen.1007280 PMC5874081

[CIT0225] Thüring K , Schmid K, Keller P, Helm M. 2016. Analysis of RNA modifications by liquid chromatography–tandem mass spectrometry. Methods 107, 48–56. 27020891 10.1016/j.ymeth.2016.03.019

[CIT0226] Tian Y , Zheng H, Zhang F, Wang S, Ji X, Xu C, He Y, Ding Y. 2019. PRC2 recruitment and H3K27me3 deposition at FLC require FCA binding of COOLAIR. Science Advances 5, eaau7246. 31032401 10.1126/sciadv.aau7246 PMC6482009

[CIT0227] Tokunaga H , Kojima M, Kuroha T, Ishida T, Sugimoto K, Kiba T, Sakakibara H. 2012. Arabidopsis lonely guy (LOG) multiple mutants reveal a central role of the LOG-dependent pathway in cytokinin activation. The Plant Journal 69, 355–365. 22059596 10.1111/j.1365-313X.2011.04795.x

[CIT0228] Truskina J , Han J, Chrysanthou E, et al. 2021. A network of transcriptional repressors modulates auxin responses. Nature 589, 116–119. 33208947 10.1038/s41586-020-2940-2

[CIT0229] Turner M , Nizampatnam NR, Baron M, Coppin S, Damodaran S, Adhikari S, Arunachalam SP, Yu O, Subramanian S. 2013. Ectopic expression of miR160 results in auxin hypersensitivity, cytokinin hyposensitivity, and inhibition of symbiotic nodule development in soybean. Plant Physiology 162, 2042–2055. 23796794 10.1104/pp.113.220699 PMC3729781

[CIT0230] Tyagi P , Singh D, Mathur S, Singh A, Ranjan R. 2022. Upcoming progress of transcriptomics studies on plants: an overview. Frontiers in Plant Science 13, 1030890. 36589087 10.3389/fpls.2022.1030890 PMC9798009

[CIT0231] Tyagi S , Kabade PG, Gnanapragasam N, Singh UM, Gurjar AKS, Rai A, Sinha P, Kumar A, Singh VK. 2023. Codon usage provide insights into the adaptation of rice genes under stress condition. International Journal of Molecular Sciences 24, 1098. 36674611 10.3390/ijms24021098 PMC9861248

[CIT0232] Ueguchi-Tanaka M , Nakajima M, Katoh E, et al. 2007. Molecular interactions of a soluble gibberellin receptor, GID1, with a rice DELLA protein, SLR1, and gibberellin. The Plant Cell 19, 2140–2155. 17644730 10.1105/tpc.106.043729 PMC1955699

[CIT0233] Ung KL , Schulz L, Kleine-Vehn J, Pedersen BP, Hammes UZ. 2023. Auxin transport at the endoplasmic reticulum: roles and structural similarity of PIN-FORMED and PIN-LIKES. Journal of Experimental Botany 74, erad192. 10.1093/jxb/erad192 37279330

[CIT0234] UniProt Consortium. 2023. UniProt: the universal protein knowledgebase in 2023. Nucleic Acids Research 51, D523–D531. 36408920 10.1093/nar/gkac1052 PMC9825514

[CIT0235] van der Woude LC , Perrella G, Snoek BL, et al. 2019. HISTONE DEACETYLASE 9 stimulates auxin-dependent thermomorphogenesis in *Arabidopsis thaliana* by mediating H2A.Z depletion. Proceedings of the National Academy of Sciences, USA 116, 25343–25354. 10.1073/pnas.1911694116 PMC6911240 31767749

[CIT0236] Verma S , Attuluri VPS, Robert HS. 2021. An essential function for auxin in embryo development. Cold Spring Harbor Perspectives in Biology 13, a039966. 33431580 10.1101/cshperspect.a039966 PMC8015691

[CIT0237] Vilfan ID , Tsai Y-C, Clark TA, Wegener J, Dai Q, Yi C, Pan T, Turner SW, Korlach J. 2013. Analysis of RNA base modification and structural rearrangement by single-molecule real-time detection of reverse transcription. Journal of Nanobiotechnology 11, 8. 23552456 10.1186/1477-3155-11-8 PMC3623877

[CIT0238] Voß U , Wilson MH, Kenobi K, et al. 2015. The circadian clock rephases during lateral root organ initiation in *Arabidopsis thaliana*. Nature Communications 6, 7641. 10.1038/ncomms8641 PMC4506504 26144255

[CIT0239] Vyroubalová S , Václavíková K, Turečková V, Novák O, Šmehilová M, Hluska T, Ohnoutková L, Frébort I, Galuszka P. 2009. Characterization of new maize genes putatively involved in cytokinin metabolism and their expression during osmotic stress in relation to cytokinin levels. Plant Physiology 151, 433–447. 19641027 10.1104/pp.109.142489 PMC2735981

[CIT0240] Wang F , Yoshida H, Matsuoka M. 2021. Making the ‘Green Revolution’ truly green: improving crop nitrogen use efficiency. Plant and Cell Physiology 62, 942–947. 33836084 10.1093/pcp/pcab051

[CIT0241] Wang H , Liu C, Cheng J, Liu J, Zhang L, He C, Shen W-H, Jin H, Xu L, Zhang Y. 2016. Arabidopsis flower and embryo developmental genes are repressed in seedlings by different combinations of polycomb group proteins in association with distinct sets of cis-regulatory elements. PLoS Genetics 12, e1005771. 26760036 10.1371/journal.pgen.1005771 PMC4711971

[CIT0242] Wang J , Tian C, Zhang C, Shi B, Cao X, Zhang T-Q, Zhao Z, Wang J-W, Jiao Y. 2017. Cytokinin signaling activates WUSCHEL expression during axillary meristem initiation. The Plant Cell 29, 1373–1387. 28576845 10.1105/tpc.16.00579 PMC5502442

[CIT0243] Wang J-L , Di D-W, Luo P, Zhang L, Li X-F, Guo G-Q, Wu L. 2022. The roles of epigenetic modifications in the regulation of auxin biosynthesis. Frontiers in Plant Science 13, 959053. 36017262 10.3389/fpls.2022.959053 PMC9396225

[CIT0244] Wang L , Zhang Z, Zhang F, Shao Z, Zhao B, Huang A, Tran J, Hernandez FV, Qiao H. 2021. EIN2-directed histone acetylation requires EIN3-mediated positive feedback regulation in response to ethylene. The Plant Cell 33, 322–337. 33793786 10.1093/plcell/koaa029 PMC8136887

[CIT0245] Wang P , Abid MA, Qanmber G, et al. 2022. Photomorphogenesis in plants: the central role of phytochrome interacting factors (PIFs). Environmental and Experimental Botany 194, 104704.

[CIT0246] Wang R , Estelle M. 2014. Diversity and specificity: auxin perception and signaling through the TIR1/AFB pathway. Current Opinion in Plant Biology 21, 51–58. 25032902 10.1016/j.pbi.2014.06.006 PMC4294414

[CIT0247] Wang S , Li Y, Gan Y, Zhou H, Wang R. 2022. Labeling and quantitative analysis of i6A-incorporated RNA via in-situ azidation of prenyl functionality and click reaction. Tetrahedron Letters 100, 153873.

[CIT0248] Wang X , Chen C, He C, Chen D, Yan W. 2022. Mapping open chromatin by ATAC-seq in bread wheat. Frontiers in Plant Science 13, 1074873. 36466281 10.3389/fpls.2022.1074873 PMC9709403

[CIT0249] Wang X , Ding J, Lin S, Liu D, Gu T, Wu H, Trigiano RN, McAvoy R, Huang J, Li Y. 2020. Evolution and roles of cytokinin genes in angiosperms 2: do ancient CKXs play housekeeping roles while non-ancient CKXs play regulatory roles? Horticulture Research 7, 29. 32140238 10.1038/s41438-020-0246-z PMC7049301

[CIT0250] Wang X , Gao J, Gao S, Li Z, Kuai B, Ren G. 2019. REF6 promotes lateral root formation through de-repression of PIN1/3/7 genes. Journal of Integrative Plant Biology 61, 383–387. 30267471 10.1111/jipb.12726

[CIT0251] Wang Y , Gu X, Yuan W, Schmitz RJ, He Y. 2014. Photoperiodic control of the floral transition through a distinct polycomb repressive complex. Developmental Cell 28, 727–736. 24613395 10.1016/j.devcel.2014.01.029

[CIT0252] Wang Z , Cao H, Sun Y, et al. 2013. Arabidopsis paired amphipathic helix proteins SNL1 and SNL2 redundantly regulate primary seed dormancy via abscisic acid–ethylene antagonism mediated by histone deacetylation. The Plant Cell 25, 149–166. 23371947 10.1105/tpc.112.108191 PMC3584531

[CIT0253] Wardenaar R , Liu H, Colot V, Colome-Tatche M, Johannes F. 2013. Evaluation of MeDIP-chip in the context of whole-genome bisulfite sequencing (WGBS-Seq) in Arabidopsis. Methods in Molecular Biology 1067, 203–224. 23975794 10.1007/978-1-62703-607-8_13

[CIT0254] Weaver LM , Gan S, Quirino B, Amasino RM. 1998. A comparison of the expression patterns of several senescence-associated genes in response to stress and hormone treatment. Plant Molecular Biology 37, 455–469. 9617813 10.1023/a:1005934428906

[CIT0255] Wei L , Gu L, Song X, et al. 2014. Dicer-like 3 produces transposable element-associated 24-nt siRNAs that control agricultural traits in rice. Proceedings of the National Academy of Sciences, USA 111, 3877–3882. 10.1073/pnas.1318131111 PMC3956178 24554078

[CIT0256] Wei X , Wang W, Xu P, Wang W, Guo T, Kou S, Liu M, Niu Y, Yang H-Q, Mao Z. 2021. Phytochrome B interacts with SWC6 and ARP6 to regulate H2A.Z deposition and photomorphogensis in Arabidopsis. Journal of Integrative Plant Biology 63, 1133–1146. 33982818 10.1111/jipb.13111

[CIT0257] Wei Z , Li Y, Ali F, Wang Y, Liu J, Yang Z, Wang Z, Xing Y, Li F. 2022. Transcriptomic analysis reveals the key role of histone deacetylation via mediating different phytohormone signalings in fiber initiation of cotton. Cell & Bioscience 12, 107. 35831870 10.1186/s13578-022-00840-4 PMC9277824

[CIT0258] Weijers D , Schlereth A, Ehrismann JS, Schwank G, Kientz M, Jürgens G. 2006. Auxin triggers transient local signaling for cell specification in Arabidopsis embryogenesis. Developmental Cell 10, 265–270. 16459305 10.1016/j.devcel.2005.12.001

[CIT0259] Wein S , Andrews B, Sachsenberg T, Santos-Rosa H, Kohlbacher O, Kouzarides T, Garcia BA, Weisser H. 2020. A computational platform for high-throughput analysis of RNA sequences and modifications by mass spectrometry. Nature Communications 11, 926. 10.1038/s41467-020-14665-7 PMC7026122 32066737

[CIT0260] Weiste C , Dröge-Laser W. 2014. The Arabidopsis transcription factor bZIP11 activates auxin-mediated transcription by recruiting the histone acetylation machinery. Nature Communications 5, 3883. 10.1038/ncomms4883 24861440

[CIT0261] Weiste C , Pedrotti L, Selvanayagam J, Muralidhara P, Fröschel C, Novák O, Ljung K, Hanson J, Dröge-Laser W. 2017. The Arabidopsis bZIP11 transcription factor links low-energy signalling to auxin-mediated control of primary root growth. PLoS Genetics 13, e1006607. 28158182 10.1371/journal.pgen.1006607 PMC5315408

[CIT0262] Werner S , Bartrina I, Schmülling T. 2021. Cytokinin regulates vegetative phase change in *Arabidopsis thaliana* through the miR172/TOE1–TOE2 module. Nature Communications 12, 5816. 10.1038/s41467-021-26088-z PMC8492644 34611150

[CIT0263] Winter D , Vinegar B, Nahal H, Ammar R, Wilson GV, Provart NJ. 2007. An ‘Electronic Fluorescent Pictograph’ browser for exploring and analyzing large-scale biological data sets. PLoS One 2, e718. 17684564 10.1371/journal.pone.0000718 PMC1934936

[CIT0264] Wood CC , Robertson M, Tanner G, Peacock WJ, Dennis ES, Helliwell CA. 2006. The *Arabidopsis thaliana* vernalization response requires a polycomb-like protein complex that also includes VERNALIZATION INSENSITIVE 3. Proceedings of the National Academy of Sciences, USA 103, 14631–14636. 10.1073/pnas.0606385103 PMC1600011 16983073

[CIT0265] Wu B , Meng J, Liu H, et al. 2023. Suppressing a phosphohydrolase of cytokinin nucleotide enhances grain yield in rice. Nature Genetics 55, 1381–1389. 37500729 10.1038/s41588-023-01454-3

[CIT0266] Wu K , Wang S, Song W, et al. 2020. Enhanced sustainable green revolution yield via nitrogen-responsive chromatin modulation in rice. Science 367, eaaz2046. 32029600 10.1126/science.aaz2046

[CIT0267] Wu K , Zhang L, Zhou C, Yu C-W, Chaikam V. 2008. HDA6 is required for jasmonate response, senescence and flowering in Arabidopsis. Journal of Experimental Botany 59, 225–234. 18212027 10.1093/jxb/erm300

[CIT0268] Wu L , Luo P, Di D-W, et al. 2015. Forward genetic screen for auxin-deficient mutants by cytokinin. Scientific Reports 5, 11923. 26143750 10.1038/srep11923 PMC4491711

[CIT0269] Wu L-Y , Shang G-D, Wang F-X, Gao J, Wan M-C, Xu Z-G, Wang J-W. 2022. Dynamic chromatin state profiling reveals regulatory roles of auxin and cytokinin in shoot regeneration. Developmental Cell 57, 526–542. 35063083 10.1016/j.devcel.2021.12.019

[CIT0270] Wu M-F , Yamaguchi N, Xiao J, Bargmann B, Estelle M, Sang Y, Wagner D. 2015. Auxin-regulated chromatin switch directs acquisition of flower primordium founder fate. eLife 4, e09269. 26460543 10.7554/eLife.09269 PMC4600763

[CIT0271] Wu W , Du K, Kang X, Wei H. 2021. The diverse roles of cytokinins in regulating leaf development. Horticulture Research 8, 118. 34059666 10.1038/s41438-021-00558-3 PMC8167137

[CIT0272] Wyrzykowska A , Bielewicz D, Plewka P, Sołtys-Kalina D, Wasilewicz-Flis I, Marczewski W, Jarmolowski A, Szweykowska-Kulinska Z. 2022. The MYB33, MYB65, and MYB101 transcription factors affect Arabidopsis and potato responses to drought by regulating the ABA signaling pathway. Physiologia Plantarum 174, e13775. 36050907 10.1111/ppl.13775 PMC9828139

[CIT0273] Xie Y , Zhang Y, Han J, et al. 2018. The intronic cis element SE1 recruits trans-acting repressor complexes to repress the expression of ELONGATED UPPERMOST INTERNODE1 in rice. Molecular Plant 11, 720–735. 29524649 10.1016/j.molp.2018.03.001

[CIT0274] Xu M , Li X, Xie W, Lin C, Wang Q, Tao Z. 2023. ETHYLENE INSENSITIVE3/EIN3-LIKE1 modulate FLOWERING LOCUS C expression via histone demethylase interaction. Plant Physiology 192, 2290–2300. 36852894 10.1093/plphys/kiad131 PMC10315263

[CIT0275] Xu Y , Gan E-S, Zhou J, Wee W-Y, Zhang X, Ito T. 2014. Arabidopsis MRG domain proteins bridge two histone modifications to elevate expression of flowering genes. Nucleic Acids Research 42, 10960–10974. 25183522 10.1093/nar/gku781 PMC4176166

[CIT0276] Xu Y , Prunet N, Gan E-S, et al. 2018. SUPERMAN regulates floral whorl boundaries through control of auxin biosynthesis. The EMBO Journal 37, e97499. 29764982 10.15252/embj.201797499 PMC5983216

[CIT0277] Xue M , Zhang H, Zhao F, Zhao T, Li H, Jiang D. 2021. The INO80 chromatin remodeling complex promotes thermomorphogenesis by connecting H2A.Z eviction and active transcription in Arabidopsis. Molecular Plant 14, 1799–1813. 34242850 10.1016/j.molp.2021.07.001

[CIT0278] Yadav VK , Santos-González J, Köhler C. 2021. INT-Hi-C reveals distinct chromatin architecture in endosperm and leaf tissues of Arabidopsis. Nucleic Acids Research 49, 4371–4385. 33744975 10.1093/nar/gkab191 PMC8096224

[CIT0279] Yamaguchi N. 2021. Removal of H3K27me3 by JMJ proteins controls plant development and environmental responses in Arabidopsis. Frontiers in Plant Science 12, 687416. 34220908 10.3389/fpls.2021.687416 PMC8248668

[CIT0280] Yamoune A , Cuyacot AR, Zdarska M, Hejatko J. 2021. Hormonal orchestration of root apical meristem formation and maintenance in Arabidopsis. Journal of Experimental Botany 72, 6768–6788. 34343283 10.1093/jxb/erab360

[CIT0281] Yamoune A , Zdarska M, Depaepe T, et al. 2023. Cytokinins regulate spatially-specific ethylene production to control root growth in Arabidopsis. bioRxiv. 10.1101/2023.01.07.522790. [Preprint]. PMC11589326 38961625

[CIT0282] Yan W , Chen D, Smaczniak C, Engelhorn J, Liu H, Yang W, Graf A, Carles CC, Zhou D-X, Kaufmann K. 2018. Dynamic and spatial restriction of Polycomb activity by plant histone demethylases. Nature Plants 4, 681–689. 30104650 10.1038/s41477-018-0219-5

[CIT0283] Yang J , Cho L-H, Yoon J, et al. 2019. Chromatin interacting factor OsVIL2 increases biomass and rice grain yield. Plant Biotechnology Journal 17, 178–187. 29851259 10.1111/pbi.12956 PMC6330541

[CIT0284] Yang S , Li C, Zhao L, et al. 2015. The Arabidopsis SWI2/SNF2 chromatin remodeling ATPase BRAHMA targets directly to PINs and is required for root stem cell niche maintenance. The Plant Cell 27, 1670–1680. 25991732 10.1105/tpc.15.00091 PMC4498203

[CIT0285] Ying Y-L , Hu Z-L, Zhang S, Qing Y, Fragasso A, Maglia G, Meller A, Bayley H, Dekker C, Long Y-T. 2022. Nanopore-based technologies beyond DNA sequencing. Nature Nanotechnology 17, 1136–1146. 10.1038/s41565-022-01193-2 36163504

[CIT0286] Yu Z , Zhang F, Friml J, Ding Z. 2022. Auxin signaling: research advances over the past 30 years. Journal of Integrative Plant Biology 64, 371–392. 35018726 10.1111/jipb.13225

[CIT0287] Zachau HG , Dütting B, Feldmann H. 1966. The structures of two serine transfer ribonucleic acids. Hoppe-Seyler’s Zeitschrift für physiologische Chemie 347, 212–235. 5991670 10.1515/bchm2.1966.347.1.212

[CIT0288] Zander M , Willige BC, He Y, et al. 2019. Epigenetic silencing of a multifunctional plant stress regulator. eLife 8, e47835. 31418686 10.7554/eLife.47835 PMC6739875

[CIT0289] Zhang C , Jian M, Li W, Yao X, Tan C, Qian Q, Hu Y, Liu X, Hou X. 2023. Gibberellin signaling modulates flowering via the DELLA–BRAHMA–NF-YC module in Arabidopsis. The Plant Cell 35, 3470–3484. 37294919 10.1093/plcell/koad166 PMC10473208

[CIT0290] Zhang D , Jing Y, Jiang Z, Lin R. 2014. The chromatin-remodeling factor PICKLE integrates brassinosteroid and gibberellin signaling during skotomorphogenic growth in Arabidopsis. The Plant Cell 26, 2472–2485. 24920333 10.1105/tpc.113.121848 PMC4114946

[CIT0291] Zhang F , Qi B, Wang L, Zhao B, Rode S, Riggan ND, Ecker JR, Qiao H. 2016. EIN2-dependent regulation of acetylation of histone H3K14 and non-canonical histone H3K23 in ethylene signalling. Nature Communications 7, 13018. 10.1038/ncomms13018 PMC5063967 27694846

[CIT0292] Zhang F , Wang L, Ko EE, Shao K, Qiao H. 2018. Histone deacetylases SRT1 and SRT2 interact with ENAP1 to mediate ethylene-induced transcriptional repression. The Plant Cell 30, 153–166. 29298835 10.1105/tpc.17.00671 PMC5810571

[CIT0293] Zhang F , Wang L, Qi B, Zhao B, Ko EE, Riggan ND, Chin K, Qiao H. 2017. EIN2 mediates direct regulation of histone acetylation in the ethylene response. Proceedings of the National Academy of Sciences, USA 114, 10274–10279. 10.1073/pnas.1707937114 PMC5617289 28874528

[CIT0294] Zhang H , Rider SD, Henderson JT, et al. 2008. The CHD3 remodeler PICKLE promotes trimethylation of histone H3 lysine 27. Journal of Biological Chemistry 283, 22637–22648. 18539592 10.1074/jbc.M802129200 PMC2504882

[CIT0295] Zhang L , Luo P, Bai J, et al. 2021. Function of histone H2B monoubiquitination in transcriptional regulation of auxin biosynthesis in Arabidopsis. Communications Biology 4, 1–8. 33589721 10.1038/s42003-021-01733-x PMC7884795

[CIT0296] Zhang T-Q , Lian H, Tang H, et al. 2015. An intrinsic microRNA timer regulates progressive decline in shoot regenerative capacity in plants. The Plant Cell 27, 349–360. 25649435 10.1105/tpc.114.135186 PMC4456919

[CIT0297] Zhang T-Q , Lian H, Zhou C-M, Xu L, Jiao Y, Wang J-W. 2017. A two-step model for de novo activation of WUSCHEL during plant shoot regeneration. The Plant Cell 29, 1073–1087. 28389585 10.1105/tpc.16.00863 PMC5466026

[CIT0298] Zhang W , Jiang J. 2015. Genome-wide mapping of DNase I hypersensitive sites in plants. Methods in Molecular Biology 1284, 71–89. 25757768 10.1007/978-1-4939-2444-8_4

[CIT0299] Zhang Y , Yin S, Tu Y, Mei H, Yang Y. 2020. A novel microRNA, SlymiR208, promotes leaf senescence via regulating cytokinin biosynthesis in tomato. Physiologia Plantarum 169, 143–155. 31985059 10.1111/ppl.13068

[CIT0300] Zhao H , Zhang W, Zhang T, Lin Y, Hu Y, Fang C, Jiang J. 2020. Genome-wide MNase hypersensitivity assay unveils distinct classes of open chromatin associated with H3K27me3 and DNA methylation in *Arabidopsis thaliana*. Genome Biology 21, 24. 32014062 10.1186/s13059-020-1927-5 PMC6996174

[CIT0301] Zhong M , Zeng B, Tang D, Yang J, Qu L, Yan J, Wang X, Li X, Liu X, Zhao X. 2021. The blue light receptor CRY1 interacts with GID1 and DELLA proteins to repress GA signaling during photomorphogenesis in Arabidopsis. Molecular Plant 14, 1328–1342. 33971366 10.1016/j.molp.2021.05.011

[CIT0302] Zhong S , Lin Z, Grierson D. 2008. Tomato ethylene receptor–CTR interactions: visualization of NEVER-RIPE interactions with multiple CTRs at the endoplasmic reticulum. Journal of Experimental Botany 59, 965–972. 18349053 10.1093/jxb/ern021

[CIT0303] Zhou C , Zhang L, Duan J, Miki B, Wu K. 2005. HISTONE DEACETYLASE19 is involved in jasmonic acid and ethylene signaling of pathogen response in Arabidopsis. The Plant Cell 17, 1196–1204. 15749761 10.1105/tpc.104.028514 PMC1087996

[CIT0304] Zhou Y , Tergemina E, Cui H, Förderer A, Hartwig B, Velikkakam James G, Schneeberger K, Turck F. 2017. Ctf4-related protein recruits LHP1–PRC2 to maintain H3K27me3 levels in dividing cells in *Arabidopsis thaliana*. Proceedings of the National Academy of Sciences, USA 114, 4833–4838. 10.1073/pnas.1620955114 PMC5422822 28428341

[CIT0305] Zhu Z , An F, Feng Y, et al. 2011. Derepression of ethylene-stabilized transcription factors (EIN3/EIL1) mediates jasmonate and ethylene signaling synergy in Arabidopsis. Proceedings of the National Academy of Sciences, USA 108, 12539–12544. 10.1073/pnas.1103959108 PMC3145709 21737749

[CIT0306] Zong W , Kim J, Bordiya Y, Qiao H, Sung S. 2022. ABA negatively regulates the Polycomb-mediated H3K27me3 through the PHD-finger protein, VIL1. New Phytologist 235, 1057–1069. 35403701 10.1111/nph.18156 PMC9673473

[CIT0307] Zou Y , Wang Y, Wang L, Yang L, Wang R, Li X. 2013. miR172b controls the transition to autotrophic development inhibited by ABA in Arabidopsis. PLoS One 8, e64770. 23717657 10.1371/journal.pone.0064770 PMC3662786

[CIT0308] Zürcher E , Müller B. 2016. Cytokinin synthesis, signaling, and function—advances and new insights. International Review of Cell and Molecular Biology 324, 1–38. 27017005 10.1016/bs.ircmb.2016.01.001

[CIT0309] Zwiewka M , Bilanovičová V, Seifu YW, Nodzyński T. 2019. The nuts and bolts of PIN auxin efflux carriers. Frontiers in Plant Science 10, 985. 31417597 10.3389/fpls.2019.00985 PMC6685051

