# Supplementary material for: Epigenetics and plant hormone dynamics: a functional and methodological perspective
Source: J Exp Bot. 2024 Feb 19;75(17):5267–94. doi: 10.1093/jxb/erae054 (PMC11389840; doi:10.1093/jxb/erae054)
Supplement: erae054_suppl_Supplementary_Tables_S1 [file erae054_suppl_supplementary_tables_s1.pdf]

Supplementary Table S1

Abscisic acid			
**Gene ID**	**Gene**	**Uniprot ID**	**Protein description**
At1g08260	*POL2A*	F4HW04	DNA polymerase epsilon catalytic subunit A
At1g14350	*MYB124*	Q94FL6	Transcription factor MYB124
At1g21970	*NFYB9*	Q9SFD8	Nuclear transcription factor Y subunit B-9
At1g63490	*JMJ17*	F4I240	Lysine-specific demethylase JMJ17
At1g75080	*BZR1*	Q8S307	Protein BRASSINAZOLE-RESISTANT 1
At2g23380	*CLF*	P93831	Histone-lysine N-methyltransferase CLF
At2g31650	*ATX1*	Q9C5X4	Histone H3-lysine(4) N-trimethyltransferase ATX1
At3g20810	*JMJ30*	Q8RWR1	Lysine-specific demethylase JMJ30
At3g18520	*HDA15*	Q8GXJ1	Histone deacetylase 15
At3g24650	*ABI3*	Q01593	B3 domain-containing transcription factor ABI3
At3g45880	*JMJ32*	Q0WVR4	Lysine-specific demethylase JMJ32
At3g48430	*REF6*	Q9STM3	Lysine-specific demethylase REF6
At4g00990	*JMJ27*	Q8VYB9	Lysine-specific demethylase JMJ27
At4g02020	*EZA1*	Q9ZSM8	Histone-lysine N-methyltransferase EZA1
At4g38130	*HDA19*	O22446	Histone deacetylase 19
At5g03280	*EIN2*	Q9S814	Ethylene-insensitive protein 2
At5g03740	*HDT3*	Q9LZR5	Histone deacetylase HDT3
At5g11530	*EMF1*	Q9LYD9	Protein EMBRYONIC FLOWER 1
At5g45890	*SAG12*	Q9FJ47	Senescence-specific cysteine protease SAG12
At5g51230	*EMF2*	Q8L6Y4	Polycomb group protein EMBRYONIC FLOWER 2
At5g63110	*HDA6*	Q9FML2	Histone deacetylase 6
*Os03g0307800	*EZ1*	Q10MI4	Histone-lysine N-methyltransferase EZ1
*Os06g0583400	*HDAC1*	Q7Y0Y8	Histone deacetylase 1
*Os03g0390600	*H3*	Q0JCT1	Histone H3.3

**Oryza sativa* orthologue

Cytokinins			
**Gene ID**	**Gene**	**Uniprot ID**	**Protein description**
At3g04240	*SEC*	Q9M8Y0	Probable UDP-N-acetylglucosamine--peptide N-acetylglucosaminyltransferase SEC
At3g12280	*RBR1*	Q9LKZ3	Retinoblastoma-related protein 1

At3g12380	*ARP5*	Q940Z2	Actin-related protein 5
At5g03280	*EIN2*	Q9S814	Ethylene-insensitive protein 2
At5g45890	*SAG12*	Q9FJ47	Senescence-specific cysteine protease SAG12
*Os05g0196500	*JMJ703*	Q53WJ1	Lysine-specific demethylase JMJ703

**Oryza sativa* orthologue

Auxin

**Gene ID**	**Gene**	**Uniprot ID**	**Protein description**
At1g14350	*MYB124*	Q94FL6	Transcription factor MYB124
At2g32700	*LUH*	O48847	Transcriptional corepressor LEUNIG_HOMOLOG
At2g33860	*ARF3*	O23661	Auxin response factor 3
At3g01460	*MBD9*	Q9SGH2	Methyl-CpG-binding domain-containing protein 9
At3g12280	*RBR1*	Q9LKZ3	Retinoblastoma-related protein 1
At3g18520	*HDA15*	Q8GXJ1	Histone deacetylase 15
At3g24650	*ABI3*	Q01593	B3 domain-containing transcription factor ABI3
At3g44600	*CYP71*	Q8W4D0	Peptidyl-prolyl cis-trans isomerase CYP71
At4g13520	*SMAP1*	Q9T0H2	Small acidic protein 1
At5g03280	*EIN2*	Q9S814	Ethylene-insensitive protein 2
At5g45890	*SAG12*	Q9FJ47	Senescence-specific cysteine protease SAG12
At5g60450	*ARF4*	Q9ZTX9	Auxin response factor 4
*Os10g0415900	*GCN5*	Q338B9	Histone acetyltransferase GCN5

**Oryza sativa* orthologue

Ethylene

**Gene ID**	**Gene**	**Uniprot ID**	**Protein description**
At1g54490	*XRN4*	Q9FQ04	5'-3' exoribonuclease 4
At2g28290	*SYD*	F4IHS2	Chromatin structure-remodeling complex protein SYD
At3g11100	*ENAP1*	Q8VZI9	Trihelix transcription factor ENAP1
At3g12380	*ARP5*	Q940Z2	Actin-related protein 5
At3g20770	*EIN3*	O24606	Protein ETHYLENE INSENSITIVE 3
At3g56400	*WRKY70*	Q9LY00	Probable WRKY transcription factor 70
At3g48430	*REF6*	Q9STM3	Lysine-specific demethylase REF6
At3g57300	*INO80*	Q8RXS6	Chromatin-remodeling ATPase INO80

At4g38130	*HDA19*	O22446	Histone deacetylase 19
At4g38495	*EEN*	Q8RWS0	Protein ENHANCER OF ETHYLENE INSENSITIVITY 6
At5g03280	*EIN2*	Q9S814	Ethylene-insensitive protein 2
At5g45890	SAG12	Q9FJ47	Senescence-specific cysteine protease SAG12
At5g63110	HDA6	Q9FML2	Histone deacetylase 6
*Os04g0271000	SRT1	Q7XWV4	NAD-dependent protein deacetylase SRT1
*Os05g0121600	AP2-1	Q2TQ34	APETALA2-like protein 1

**Oryza sativa* orthologue

Gibberellins			
**Gene ID**	**Gene**	**Uniprot ID**	**Protein description**
At3g04240	SEC	Q9M8Y0	Probable UDP-N-acetylglucosamine--peptide N-acetylglucosaminyltransferase SEC
At5g06550	JMJ22	Q67XX3	Arginine-specific demethylase JMJ22
At5g63080	JMJ20	Q67ZB6	Arginine-specific demethylase JMJ20
*Solyc06g074790.1.1	H2B-1	O65821	Histone H2B.1
*Solyc11g066430.1.1	H2B-2	O65818	Histone H2B.2
*Solyc05g055440.1.1	H2B-3	O65819	Histone H2B.3

* *Solanum lycopersicum* orthologue
